# A redox-active diborane platform performs C(sp^3^)–H activation and nucleophilic substitution reactions†
†Electronic supplementary information (ESI) available. CCDC 1819687–1819695. For ESI and crystallographic data in CIF or other electronic format see DOI: 10.1039/c8sc00743h


**DOI:** 10.1039/c8sc00743h

**Published:** 2018-03-19

**Authors:** Thomas Kaese, Timo Trageser, Hendrik Budy, Michael Bolte, Hans-Wolfram Lerner, Matthias Wagner

**Affiliations:** a Institut für Anorganische und Analytische Chemie , Goethe-Universität Frankfurt , Max-von-Laue-Straße 7 , D-60438 Frankfurt am Main , Germany . Email: matthias.wagner@chemie.uni-frankfurt.de

## Abstract

Organoboranes are among the most versatile and widely used reagents in synthetic chemistry. A significant further expansion of their application spectrum would be achievable if boron-containing reactive intermediates capable of inserting into C–H bonds or performing nucleophilic substitution reactions were readily available. However, current progress in the field is still hampered by a lack of universal design concepts and mechanistic understanding. Herein we report that the doubly arylene-bridged diborane(6) **1**H_2_ and its B=B-bonded formal deprotonation product Li_2_[**1**] can activate the particularly inert C(sp^3^)–H bonds of added H_3_CLi and H_3_CCl, respectively. The first case involves the attack of [H_3_C]^–^ on a Lewis-acidic boron center, whereas the second case follows a polarity-inverted pathway with nucleophilic attack of the B=B double bond on H_3_CCl. Mechanistic details were elucidated by means of deuterium-labeled reagents, a radical clock, α,ω-dihaloalkane substrates, the experimental identification of key intermediates, and quantum-chemical calculations. It turned out that both systems, H_3_CLi/**1**H_2_ and H_3_CCl/Li_2_[**1**], ultimately funnel into the same reaction pathway, which likely proceeds past a borylene-type intermediate and requires the cooperative interaction of both boron atoms.

## Abstract 2

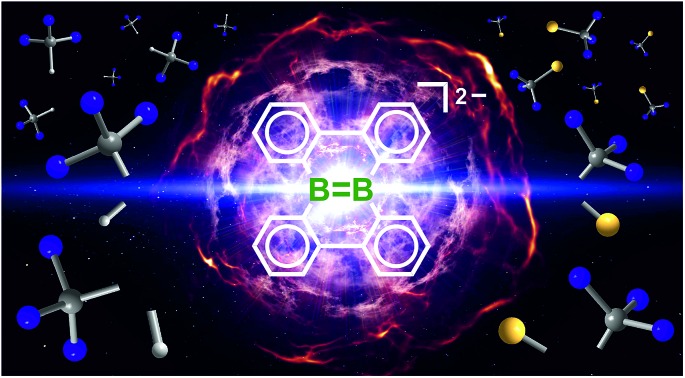
Targeted C(sp^3^)–H activation or nucleophilic substitution reactions have been achieved through the interaction of a diborane dianion with haloalkanes.

## Introduction

For decades, organoboranes remained limited to a passive role as reagents in organic synthesis, where boryl substituents either serve as placeholders for other functional groups (*e.g.*, halides, hydroxy, and amino groups),^1^ or are involved in Pd-catalyzed C–C-coupling reactions.^2^ Another useful asset, the potential of boron compounds to actively promote the cleavage of element–element bonds, lay dormant until the concepts of “Boron Lewis-acid catalysis“^3^–^6^ and “Frustrated Lewis pairs”^7^–^9^ were introduced about 15 years ago. Since then, it became increasingly apparent that appropriately selected main group compounds can rival transition metal complexes in mediating the transformation of organic substrates.

Certain organoboranes are catalytically active not only in their Lewis-acidic neutral forms, but also in their exhaustively reduced states. As prominent examples, 9,10-dihydro-9,10-diboraanthracenes (DBAs) catalyze inverse electron-demand Diels–Alder reactions of 1,2-diazines^3^ as well as the dehydrogenation of ammonia-borane.^5^ Upon reduction, the corresponding [DBA]^2–^ anions readily add C(sp)–H or H–H bonds across the two boron atoms; the latter reaction can be exploited for the economic conversion of chlorosilanes into hydrosilanes.^10^,^11^

**Scheme 1:**
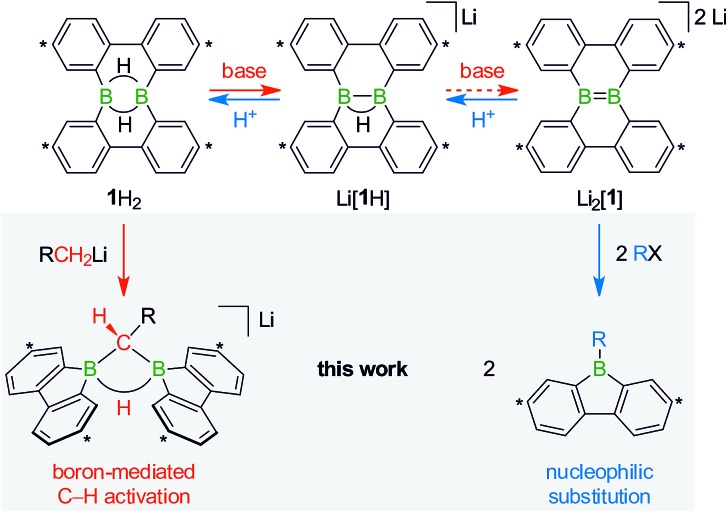
The members of the triad **1**H_2_/Li[**1**H]/Li_2_[**1**] are linked through redox processes as well as protonation/deprotonation reactions. Treatment of **1**H_2_ with RCH_2_Li leads to C(sp^3^)–H activations and skeletal rearrangements to furnish 1,1-bis(9-borafluorenyl)methanes (together with Li[**1**H]; R = H, C_3_H_7_). The addition of haloalkanes RX to Li_2_[**1**] results in nucleophilic substitution reactions and again skeletal rearrangements to afford 9-*R*-9-borafluorenes (in some cases accompanied by C(sp^3^)–H activations; X = Cl, Br, I). Carbon atoms marked with asterisks bear *t*Bu substituents.

With the triad **1**H_2_/Li[**1**H]/Li_2_[**1**] (Scheme 1), we recently developed a system of ditopic boranes, which is comparable to the DBA/[DBA]^2–^ pair, because it encompasses a Lewis-acidic (**1**H_2_) together with a dianionic species ([**1**]^2–^). As a decisive difference, however, the boron atoms in [DBA]^2–^ are linked by two *o*-phenylene rings, whereas in [**1**]^2–^ they are directly connected by a double bond. Both systems thus possess different frontier orbitals and should exhibit different reactivities.

The anions [**1**H]^–^ and [**1**]^2–^ are accessible in good yields *via* alkali-metal reduction of **1**H_2_.^12^–^14^ Stepwise protonation with ethereal HCl cleanly takes [**1**]^2–^ back to [**1**H]^–^ and finally **1**H_2_.^14^ The reverse deprotonation reaction of **1**H_2_ to afford [**1**H]^–^ is also quantitative, provided that the sterically demanding bases (Me_3_Si)_2_NLi and (Me_3_Si)_3_CLi are used. In case of the smaller *n*BuLi, the deprotonation reaction (20%) is accompanied by the formation of an anionic diborylmethane featuring a boron-bridging hydrogen atom (30%; Scheme 1, R = C_3_H_7_).^14^ These remarkable results immediately raise the following questions: (i) can **1**H_2_ activate C(sp^3^)–H bonds of added alkyllithium reagents RCH_2_Li? (ii) Will [**1**]^2–^ show nucleophilic behavior also toward electrophiles other than the proton (*i.e.*, RX)?

**Scheme 2:**
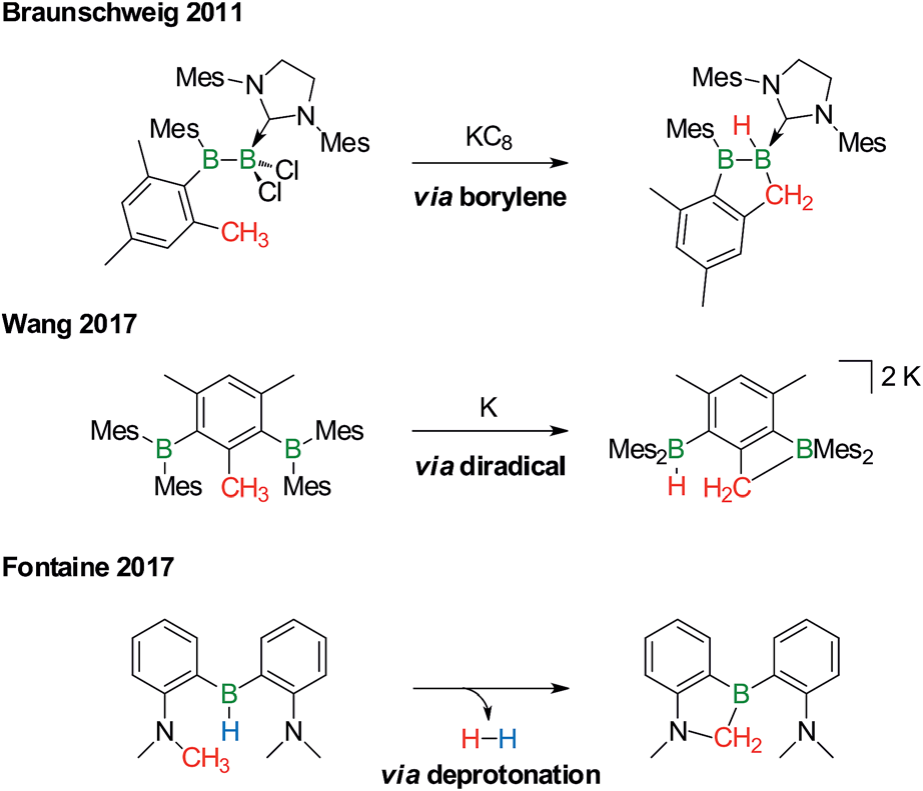
Selected examples of transition metal-free intramolecular C(sp^3^)–H activations through borylene (top), diradical (middle), and deprotonation reactions (bottom). Mes = 2,4,6-(H_3_C)_3_C_6_H_2_.

Derivatization reactions of the inert C(sp^3^)–H bond are as topical as they are challenging – even if transition-metal catalysts are present.^15^–^18^ The few known boron-promoted examples fall into the three categories compiled in Scheme 2: (1) Braunschweig performed the reductive dechlorination of a dichloroborane precursor to generate an intermediate borylene, which inserted into the H_3_C group of a nearby mesityl substituent.^19^ (2) Wang *et al.* observed hydrogen-atom abstraction from a H_3_C group with concomitant formation of B–H and B–C bonds when they reduced 2,6-bis(BMes_2_)mesitylene to its diradical state.^20^ (3) Fontaine exploited an intramolecular deprotonation step on an FLP platform to establish an NCH_2_–B bond; subsequent H_2_ liberation provided the necessary thermodynamic driving force.^21^

The umpolung of carbon electrophiles through their conversion in, *e.g.*, nucleophilic organolithium or Grignard reagents was one of the most important breakthroughs for the laboratory synthesis of organic compounds. A comparably high impact on the future progress of boron chemistry can be expected from the development of efficient tools to accomplish a polarity inversion of the intrinsically electrophilic boron center.^22^

**Fig. 1:**
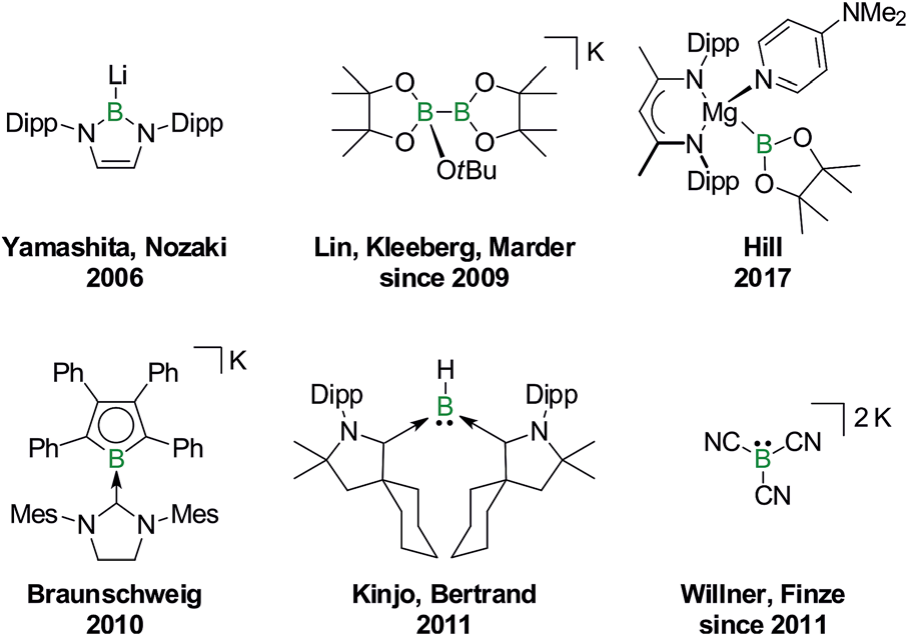
Selected isolable boron compounds showing formal nucleophilic behavior. Dipp = 2,6-(*i*Pr)_2_C_6_H_3_.

In 2006, Yamashita and Nozaki pioneered the field of nucleophilic boron compounds by disclosing a lithium boryl isostere of stable *N*-heterocyclic carbenes (NHCs; Fig. 1).^23^ More than 10 years later, Hill expanded the class of compounds to include an isolable magnesium pinacolatoboryl complex.^24^ In the intervening period, a wealth of chemistry had already been developed based on the *in situ* generation of pinacolatoboryl nucleophiles *via* the alkoxide-induced heterolytic cleavage of bis(pinacolato)diboron (Lin, Kleeberg, Marder and others).^25^ Boryl nucleophiles can also be stabilized through π delocalization of the boron lone pair, as exemplified by Braunschweig's NHC-adduct of a borolyl salt (which may in fact react *via* radical pathways),^26^ the cyclic (alkyl)(amino)carbene-coordinated BH fragment of Kinjo/Bertrand,^27^ as well as Willner's/Finze's alkali metal tricyanoborate (Fig. 1).^28^

Before the background provided by the literature and our own previous results, we regarded the triad **1**H_2_/Li[**1**H]/Li_2_[**1**] as a perfect platform for further studies into boron-promoted C–H-activation processes and boron-centered nucleophiles. Herein we present evidence that the reactions of **1**H_2_ with RCH_2_Li indeed proceed through C(sp^3^)–H-cleavage steps and that the boron-bridging H atoms in the diborylmethane products stem from the organolithium reagents and are not remains of **1**H_2_ (*cf.*Scheme 1; R = H, C_3_H_7_). We also show that the B=B double bond of the dianion [**1**]^2–^ behaves as a closed-shell nucleophile toward organohalides and that specifically H_3_CCl/Li_2_[**1**] and H_3_CLi/**1**H_2_ funnel into the same reaction channel. When H_3_CCl is replaced by an excess of H_3_C–I, C–H-activation is completely suppressed by a second nucleophilic substitution reaction to afford 2 equiv. of 9-methyl-9-borafluorene (Scheme 1; R = H_3_C). Employing α,ω-dihaloalkanes X(CH_2_)_*n*_X and Li_2_[**1**], we gained further insight into the competition between the nucleophilic substitution and C–H-activation scenarios as well as the cooperativity of the two adjacent boron centers (X = Cl, Br).

## Results and discussion

We started our study by addressing the question: why and how does the reaction of **1**H_2_ with *n*BuLi furnish not only the deprotonation product Li[**1**H], but also the diborylmethane-hydride adduct shown in Scheme 1 (R = C_3_H_7_)?

**Scheme 3:**
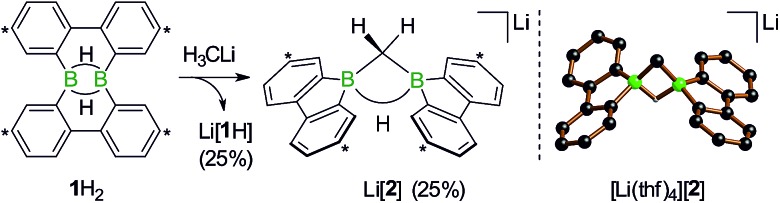
The addition of H_3_CLi to **1**H_2_ furnishes the C–H activation product Li[**2**] together with the deprotonated compound Li[**1**H] (left; carbon atoms marked with asterisks bear *t*Bu substituents). Molecular structure of [Li(thf)_4_][**2**] in the solid state (right). The solvent-separated [Li(thf)_4_]^+^ cation, all *t*Bu groups, and all CH atoms are omitted for clarity. Selected atom···atom distance [Å] and bond angle [°]: B···B = 1.974(6); B–CH_2_–B = 76.8(3).

First, we confirmed that a simplified system using H_3_CLi in place of *n*BuLi maintains the same general reactivity (Scheme 3). From equimolar mixtures of **1**H_2_ and H_3_CLi, the products Li[**1**H] and Li[**2**] are formed in slightly varying relative amounts but constant combined yields of close to 50% (the analogous finding holds for the *n*BuLi case). The ^1^H NMR spectroscopic monitoring of the reaction in a sealed NMR tube (THF-*d*_8_, room temperature) showed no free H_2_ (*δ* 4.55 ppm),^29^ which is an important observation considering that the starting materials **1**H_2_ and H_3_CLi contain a sum of five BHB/H_3_CLi protons, of which only three remain in the product Li[**2**].

**Scheme 4:**
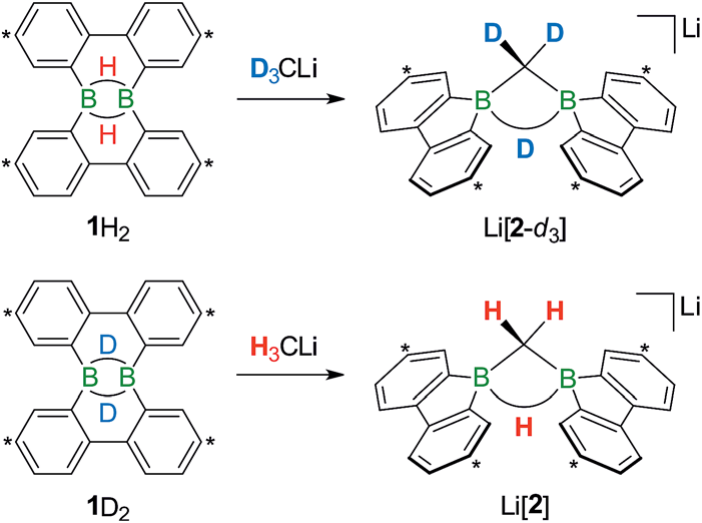
The reactions D_3_CLi/**1**H_2_ (top) or H_3_CLi/**1**D_2_ (bottom) give the C–D- or C–H-activation products Li[**2**-*d*_3_] or Li[**2**], respectively. Carbon atoms marked with asterisks bear *t*Bu substituents.

Deuterium-labeling experiments with D_3_CLi/**1**H_2_ or H_3_CLi/**1**D_2_ combinations furnished isotopically pure Li[**2**-*d*_3_] or Li[**2**], respectively (Scheme 4). Thus, not only the methylene linker (*δ*(^1^H) 0.49 ppm, d), but also the boron-bridging hydrogen atom (*δ*(^1^H) 1.94 ppm, br) in Li[**2**] originate from the organolithium reagent. None of the two BHB atoms of **1**H_2_ is still present in the product Li[**2**-*d*_3_] (see the ESI† for more information). We also note the appearance of two sets of aryl-proton signals that neither belong to Li[**1**H] nor Li[**2**] (or their partly deuterated counterparts) and are consequently accountable for the missing 50% product yield (see below).

**Scheme 5:**
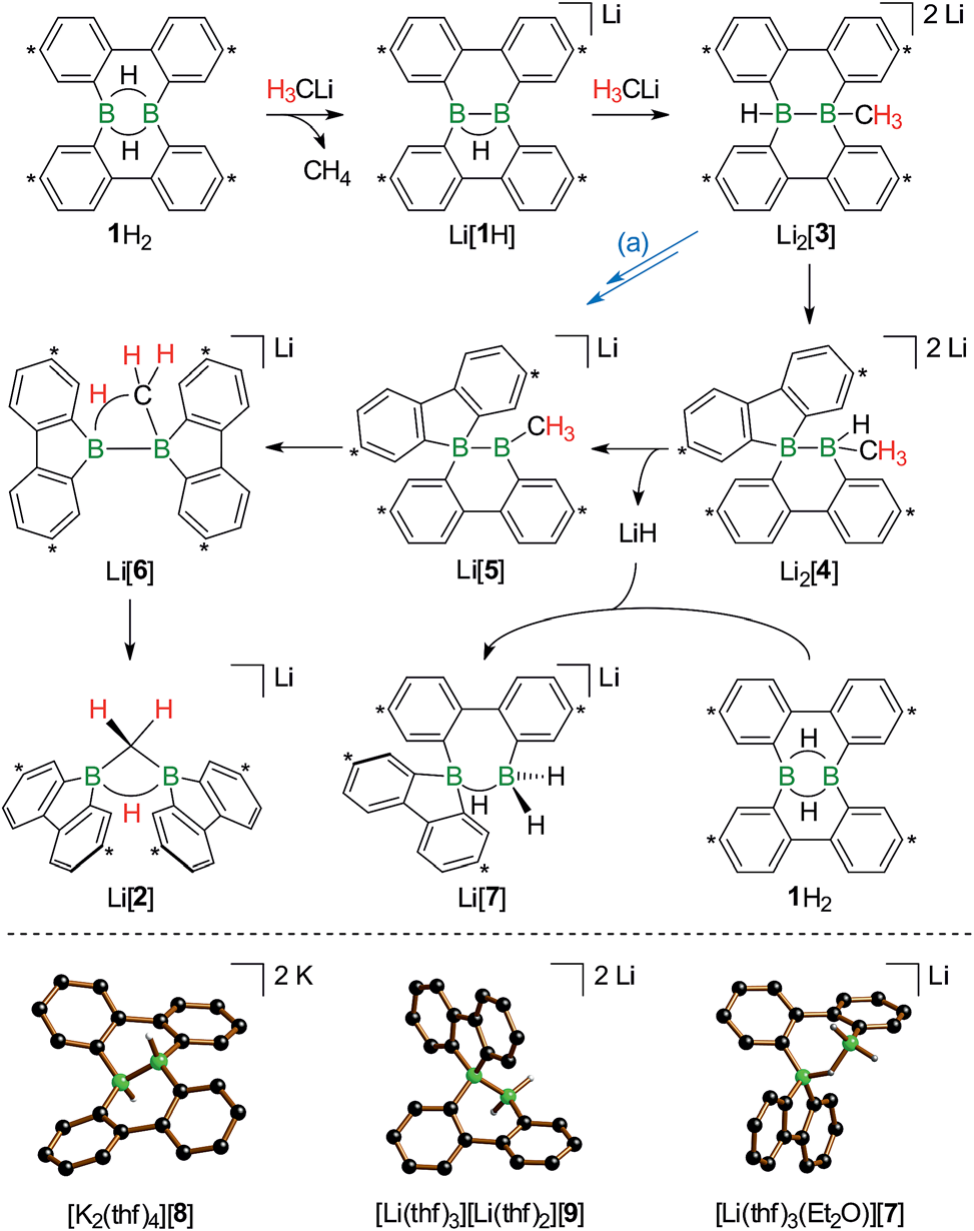
Proposed reaction mechanism explaining the formation of Li[**1**H], Li[**2**], and Li[**7**] from an equimolar mixture of H_3_CLi and **1**H_2_ (top; carbon atoms marked with asterisks bear *t*Bu substituents). The alternative pathway (a) leads from Li_2_[**3**] to Li[**5**], first *via* hydride elimination and second *via* a 1,2-phenyl shift. Molecular structures of [K_2_(thf)_4_][**8**], [Li(thf)_3_][Li(thf)_2_][**9**], and [Li(thf)_3_(Et_2_O)][**7**] in the solid state (bottom). The solvent-separated cations, all *t*Bu groups, and all CH atoms are omitted for clarity.

In the following, a plausible mechanistic model for the conversion of **1**H_2_ with H_3_CLi will be described (black arrows in Scheme 5), which accounts for all available experimental evidence. It explains (i) the C–H activation of [H_3_C]^–^, (ii) the fate of the boron-bonded hydrogen atoms of **1**H_2_, and (iii) the combined yield of only 50% for Li[**1**H] and Li[**2**]: similar to the case (Me_3_Si)_3_CLi/**1**H_2_, the reaction H_3_CLi/**1**H_2_ starts with the deprotonation of **1**H_2_ to afford Li[**1**H]. The byproduct CH_4_ was detected by ^1^H and ^13^C{^1^H} NMR spectroscopy; when D_3_CLi was employed as the Brønsted base, we instead observed the formation of D_3_CH (sealed NMR tubes; see the ESI† for more details).

Contrary to the case (Me_3_Si)_3_CLi/**1**H_2_, the reaction involving H_3_CLi does not necessarily stop at the stage of Li[**1**H], because the small [H_3_C]^–^ ion also has the potential to act as a Lewis base. Nucleophilic attack of H_3_CLi on a boron atom of Li[**1**H] establishes a B–CH_3_ bond and shifts the boron-bridging hydrogen atom to a terminal position. The structural motif of the resulting intermediate [**3**]^2–^ has precedence in the crystallographically characterized dianion [**8**]^2–^,^13^ which carries a further hydrogen atom rather than a boron-bonded methyl group (Scheme 5, top and bottom). Li_2_[**3**] rearranges to Li_2_[**4**] through a 1,2-phenyl shift, accompanied by a 1,2-hydride shift. Again, a comparable hydrogen-containing species Li_2_[**9**] exists (Scheme 5, bottom), and its molecular structure has been confirmed by X-ray analysis.^13^ Li_2_[**9**] can isomerize to Li_2_[FluB(H)–(H)BFlu] (BFlu = 9-borafluorenyl),^13^ thereby providing an example of a 1,2-phenyl/1,2-hydride-shift cascade closely related to the isomerization of Li_2_[**3**] to Li_2_[**4**]. The latter reaction continues with an LiH-elimination step to generate Li[**5**], which possesses a three-coordinate boron atom with a vacant p_z_ orbital and therefore easily undergoes a 1,2-phenyl shift to produce Li[**6**]. The anion [**6**]^–^ can be viewed as the [H_3_C]^–^ adduct of a diborane(4) containing two 9-borafluorene units that are linked by a B–B single bond. Only the sp^3^-hybridized boron atom has acquired an electron octet, however, also the B(sp^2^) center might gain some electron density from an agostic interaction with the methyl group and thereby reduce its strong Lewis acidity.^30^ Finally, this interaction turns into C–H-bond activation accompanied by B–B-bond cleavage and ultimately results in the formation of Li[**2**]. It is well known that B(sp^2^)–B(sp^3^) diboranes readily undergo B–B-bond heterolysis and thereby act as mild sources of nucleophilic boron.^31^ Moreover, the core parts of [**2**]^–^ and [**6**]^–^ are isoelectronic with protonated cyclopropane [C_3_H_7_]^+^. This cation has been thoroughly investigated by experimental^32^–^34^ and theoretical^35^,^36^ methods and found to be a highly fluctional system,^37^ which supports the idea of [**6**]^–^ rearranging to [**2**]^–^. At this stage, the dynamic behavior comes to an end, because, contrary to the case of [C_3_H_7_]^+^, the three corners of [**2**]^–^ are not equivalent and the BHB bridge should be thermodynamically favored over alternative BHC bridges.

**Fig. 2:**
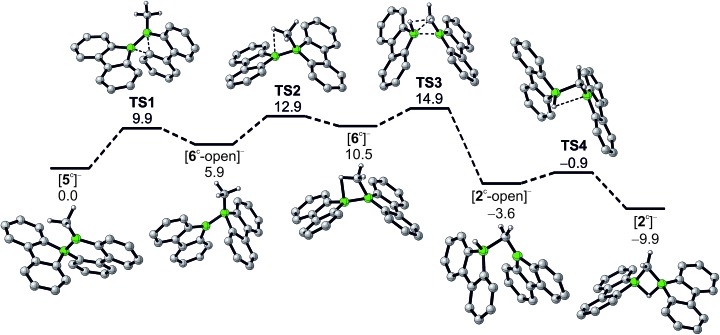
Reaction pathway for the conversion of [**5**^c^]^–^ to [**2**^c^]^–^, calculated at the PBE0D/TZVP level of theory with the SMD polarized continuum model for solvation in THF. Gibbs free energies at 298 K (Δ*G*) are given in kcal mol^–1^ relative to [**5**^c^]^–^.

In addition to the qualitative comparison with the all-carbon model system [C_3_H_7_]^+^, we studied the key C–H-activation step of the organoboron anion [**6**]^–^ by quantum-chemical calculations (Fig. 2). Apart from the Li^+^ counterion, which likely is solvent-separated in THF solution (*cf.* the solid-state structure of [Li(thf)_4_][**2**]; Scheme 3, right), we also omitted the *t*Bu substituents. The computed parent systems will be denoted with a superscript ‘c’ (*e.g.*, [**5**^c^]^–^ represents Li[**5**]). The 1,2-phenyl shift in [**5**^c^]^–^ proceeds *via***TS1** with an activation barrier of Δ*G*^‡^ = 9.9 kcal mol^–1^ and is endoergic by Δ*G*_R_ = 5.9 kcal mol^–1^. The resulting open-chain rearrangement product [**6**^c^-open]^–^ features a large B–B–CH_3_ bond angle of 121° and the vacant p_z_ orbital of the B(sp^2^) atom is oriented almost orthogonal to the B–CH_3_-bond vector, which precludes an agostic interaction in this isomer. To establish the B–H–C bridge proposed above, the tricoordinate borafluorene fragment must be rotated by approximately 70° and the B–B–CH_3_ bond angle contracted – ultimately to a value of 68° in the local-minimum structure [**6**^c^]^–^. The conversion of [**6**^c^-open]^–^ to the cyclic isomer [**6**^c^]^–^*via***TS2** (Δ*G*^‡^ = 7.0 kcal mol^–1^) is associated with a moderate energy penalty of Δ*G*_R_ = 4.6 kcal mol^–1^. The actual C–H-activation process involves the transition state **TS3** in which the B–B bond and one C–H bond are concertedly cleaved and a new B–C bond is formed (Δ*G*^‡^ = 4.4 kcal mol^–1^).

The primary, open-chain activation product [**2**^c^-open]^–^ is thermodynamically favored by –14.1 kcal mol^–1^ and –3.6 kcal mol^–1^ compared to [**6**^c^]^–^ and [**5**^c^]^–^, respectively. A further stabilization is achievable through rotation about a B–C bond and placement of the hydrogen atom into a boron-bridging position to obtain the final product [**2**^c^]^–^ (**TS4**: Δ*G*^‡^ = 2.7 kcal mol^–1^; Δ*G*_R_ = –6.3 kcal mol^–1^). In summary, the reaction cascade from [**5**^c^]^–^ to [**2**^c^]^–^ possesses an overall activation barrier of Δ*G*^‡^ = 14.9 kcal mol^–1^, which is easily surmountable at room temperature. An appreciable thermodynamic driving force is provided by the exergonicity of the [**2**^c^]^–^ formation (Δ*G*_R_ = –9.9 kcal mol^–1^).

**Scheme 6:**
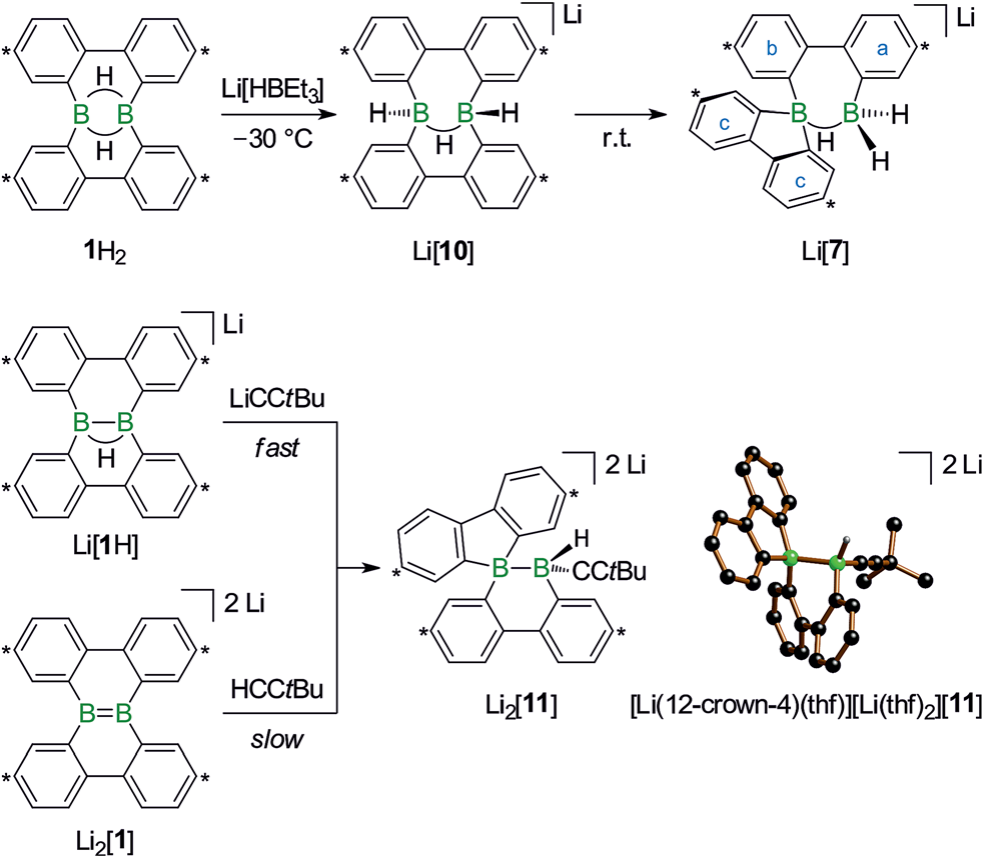
Reaction of **1**H_2_ with Li[HBEt_3_] at –30 °C to give Li[**10**], which isomerizes to Li[**7**] at room temperature (top). Compound Li_2_[**11**] forms in both reactions, *t*BuCCLi/Li[**1**H] and *t*BuCCH/Li_2_[**1**] (bottom; carbon atoms marked with asterisks bear *t*Bu substituents). Molecular structure of [Li(12-crown-4)(thf)][Li(thf)_2_][**11**] in the solid state. The solvent-separated cations, phenyl-bonded *t*Bu groups, and all CH atoms are omitted for clarity.

To experimentally substantiate the role of Li[**1**H] as the first intermediate along the pathway from **1**H_2_ to Li[**2**], we treated an isolated sample of Li[**1**H] with 1 equiv. of H_3_CLi in THF. Even though the reaction started as expected, it stopped at the stage of Li_2_[**4**] (which enabled us to record a ^1^H NMR spectrum of this compound). The elimination of LiH from Li_2_[**4**] is thus not a spontaneous process, but apparently requires a hydride-trapping reagent. Compound **1**H_2_ constitutes an ideal candidate for this purpose and, indeed, after the addition of 1 equiv. of **1**H_2_, Li_2_[**4**] quantitatively vanished and Li[**2**] formed instead. Moreover, we found two sets of proton resonances that are assignable to two isomeric hydride-trapping products of **1**H_2_ (*cf.* Li[**7**], Li[**10**]; Schemes 5 and 6).

As a caveat we emphasize that the reaction from **1**H_2_ to Li[**2**] may bypass the intermediate Li_2_[**4**] if hydride transfer from Li_2_[**3**] to **1**H_2_ is faster than the rearrangement from Li_2_[**3**] to Li_2_[**4**] (blue path (a) in Scheme 5). Arguments in favor of this alternative route include: (i) the 1,2-phenyl shift required to generate intermediate Li[**5**] should be more facile on a B(sp^2^)–B(sp^3^) rather than a B(sp^3^)–B(sp^3^) scaffold (*cf.* Li_2_[**3**] → Li_2_[**4**]; Scheme 5). (ii) Li_2_[**4**] was observed only when the reaction was started from Li[**1**H], *i.e.*, when the hydride trap **1**H_2_ was absent, thus rendering the blue path impassable.

After the above discussion of a plausible mechanistic picture underlying the overall reaction scenario, we now present analytical data of key intermediates and products. The reaction H_3_CLi/**1**H_2_ furnishes Li[**1**H] and Li[**2**] besides the isomeric hydride-trapping products Li[**7**] and Li[**10**]. The first species, Li[**1**H], is a known compound and therefore does not require further discussion.^14^ The second species, Li[**2**], is reminiscent of the published C–H-activation product obtained from the reaction *n*BuLi/**1**H_2_ (*cf.*Scheme 1, R = C_3_H_7_).^14^ The main difference between both compounds relates to the fact that Li[**2**] possesses an average *C*_2v_ symmetry in solution, whereas a pending C_3_H_7_ substituent reduces the symmetry to *C*_s_. Consequently, the ^1^H NMR spectrum of Li[**2**] contains only one set of signals for all four *t*Bu-C_6_H_3_ rings. The corresponding spectrum of its *C*_s_-symmetric congener features two sets of resonances,^14^ one of them with chemical shift values almost identical to those of Li[**2**] and thus likely assignable to those halves of the 9-borafluorene subunits, which point into the same direction as the proton residing on the methylene bridge. A similar interpretation is valid for the ^13^C{^1^H} NMR spectrum of Li[**2**]. Single crystals of [Li(thf)_4_][**2**] suitable for X-ray analysis were grown from THF-hexane (Scheme 3). Like its C_3_H_7_ derivative,^14^ [Li(thf)_4_][**2**] forms solvent-separated ion pairs in the crystal lattice, and all key geometric parameters of the two anions are identical within the experimental error margins. We also note a pleasingly good agreement between the experimentally determined structure of [**2**]^–^ and the computed structure of [**2**^c^]^–^ (*cf.* the ESI† for full details).

^1^H NMR spectra measured on H_3_CLi/**1**H_2_ mixtures reproducibly showed resonances pointing toward a primary hydride-trapping product Li[**10**], which features a BHB bridge and two terminal hydrogen substituents in mutual *trans* arrangement (Scheme 6). For comparison, we prepared an authentic sample of Li[**10**] from **1**H_2_ and 1 equiv. of the ‘superhydride’ Li[HBEt_3_]. At low temperatures, Li[**10**] forms quantitatively; since the compound is thermolabile, its NMR spectra had to be recorded at –30 °C. Li[**10**] gives rise to a double set of proton resonances in THF solution. On average, the two 2,2′-biphenylylene fragments of the anion [**10**]^–^ should be related by a mirror plane containing the B_2_H_3_ core. The two phenylene rings of each individual 2,2′-biphenylylene moiety, however, are chemically inequivalent (as confirmed by 2D NMR experiments).

At room temperature, Li[**10**] readily isomerizes to the secondary hydride-trapping product Li[**7**], which we have isolated and characterized by NMR spectroscopy as well as X-ray crystallography. The anion of [Li(thf)_3_(Et_2_O)][**7**] consists of one 9-borafluorenyl and one BH_2_ fragment that are linked by a μ-H atom and a 2,2′-biphenylylene bridge (Scheme 5, bottom). As a result, both boron atoms are tetracoordinate and placed at a distance of B···B = 2.382(8) Å. In the solid state, the central seven-membered HB_2_C_4_ ring is non-planar and the anion possesses *C*_1_ symmetry (the torsion angle of the bridging 2,2′-biphenylylene amounts to 36°).

The molecular scaffolds of [**7**]^–^ and the known anion [**9**]^2–^ are essentially superimposable, apart from the fact that the latter features a covalent B–B bond (1.810(5) Å) instead of the μ-H atom (Scheme 5, bottom).^13^ In line with their marked structural resemblance, both anions exhibit similar ^1^H NMR spectra: in each case, three sets of aryl resonances are detectable. Two of those are well resolved at room temperature (H-a, H-b), whereas the third set consists of very broad signals, each of them integrating 2H (H-c; Scheme 6). This points toward a dynamic behavior of the compounds in solution, which likely arises from conformational changes of the twisted boron heterocycles. The ^11^B NMR spectrum of [**7**]^–^ is characterized by two resonances with chemical shift values of *δ* –3.6 and –10.1 ppm, testifying to the presence of two magnetically inequivalent, tetracoordinate boron nuclei.^38^

Turning our attention from the products of the reaction CH_3_Li/**1**H_2_ to its intermediates, we note that the ^1^H NMR spectrum of Li_2_[**4**] shows the same peculiarities as those of its structural congeners Li[**7**] and Li_2_[**9**]: well resolved resonances coexist with severely broadened signals. Together with a BCH_3_ resonance at *δ* –0.1 ppm, this can be taken as a support for our structural proposal of Li_2_[**4**], but the motional broadening precludes the measurement of meaningful ^13^C{^1^H} NMR and 2D correlation spectra. Despite numerous efforts, we have not succeeded in growing crystals of Li_2_[**4**] and therefore considered replacing the H_3_C group with an alternative sterically undemanding organic substituent: The reaction *t*BuCCLi/Li[**1**H] provided the alkynyl analogue Li_2_[**11**] of Li_2_[**4**] in single-crystalline form ([Li(12-crown-4)(thf)][Li(thf)_2_][**11**]; Scheme 6). X-ray crystallography confirmed the proposed ring-contracted, H-shifted structure of [**11**]^2–^.

NMR spectroscopy reproduced the characteristic distribution of well-resolved and motionally broadened line shapes; the chemical shift values of the aryl protons of Li_2_[**11**] are reasonably close to those of Li_2_[**4**] (*cf.* the ESI† for an overlay of the respective ^1^H NMR spectra). Remarkably, Li_2_[**11**] is also accessible *via* a different approach, starting from the doubly boron-doped dibenzo[*g*,*p*]chrysene Li_2_[**1**] and *t*BuCCH, the conjugate weak acid of [*t*BuCC]^–^ (Scheme 6).

**Fig. 3:**
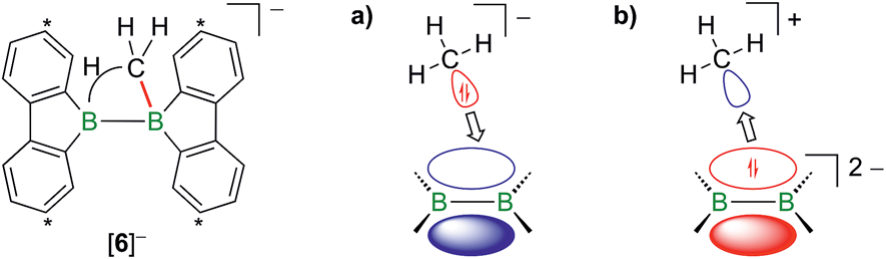
Two borderline cases to describe the bonding situation in [**6**]^–^ as (a) the [H_3_C]^–^ adduct of a diborane(4) and (b) the [H_3_C]^+^ adduct of a [**1**]^2–^ anion. Carbon atoms marked with asterisks bear *t*Bu substituents.

The facile protonation^14^ of Li_2_[**1**] prompted us to investigate whether an umpolung approach to synthesize compounds of the type Li[**2**] might also be successful, which would provide fundamentally interesting insights into the reactivities of B=B double-bonded species. As mentioned above, the intermediate Li[**6**] of the reaction H_3_CLi/**1**H_2_ can be regarded as the [H_3_C]^–^ adduct of a diborane(4). Conceptually, it should be possible to arrive at the same molecule by formally transferring two electrons from the carbon nucleophile to the redox-active organoborane and thus starting from methylium-ion sources and the anion [**1**]^2–^ (Fig. 3).^39^

**Scheme 7:**
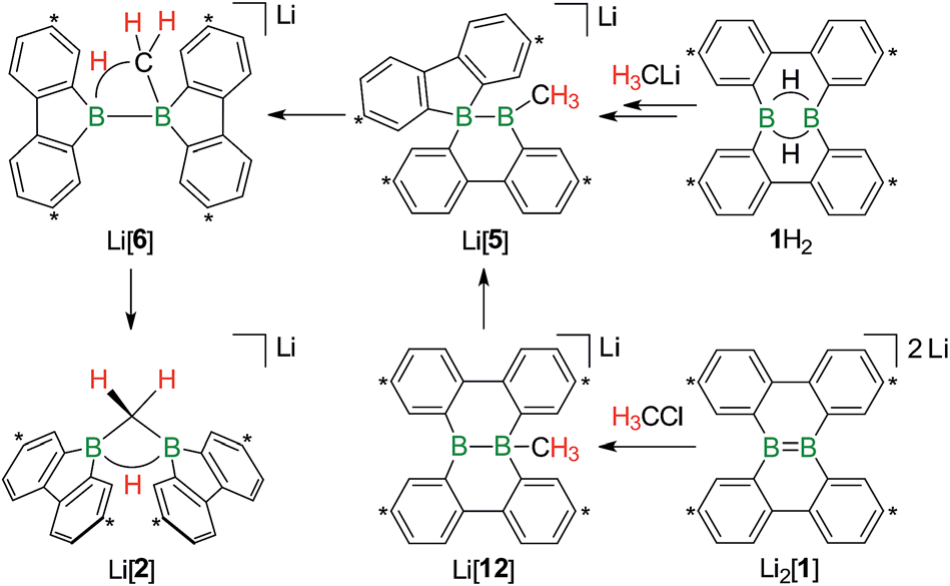
The addition of H_3_CCl to Li_2_[**1**] quantitatively furnishes Li[**2**]. The reaction pathways to Li[**2**], starting from either **1**H_2_ or Li_2_[**1**], merge at the stage of Li[**5**] (*cf.* also Scheme 5). Carbon atoms marked with asterisks bear *t*Bu substituents.

Indeed, when a THF solution of Li_2_[**1**] is stirred at room temperature under a blanket of H_3_CCl gas (1 atm), a quantitative conversion to Li[**2**] occurs (Scheme 7).^40^ This approach is far more atom- and time-economic than the previous access route *via* the polarity-inverted couple H_3_CLi/**1**H_2_, because we avoid wasting 50% of **1**H_2_ as a hydride-trapping reagent and do no longer have to separate the resulting hydride-trapping products. Mechanistically, the electron-rich B=B fragment of Li_2_[**1**] likely acts as a nucleophile toward H_3_CCl to form [**12**]^–^, which carries a boron-bonded methyl substituent and contains a central B–B single bond. The B(sp^2^)–B(sp^3^) species Li[**12**] then undergoes a 1,2-phenyl shift to afford Li[**5**] and thereby funnels into the reaction cascade outlined above for the formation of Li[**2**] from H_3_CLi/**1**H_2_ (Scheme 7).

**Scheme 8:**
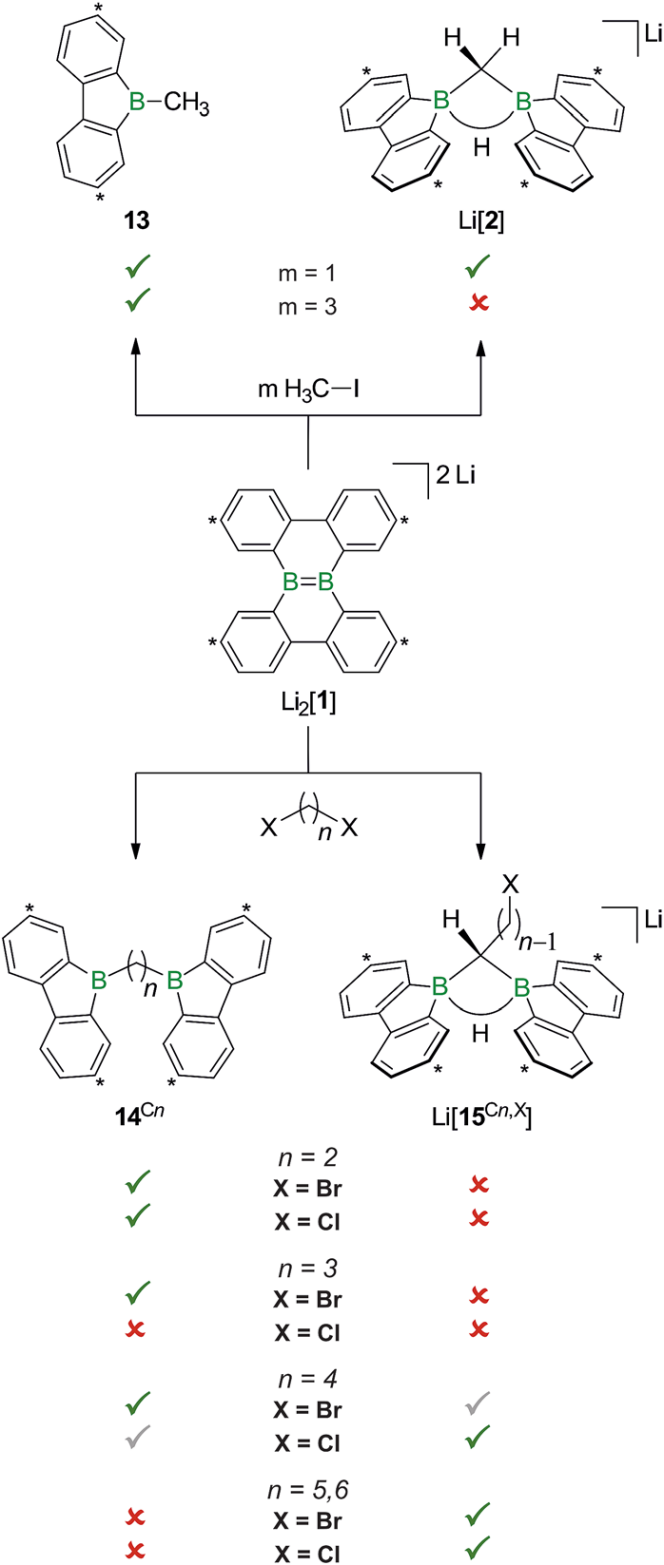
The outcome of the reaction H_3_C–I/Li_2_[**1**] depends on the stoichiometries employed. While 1 : 1 mixtures give **13** together with Li[**2**], 3 : 1 mixtures exclusively furnish **13**. Use of α,ω-dihaloalkanes X(CH_2_)_*n*_X instead of H_3_C–I affords ditopic boranes **14**^C*n*^ (*n* = 2–4) and/or Li[**15**^C*n*,X^] (*n* = 4–6; X = Cl, Br). Carbon atoms marked with asterisks bear *t*Bu substituents.

**Scheme 9:**
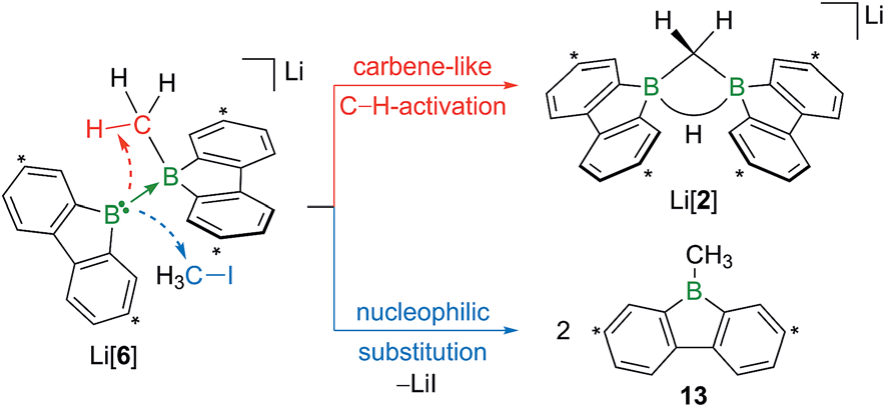
The intermediate Li[**6**] can be interpreted as an adduct between the 9-borafluorenyl anion ([BFlu]^–^) and 9-methyl-9-borafluorene (**13**). Intramolecular C–H insertion of the carbene-like [BFlu]^–^ furnishes Li[**2**]; intermolecular nucleophilic attack on H_3_C–I affords 2 equiv. of **13**. Carbon atoms marked with asterisks bear *t*Bu substituents.

When H_3_CCl is replaced by 1 equiv. of iodomethane (H_3_C–I), the outcome is a mixture of Li[**2**], 9-methyl-9-borafluorene (**13**), and residual Li_2_[**1**] (Scheme 8). After increasing the relative amount of H_3_C–I to 3 equiv., we almost exclusively obtained **13**. The different behaviors of the two halomethanes can be rationalized by viewing the intermediate Li[**6**] as an adduct between the 9-borafluorenyl anion ([BFlu]^–^) and (H_3_C)BFlu (**13**; Scheme 9). [BFlu]^–^ is isoelectronic to the carbene 9-fluorenylidene. A formal carbene-like reactivity is reflected by the intramolecular insertion of [BFlu]^–^ into the C–H bond of the 9-methyl-9-borafluorene moiety to afford Li[**2**]. When the strong electrophile H_3_C–I with its excellent iodide leaving group is present, also the nucleophilic character of [BFlu]^–^ comes into play and opens a competing intermolecular pathway, which ultimately leads to **13**. As the relative amount of H_3_C–I is increased, the substitution reaction becomes dominant (we note in passing that the reaction with H_3_C–I can alternatively be viewed as a carbene-like insertion of [BFlu]^–^ into the C–I bond with subsequent elimination of LiI).

**Fig. 4:**
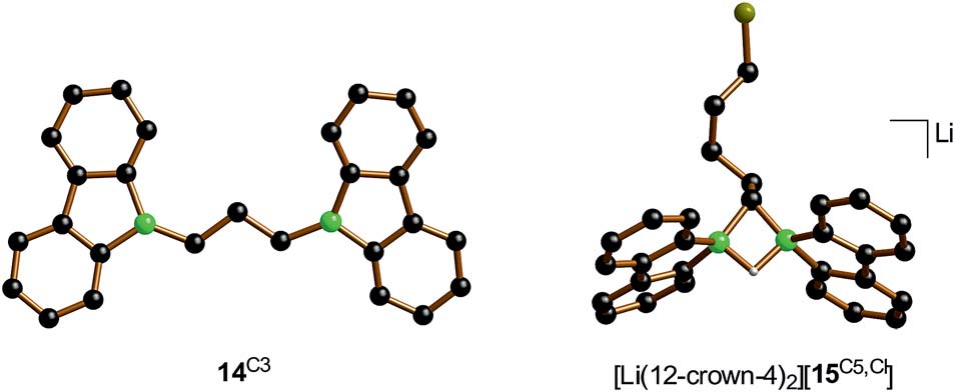
Molecular structures of **14**^C3^ and of the terminally chlorine-substituted [Li(12-crown-4)_2_][**15**^C5,Cl^] in the solid state. The solvent-separated [Li(12-crown-4)_2_]^+^ cation, all *t*Bu groups, and all CH atoms are omitted for clarity.

In case of the system H_3_C–I/Li_2_[**1**], the methyl group initially gets attached to only one of the symmetry-related boron centers, but the other is equally important for the subsequent C–H-activation and nucleophilic substitution steps. The degree of B–B cooperativity in Li_2_[**1**] as well as the insertion *vs.* nucleophilic behavior of [BFlu]^–^ thus deserve a detailed assessment. To this end, we conducted a systematic study using 1 : 1 mixtures of Li_2_[**1**] and α,ω-dihaloalkanes X(CH_2_)_*n*_X with chain lengths in the range of *n* = 2–6 and leaving groups of different qualities (*e.g.*, X = Cl, Br). In these experiments, smaller alkylidene linkers are supposed to mimic higher local concentrations of the electrophile. As summarized in Scheme 8, clean twofold substitution reactions are observed with the short-chain substrates (*n* = 2 and 3, *cf.***14**^C2^ and **14**^C3^; 1,3-dichloropropane leads to a complex mixture of products). Clean C–H-activation reactions occur with the long-chain substrates (*n* = 5 and 6) to afford the haloalkyl species Li[**15**^C5,Cl^]/Li[**15**^C5,Br^] and Li[**15**^C6,Cl^]/Li[**15**^C6,Br^]. The medium-chain substrates (*n* = 4) mark the switching point between both scenarios: with the worse chloride leaving group, C–H-activation is preferred over the twofold substitution. The reverse is true in the case of the better bromide leaving group. The solid-state structures of **14**^C2^·thf, **14**^C3^ (Fig. 4), **14**^C4^, and [Li(12-crown-4)_2_][**15**^C5,Cl^] (Fig. 4) were characterized by X-ray crystallography (*cf.* the ESI† for full information). Also the connectivities of [Li(thf)_4_][**15**^C4,Cl^], [Li(thf)_4_][**15**^C6,Cl^], and [Li(thf)_4_][**15**^C6,Br^] are supported by X-ray diffraction studies, however, due to disordered haloalkyl chains, *t*Bu groups, and THF molecules, the quality of these three structures prevents their inclusion into this publication.^41^

The observed chain-length dependence of the product distribution suggests that the carbene-type insertion and the second nucleophilic substitution both follow an intramolecular pathway involving two cooperating boron atoms.

If the remaining CH_2_X center and the BCH_2_ group are similarly close to the B–B bond, the nucleophilic process occurs at a higher rate than the carbene-type C–H-activation. As the alkylidene spacer grows, the second electrophilic functionality moves further apart whereas the reactive α-CH_2_ unit stays in place such that the C–H-activation becomes more and more relevant until it finally takes over.

**Scheme 10:**
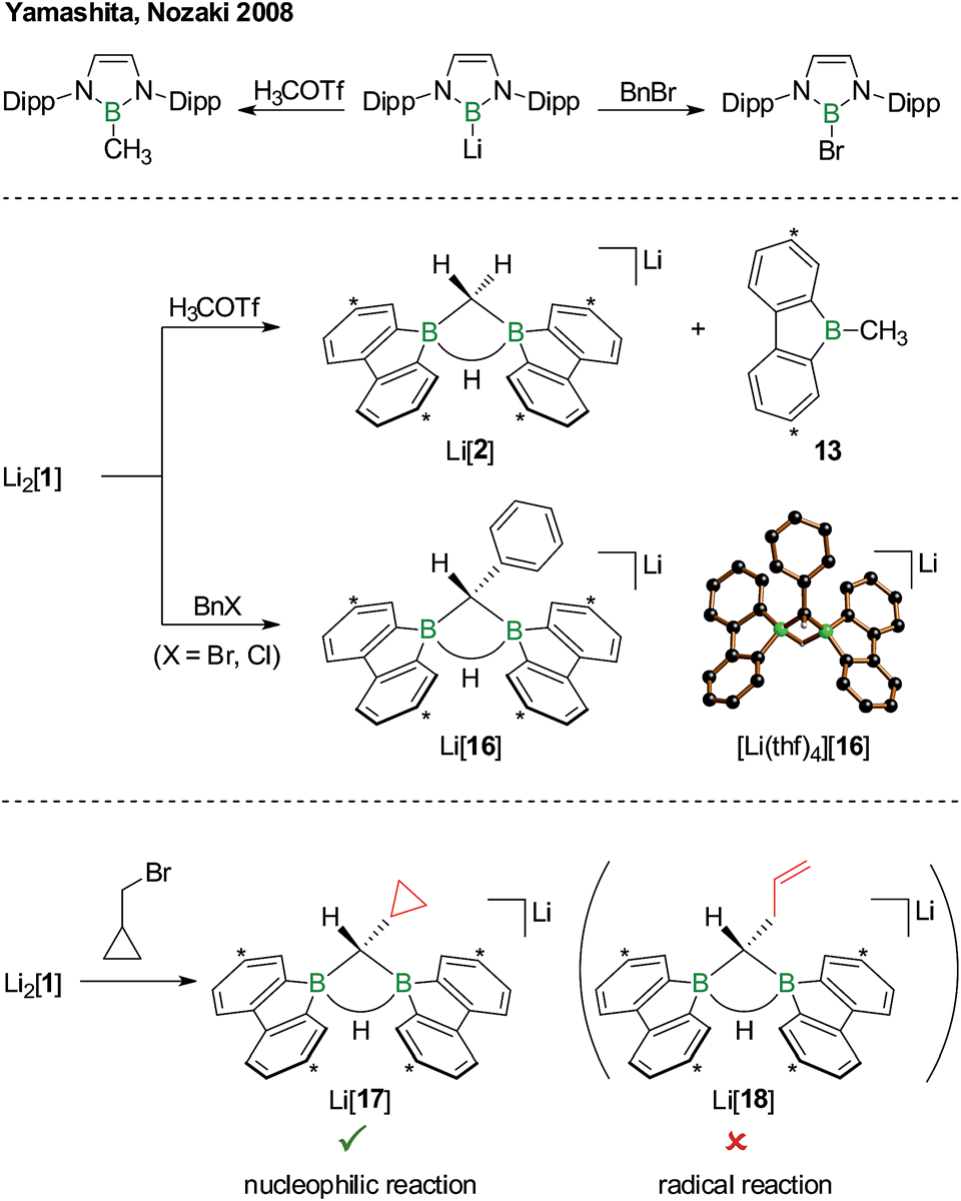
The reactions of Yamashita's and Nozaki's boryllithium compound with H_3_COTf or BnBr furnish the corresponding methyl borane or bromoborane, respectively (top). In the analogous reactions with Li_2_[**1**], only the organyl moieties are transferred to boron (middle; *cf.* Li[**2**]/**13** and Li[**16**]). The reaction of Li_2_[**1**] with the radical clock (bromomethyl)cyclopropane quantitatively furnishes Li[**17**], which is a strong indication for a closed-shell, nucleophilic pathway (bottom). Dipp = 2,6-(*i*Pr)_2_C_6_H_3_, Bn = CH_2_C_6_H_5_, H_3_COTf = H_3_COSO_2_CF_3_; carbon atoms marked with asterisks bear *t*Bu substituents. In the crystal structure plot of [Li(thf)_4_][**16**], the solvent-separated cation, the *t*Bu groups, and all C(sp^2^)–H atoms are omitted for clarity.

Although the reaction between Li_2_[**1**] and, *e.g.*, H_3_C–I can convincingly be rationalized by assuming a nucleophilic pathway, the possible operation of a radical mechanism remains to be ruled out. We first note in this context that 1,2-dihaloethane in the presence of Li_2_[**1**] did not undergo reductive dehalogenation with ethene formation. Yamashita, Nozaki *et al.* have treated their boryllithium compound with methyl trifluoromethanesulfonate (H_3_COTf)^42^ on the one hand and benzyl bromide (BnBr) on the other (Scheme 10, top). In the first case, they observed the corresponding methyl borane in yields of 85%, whereas in the second case exclusively the bromoborane was obtained.^43^ To explain the different outcomes, they proposed halogenophilic attack of the boryllithium or single electron transfer to the benzyl halide. We repeated Nozaki's experiments with Li_2_[**1**] (Scheme 10, middle): H_3_COTf showed the same reactivity as described above for H_3_C–I (*cf.* Li[**2**] and **13**); BnBr (as well as BnCl) gave the C–H-activation product Li[**16**] rather than any haloboranes, as confirmed by NMR spectroscopy and X-ray crystallography on [Li(thf)_4_][**16**].

As the ultimate test, we added Li_2_[**1**] to 1 equiv. of (bromomethyl)cyclopropane, a well-established radical clock (Scheme 10, bottom).^44^–^46^ A quantitative conversion to the C–H-activation product Li[**17**], still carrying an intact cyclopropyl substituent, occurred (NMR-spectroscopic control). The absence of the ring-opened olefin derivative Li[**18**] in the reaction mixture strongly supports the proposal of a closed-shell scenario in contrast to an open-shell process.

The results collected thus far are not only fundamentally interesting with respect to the reactivities of electron-rich B=B double bonds, but open new access routes to ditopic boranes of high Lewis acidity. Molecules containing two or more potentially cooperating boron sites are of great current interest, *inter alia*, as organocatalysts^5^,^11^,^47^ or electron-storage media.^48^,^49^ Compounds of the class **14**^C*n*^ already constitute free Lewis acids, but do not contain functional groups amenable to further derivatization.

The opposite is true for the salts Li[**15**^C*n*,X^]. Here, the terminal halogen atoms provide ample opportunities, *e.g.*, for grafting the organoboron units onto polymers, dendrimers, or surfaces, but the Lewis acids need to be activated through LiH elimination prior to use.

**Scheme 11:**
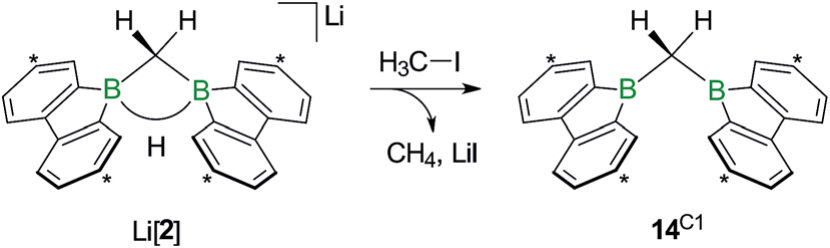
The addition of H_3_C–I to Li[**2**] furnishes the bis(9-borafluorenyl)methane **14**^C1^. Carbon atoms marked with asterisks bear *t*Bu substituents.

While the bulky hydride scavenger (H_3_C)_3_SiCl failed in this respect, the smaller electrophile H_3_C–I efficiently transformed the model compound Li[**2**] to its conjugate acid **14**^C1^ (Scheme 11). As important diagnostic criteria, the BHB proton resonance vanishes in the course of the reaction, and the ^11^B NMR signal shifts from the tetracoordinate (Li[**2**]: *δ* –14 ppm) to the tricoordinate spectral region (**14**^C1^: *δ* 45 ppm).^49^,^50^

In line with the reaction H_3_C–I/Li[**2**], the haloalkyl derivatives Li[**15**^C*n*,X^] are not long-term stable in THF at room temperature: ^1^H NMR monitoring of the solutions revealed in each case a gradual decrease of the CH_2_X resonance and a concomitant increase of a signal assignable to a terminal CH_3_ group, which leads to the conclusion that the pending haloalkyl substituent can take a similar role as added H_3_C–I. It is important to note in this context that the follow-up X/H exchange reactions are completely suppressed at –78 °C and even at room temperature slow enough not to interfere with targeted derivatizations of the CH_2_X termini.

## Conclusion

In summary, C(sp^3^)–H activation and nucleophilic substitution reactions have been performed on the same redox-active diborane platform. We propose that the doubly 2,2′-biphenylylene-bridged diborane(6) **1**H_2_ reacts with H_3_CLi to furnish the rearranged B(sp^2^)–B(sp^3^) intermediate Li[FluB–BFlu(CH_3_)] (Li[**6**]; BFlu = 9-borafluorenyl). Li[**6**] also forms *via* an umpolung approach starting from H_3_CX and the B=B bonded, nucleophilic Li_2_[**1**], a compound which can be regarded as the product of a double deprotonation of **1**H_2_ (X = Cl, I). Li[**6**] readily undergoes B–B-bond heterolysis to formally give the [BFlu]^–^ anion and (H_3_C)BFlu (**13**). The final product distribution depends on the relative amount of H_3_CX and the leaving-group qualities of X, because [BFlu]^–^ can either insert into a C(sp^3^)–H bond of **13** or replace the halogen atom of a second equivalent of H_3_CX. The product of the carbene-type C–H insertion is Li[FluB(μ-CH_2_)(μ-H)BFlu] (Li[**2**]) while the nucleophilic substitution on C–X generates 2 equiv. of **13**. Further insight into the competition between the two scenarios was gained with the help of α,ω-dihaloalkanes X(CH_2_)_*n*_X (X = Cl, Br). In the resulting intermediates Li[FluB–BFlu((CH_2_)_*n*_X)], both possible follow-up reactions should be intramolecular processes. A longer alkylidene chain corresponds to a lower local concentration of the electrophile, while the BCH_2_ groups are always similarly close to the reactive B–B bond. Consequently, short chains (*n* = 2,3) result in double substitution products FluB(CH_2_)_*n*_BFlu and long chains (*n* = 5,6) in C–H-activation products Li[FluB(μ-C(H)(CH_2_)_*n*–1_X)(μ-H)BFlu]. In the case of the intermediate chain length *n* = 4, a mixture of both compounds is obtained: the worse leaving group X = Cl leads to a higher proportion of the C–H-activated species, the better leaving group X = Br furnishes more FluB(CH_2_)_4_BFlu. We finally note that the B–B-bond heterolysis of Li[**6**] with concomitant transfer of a reactive [BFlu]^–^ moiety is reminiscent of the reactivity patterns of the widely used alkoxy-diborane(4) adducts [pinB–Bpin(OR)]^–^.^25^ As a decisive difference, however, [BFlu]^–^ appears to be considerably more reactive than *in situ*-generated [Bpin]^–^, because C–H-insertion reactions of the latter are so far unknown.

## Conflicts of interest

There are no conflicts to declare.

## Supplementary Material

Supplementary information

Crystal structure data
