# Supplementary material for: A redox-active diborane platform performs C(sp3)–H activation and nucleophilic substitution reactions
Source: Chem Sci. 2018 Mar 19;9(15):3881–91. doi: 10.1039/c8sc00743h (PMC5942040; doi:10.1039/c8sc00743h)
Supplement: Supplementary file 1 [file SC-009-C8SC00743H-s001.pdf]

Electronic Supplementary Material (ESI) for Chemical Science.
This journal is © The Royal Society of Chemistry 2018

Electronic Supplementary Information

A Redox-Active Diborane Platform Performs C(sp^3)–H Activation and Nucleophilic Substitution Reactions

Thomas Kaese, Timo Trageser, Hendrik Budy, Michael Bolte, Hans-Wolfram Lerner and Matthias Wagner*

Content:

1. Experimental details and characterization data ESI2-ESI22
2. Plots of NMR spectra ESI23-ESI46
3. X-ray ESI47-ESI59
4. Computational details ESI60-ESI69
5. References ESI70

1. Experimental details and characterization data

General considerations. All reactions and manipulations were carried out in an argon-filled glovebox or by applying standard Schlenk techniques under an argon atmosphere. Hexane was dried over Na, THF and Et_2O were dried over Na/benzophenone, THF-d_8 was dried over Na-K alloy. Prior to use, the solvents were distilled from the drying agent, degassed by applying three freeze-pump-thaw cycles, and stored over activated molecular sieves (3 Å). Compounds **1**H_2,[S1] Li[**1**H],[S2] and Li_2[**1**][S3] were synthesized according to literature procedures. The Et_2O solution of the $D_3CLi{\cdot}LiI$ complex (Sigma Aldrich) is commercially available with 99 atom-% deuterium and was used as received. Commercial Et_3SiD (Santa Cruz Biotechnology) contained 98 atom-% deuterium. Prior to use, the liquid haloalkanes were degassed by applying three freeze-pump-thaw cycles. NMR: Bruker DPX 250, Avance 300, Avance III 500 HD. Chemical shifts are referenced to (residual) solvent signals ($^{1}H/^{13}C\{^{1}H\}$; THF-d_8: δ = 3.58/67.21 ppm),[S4] external LiCl in D_2O (^{7}Li), or external $BF_3{\cdot}Et_2O$ (^{11}B; $^{11}B\{^{1}H\}$). Abbreviations: s = singlet, d = doublet, t = triplet, q = quartet, m = multiplet, br = broad, n.r. = multiplet expected in the NMR spectrum but not resolved, n.o. = not observed, vq = virtual quartet, vquint = virtual quintet.

Reaction of $1H_2$ with 1 equiv. of H_3CLi. An Et_2O solution of H_3CLi (0.5 M, 0.05 mL, 25 µmol) was evaporated to dryness in an NMR tube and the colorless solid residue was dissolved in THF-d_8 (0.6 mL) at room temperature. The solution was frozen at –196 °C and colorless **1**H_2 (14 mg, 25 µmol) was added. The NMR tube was evacuated, flame sealed, and warmed to room temperature. The ^{1}H NMR spectroscopic investigation of the orange reaction solution revealed Li[**1**H],[S2] Li[**2**], and Li[**7**] as the major products (each ca. 20-40%; the product distribution varied to some extent between repeated experiments).

In a glovebox, a representative NMR sample was transferred to an uncapped glass vial, and the vial was placed in a larger glass vessel containing hexane (2 mL). The outer vessel was covered with a lid to allow for gas-phase diffusion of the solvents in a closed environment. After 2-3 d, three types of single crystals (1 x yellow, 2 x colorless) had grown. According to X-ray crystallography, the yellow crystals consisted of the known salt [Li(thf)$_3$][**1**H].[S2] Of the colorless specimen, the first kind were too poorly diffracting to perform a full refinement, but, according to ^{1}H NMR spectroscopy, they consisted of Li[**7**] (cf. the reaction of **1**H_2 with Li[$HBEt_3$] for a targeted synthesis and the characterization of Li[**7**]). The second kind of colorless crystals turned out to be [Li(thf)$_4$][**2**] (for the X-ray crystal structure of the differently solvated analog [Li(thf)$_3$][**2**], see the reaction of Li_2[**1**] with methyl triflate).

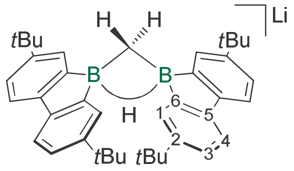

Figure S1. NMR numbering scheme for Li[**2**].

*Li[**2**]*

^{1}H NMR (500.2 MHz, THF-d_8): δ = 7.99 (d, ^4J(H,H) = 1.9 Hz, 4H; H-1), 7.41 (d, ^3J(H,H) = 7.9 Hz, 4H; H-4), 7.06 (dd, ^3J(H,H) = 7.9 Hz, ^4J(H,H) = 1.9 Hz, 4H; H-3), 1.94 (br, 1H; μ-H), 1.42 (s, 36H; CH_3), 0.49 (d, ^3J(H,H) = 4.8 Hz, 2H; CH_2).

^{7}Li NMR (194.4 MHz, THF-d_8): δ = –0.3.

^{11}B NMR (160.5 MHz, THF-d_8): δ = –14.0 (br).

^{13}C{^{1}H} NMR (125.8 MHz, THF-d_8): δ = 159.1 (C-6), 147.8 (C-5), 146.3 (C-2), 127.9 (C-1), 121.8 (C-3), 117.7 (C-4), 35.2 (CCH_3), 32.6 (CH_3), 5.1 (CH_2).

Reaction of Li[2] with H_3C–I. An NMR tube was charged at room temperature with [Li(thf)$_4$][**2**] (5.0 mg, 5.8 µmol), THF-d_8 (0.5 mL), and H_3C–I (1.0 µl, 2.3 mg, 16 µmol). According to ^{1}H, ^{11}B, and ^{13}C NMR spectroscopic investigations, Li[**2**] had vanished after 3 d and **14**^{C1} formed as the major product besides trace amounts of **13**.

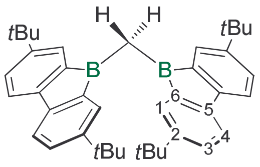

Figure S2. NMR numbering scheme for **14**^{C1}.

14*^{C1}*

^{1}H NMR (500.2 MHz, THF-*d*$_8$): δ = 7.41 (d, ^{4}J(H,H) = 1.9 Hz, 4H; H-1), 7.13 (d, ^{3}J(H,H) = 7.8 Hz, 4H; H-4), 7.07 (dd, ^{3}J(H,H) = 7.8 Hz, ^{4}J(H,H) = 1.9 Hz, 4H; H-3), 1.93 (s, 2H; CH_2), 1.18 (s, 36H; CH_3).
^{11}B NMR (160.5 MHz, THF-*d*$_8$): δ = 45 (vbr).
^{13}C{^{1}H} NMR (125.8 MHz, THF-*d*$_8$): δ = 149.3 (C-5), 149.2 (C-2), 147.8 (C-6), 129.7 (C-1), 127.2 (C-3), 118.4 (C-4), 34.9 (*C*CH_3), 31.7 (CH_3), 14.8 (CH_2).
HRMS: Calculated for $C_{41}H_{50}B_2$: 564.40931, found: 564.41115.

Reaction of $1H_2$ with 1 equiv. Li[$HBEt_3$].

At –30°C

In an NMR tube, a THF solution of Li[$HBEt_3$] (1 M, 38 µL, 38 µmol) was evaporated to dryness at room temperature in a dynamic vacuum. During the addition of a THF-d_8 solution of **1**H_2 (0.5 mL, 21 mg, 38 µmmol) and the subsequent flame-sealing of the NMR tube, the lower part of the tube was cooled to –196 °C. The sample was stored at –70 °C for 3 h prior to its NMR spectroscopic investigation at –30 °C, which showed a selective transformation to the primary hydride trapping product Li[**10**].

At room temperature

When carried out in neat THF (6 mL) at room temperature, the reaction between **1**H_2 (79 mg, 143 µmol) and Li[$HBEt_3$] (0.05 M in THF, 2.7 mL, 135 µmol) furnished a clear, pale yellow solution. A ^{1}H NMR spectroscopic investigation (THF-d_8) showed the quantitative consumption of **1**H_2 and the formation of two new major compounds (*i.e.*, the primary hydride trapping product Li[**10**] and its isomer Li[**7**]). After heating the sample at 50 °C for 1 h, Li[**7**] became by far the major constituent of the product mixture (>70%).
To obtain crystals, a solution of Li[$HBEt_3$] (0.2 M, 0.36 mL, 72 µmol) in THF/Et_2O (1:4) was added at room temperature to a colorless suspension of **1**H_2 (40 mg, 72 µmol) in Et_2O (3 mL). After stirring for 10 min, the reaction mixture was filtered through a PTFE syringe filter (0.2 µm). Colorless thin needles of [Li(thf)$_3$(Et_2O)][**7**] grew from the filtrate at room temperature within 1 d.

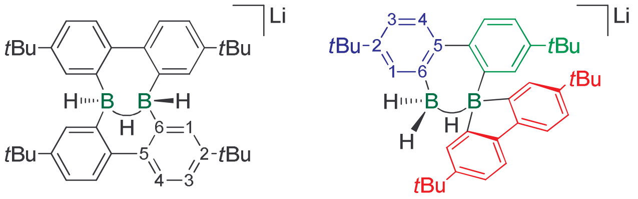

Figure S3. NMR numbering scheme for the primary hydride trapping product Li[**10**] (left) and its isomer Li[**7**] (right).

*Li[**10**]*

^{1}H NMR (500.2 MHz, THF-d_8): δ = 7.61 (d, ^{4}J(H,H) = 1.9 Hz, 2H; H-1), 7.27 (d, ^{3}J(H,H) = 8.0 Hz, 2H; H-4), 7.11 (dd, ^{3}J(H,H) = 8.0 Hz, ^{4}J(H,H) = 1.9 Hz, 2H; H-3), 6.94 (d, ^{3}J(H,H) = 8.0 Hz, 2H; H-4), 6.78 (d, ^{4}J(H,H) = 1.9 Hz, 2H; H-1), 6.59 (dd, ^{3}J(H,H) = 8.0 Hz, ^{4}J(H,H) = 1.9 Hz, 2H; H-3), 1.38 (s, 18H; CH_3), 0.94 (s, 18H; CH_3).
^{7}Li NMR (194.4 MHz, THF-d_8): δ = –0.6.
^{11}B NMR (96.3 MHz, THF-d_8): δ = 33.5 (br), 8.2 (br).
^{13}C{^{1}H} NMR (125.8 MHz, THF-d_8): δ = 149.6 (C-6), 149.2 (C-6), 145.4 (C-2), 144.8 (C-5), 144.5 (C-2), 141.5 (C-5), 134.4 (C-1), 129.6 (C-1), 125.7 (C-4), 125.4 (C-4), 122.1 (C-3), 119.9 (C-3), 34.6 (CCH_3), 34.2 (CCH_3), 32.1 (CH_3), 31.6 (CH_3).
Resonances of the same color belong to the same phenyl ring (as confirmed by 2D NMR experiments).

*Li[**7**]*

^{1}H NMR (500.2 MHz, THF-*d*$_8$): δ = 7.48 (d, ^4J(H,H) = 2.3 Hz, 1H; H-1), 7.39 (d, ^4J(H,H) = 2.3 Hz, 1H; H-1), 7.38 (d, ^3J(H,H) = 8.0 Hz, 1H; H-4), 7.31 (d, ^3J(H,H) = 7.9 Hz, 2H; H-4), 7.29 (d, ^3J(H,H) = 8.0 Hz, 1H; H-4), 7.23 (dd, ^3J(H,H) = 8.0 Hz, ^4J(H,H) = 2.3 Hz, 1H; H-3), 7.05 (dd, ^3J(H,H) = 8.0 Hz, ^4J(H,H) = 2.3 Hz, 1H; H-3), 6.97 (n.r., 2H; H-3), 2.6 (vbr, 1H; BH), 1.34 (s, 9H; CH_3), 1.18 (s, 9H; CH_3), 1.18 (br, 18H; CH_3), n.o. (H-1 (2H), BH (2H)).

^{7}Li NMR (194.4 MHz, THF-*d*$_8$): δ = –0.7.

^{11}B NMR (96.3 MHz, THF-*d*$_8$): δ = –3.6 (br), –10.1 (br).

^{13}C{^{1}H} NMR (125.8 MHz, THF-*d*$_8$): δ = 147.4 (br), 147.0, 146.5, 145.9, 145.6, 145.5, 133.9 (C-1), 130.2 (C-1), 129.6 (C-1), 126.4 (C-4), 125.9 (C-4), 122.9 (C-3), 122.3 (C-3), 121.8 (C-3), 117.5 (C-4), 34.9 (*C*CH_3), 34.8 (*C*CH_3), 34.6 (*C*CH_3), 32.2 (CH_3), 32.1 (CH_3), 32.0 (CH_3), n.o. (3 x C^{Ar}).

Resonances of the same color belong to the same phenyl ring (as confirmed by 2D NMR experiments). Signals marked in red could be unequivocally assigned, because they possess double intensity; the blue/green resonances were tentatively assigned by comparison of their chemical shift values with those of a related compound featuring a B–B bond instead of the bridging H atom.[S5]

Reaction of 1H_2 with exc. LiH. In an NMR tube, THF-*d*$_8$ (0.6 mL) was added at room temperature to a solid mixture of **1**H_2 (7 mg, 13 µmol) and LiH (15 mg, 1.9 mmol). ^{1}H and ^{11}B NMR spectra recorded on this mixture after 10 h showed no reaction. However, upon heating (60 °C, 1 d) the lithium dihydrido-2,7-di(*t*Bu)-9-boratafluorene[S3] evolved as the major product (ca. 80%); further heating at 100 °C for 1d resulted in the quantitative conversion of **1**H_2 to the lithium dihydrido-2,7-di(*t*Bu)-9-boratafluorene.

Deuterium-labeling experiments

*(A) Synthesis of **1**D_2*

Compound **1**D_2 was obtained according to the published synthesis of **1**H_2,[S1] but by using Et_3SiD instead of Et_3SiH (Scheme S1).

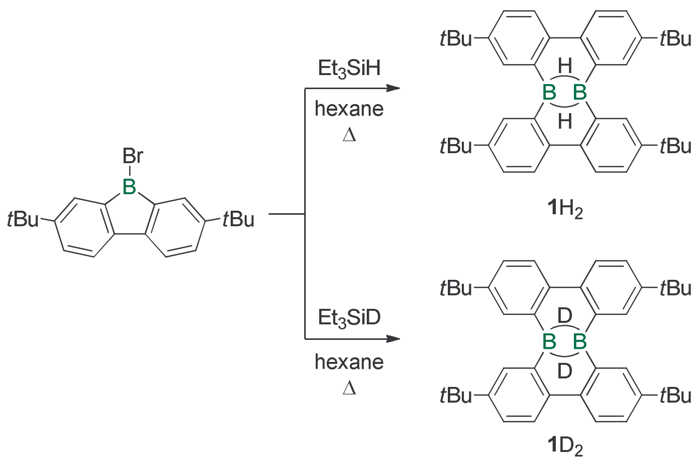

Scheme S1. Reaction of 2,7-di(*t*Bu)-9-Br-9-borafluorene with Et_3SiH or Et_3SiD to afford **1**H_2 or **1**D_2, respectively.

***1**D_2*

The ^{1}H, $^{11}B\{^{1}H\}$, and $^{13}C\{^{1}H\}$ NMR spectra (C_6D_6) of **1**H_2[S1] and **1**D_2 are identical with the exception of a broad proton resonance at 3.5 ppm, which has been assigned to the bridging H-atoms in **1**H_2[S1] and which is missing in **1**D_2. Instead, **1**D_2 shows a signal at 3.4 ppm in the ^{2}H NMR spectrum.

*(B) Reaction of **1**H_2 with D_3CLi*

A solution of D_3CLi·LiI in Et_2O (0.5 M, 0.05 mL, 25 µmol) was evaporated to dryness in an NMR tube. The resulting colorless residue was dissolved in THF-d_8 and the solution was frozen at –196 °C. Colorless **1**H_2 (14 mg, 25 µmol) was added, the NMR tube was vacuum sealed, and warmed to room temperature. The orange reaction mixture was analyzed by NMR spectroscopy (see section *D*).

*(C) Reaction of **1**D_2 with H_3CLi*

A solution of H_3CLi in Et_2O (0.25 M, 0.14 mL, 35 µmol) was evaporated to dryness in an NMR tube. The resulting colorless residue was dissolved in THF-d_8 and the solution was frozen at –196 °C. Colorless **1**D_2 (20 mg, 36 µmol) was added, the NMR tube was vacuum sealed, and warmed to room temperature. The orange reaction mixture was analyzed by NMR spectroscopy (see section *D*).

(D) Results of the deuterium-labeling experiments

The reactions D_3CLi/**1**H_2 and H_3CLi/**1**D_2 in THF-d_8 give complex product mixtures in which Li[**1**H]/Li[**1**D] and Li[**2**]/Li[**2**-d_3] are present as major constituents (Figure S4). The additional, poorly resolved signals in the range 7.7 ppm to 6.5 ppm are due to hydride-trapping products. The product distributions critically depend on the exact stoichiometries employed.

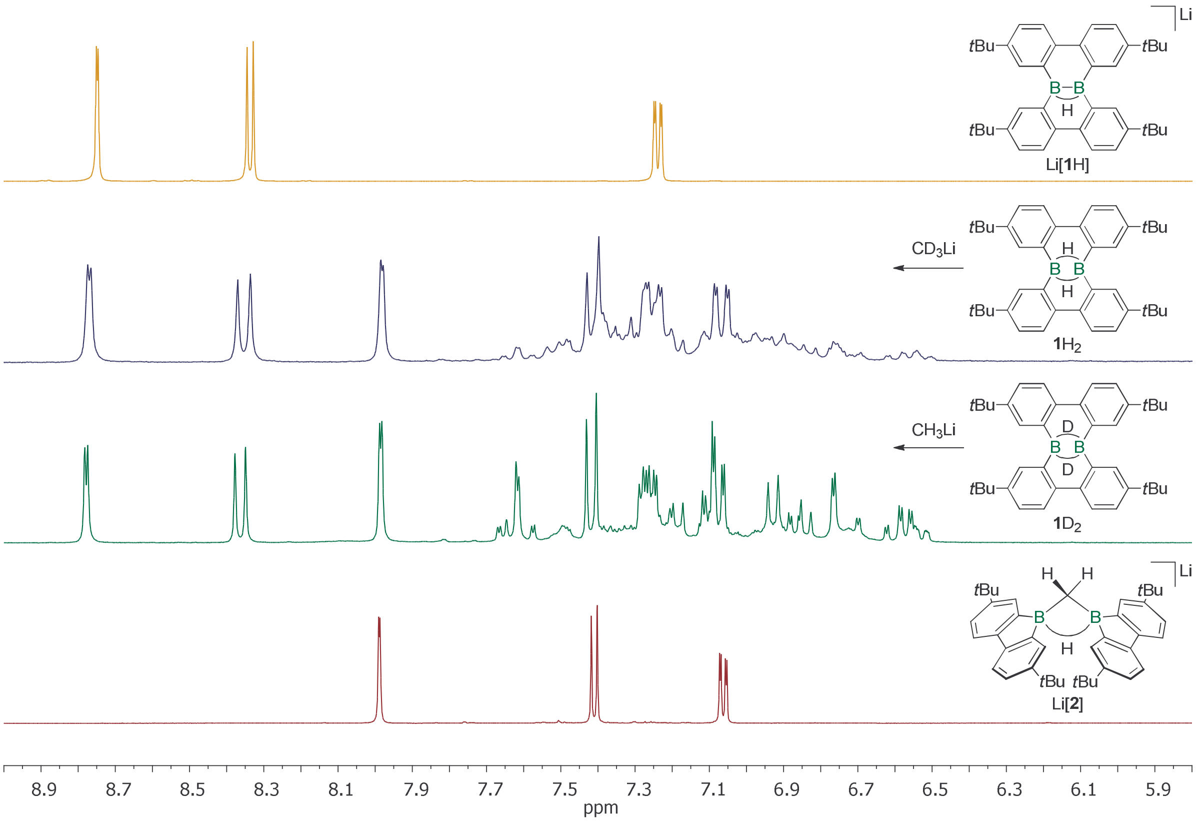

Figure S4. Aromatic regions of ^1H NMR spectra (THF-d_8) recorded on the reaction mixtures of D_3CLi/**1**H_2 (blue; 250.1 MHz) and H_3CLi/**1**D_2 (green; 300.0 MHz). Matching spectra of Li[**1**H] (orange; 500.2 MHz) and Li[**2**] (red; 500.2 MHz) are shown for comparison. *Note*: The spectra have been recorded at different spectrometer frequencies, which leads to slight differences in the line shapes of corresponding signals.

Li[**2**] shows two characteristic ^{1}H NMR signals, which are particularly relevant for the mechanistic considerations: a broad resonance at 1.94 ppm (μ-H) and a doublet at 0.49 ppm (CH_2). Both signals are observed in the ^{1}H NMR spectra of H_3CLi/**1**D_2, but absent in D_3CLi/**1**H_2 (Figure S5). Thus, the μ-H atom of Li[**2**] does not originate from the boron-bonded H atoms of the diborane(6) starting material (**1**H_2). Rather, all the CH_2 and μ-H atoms originate from the methyllithium reagent.

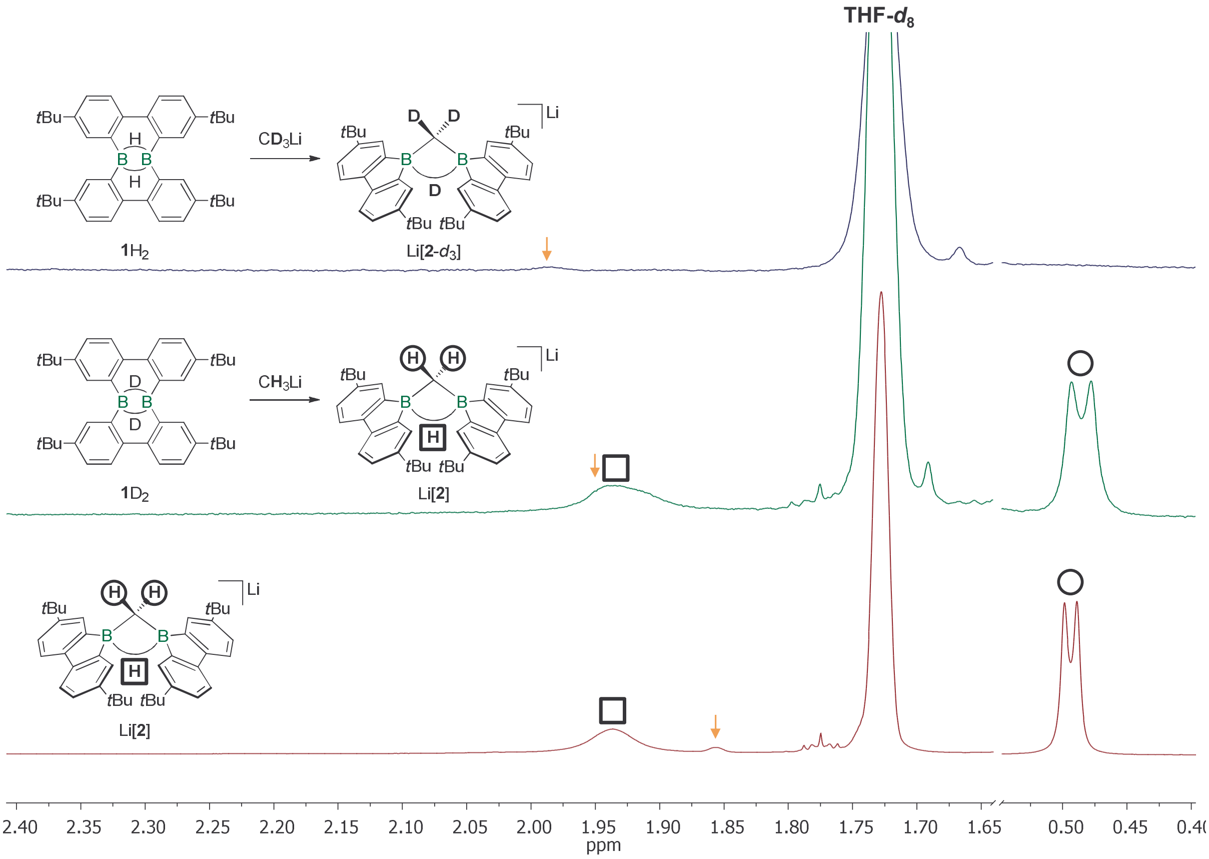

Figure S5. ^{1}H NMR spectra (THF-d_8) recorded on the reaction solutions of D_3CLi/**1**H_2 (blue; 250.1 MHz) and H_3CLi/**1**D_2 (green; 300.0 MHz). A spectrum of Li[**2**] (red; 500.2 MHz) is shown for comparison. *Note*: The orange-colored arrows mark the ^{13}C satellites of the solvent. The spectra have been recorded at different spectrometer frequencies, which leads to slight differences in the line shapes of corresponding signals and in the positions of the satellites. The integral values of the satellite signals are negligible compared to those of the other resonances. Both in the green and in the red spectrum, the integral ratio of the μ-H signal relative to the CH_2 signal is 1:2.

The reactions D_3CLi/**1**H_2 and H_3CLi/**1**D_2 afford D_3CH [$\delta(^1H) = 0.14$, sept, $^2J(H,D) = 1.9$ Hz; $\delta(^2H) = 0.14$, d, $^2J(H,D) = 1.9$ Hz] and CH_3D [$\delta(^1H) = 0.18$, t, $^2J(H,D) = 1.9$ Hz; $\delta(^2H) = 0.18$, q, $^2J(H,D) = 1.9$ Hz], respectively, as byproducts (Figure S6). The presence of small amounts of CH_4 are attributable to the fact that the Et_3SiD employed contained only 98 atom-% deuterium and to minor hydrolysis during sample preparation.

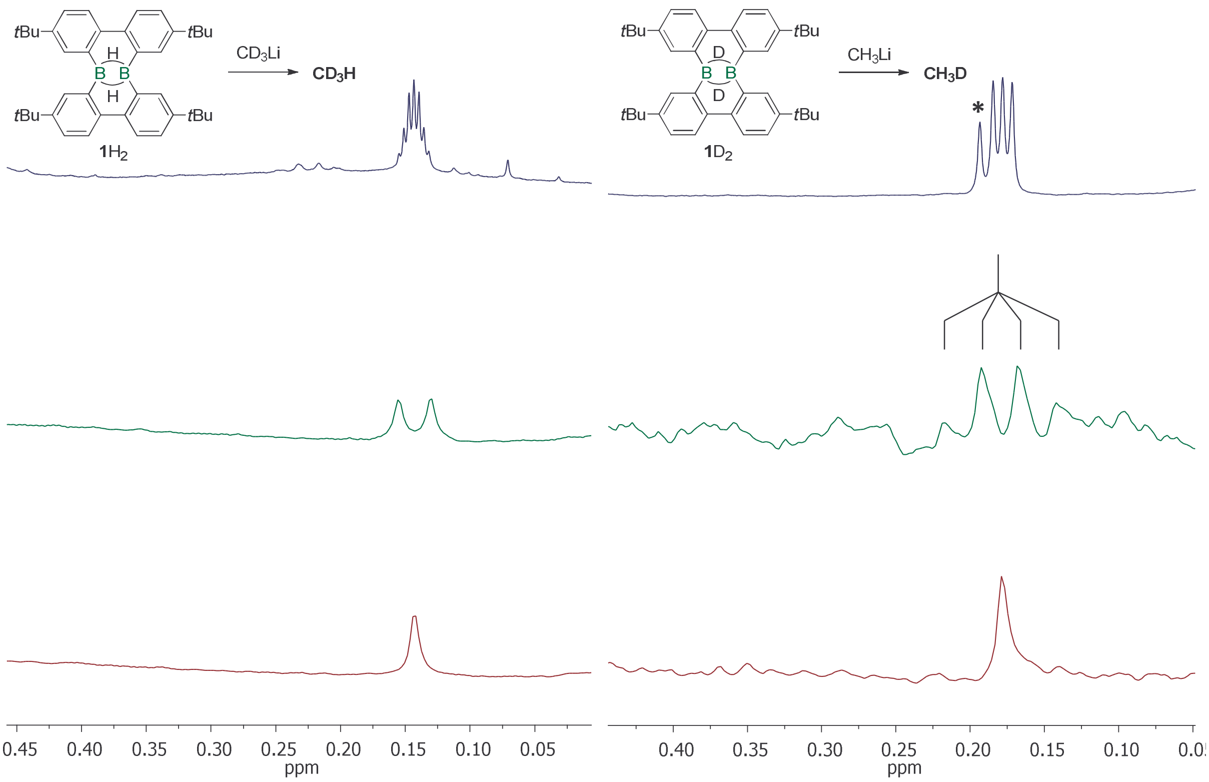

Figure S6. Stacked ^1H (blue; 500.2 MHz), ^2H (green; 76.8 MHz) and $^2H\{^1H\}$ (red) NMR spectra, recorded on the mixtures D_3CLi/**1**H_2 (left) and H_3CLi/**1**D_2 (right). The reactions were carried out in sealed NMR tubes (THF-d_8) and the spectra confirm the presence of D_3CH (left) and CH_3D (right). The signal marked with an asterisk corresponds to CH_4 (approximately 10% relative to CH_3D according to the integral values).

Reaction of Li[1H] with H_3CLi to furnish Li_2[4] and follow-up reaction with $1H_2$. A solution of H_3CLi in Et_2O (1.4 M, 15 µL, 21 µmol) was evaporated to dryness in an NMR tube. The addition of yellow [Li(thf)$_3$][**1**H] (16 mg, 21 µmol) in THF-d_8 (0.6 mL) at room temperature furnished an orange-colored solution. The NMR tube was vacuum sealed and ^{1}H and ^{11}B NMR spectra were recorded. The spectra did not show the resonances of Li[**2**], but rather signal patterns assignable to Li_2[**4**] (Figures S8 and Figures S26). The sample was transferred to a new NMR tube, which had already been charged with **1**H_2 (11 mg, 20 µmol). ^{1}H NMR spectroscopy confirmed an approximate 45% conversion to Li[**2**] and revealed the presence of a primary hydride trapping product Li[**10**], together with its rearranged isomer Li[**7**] (Figure S8).

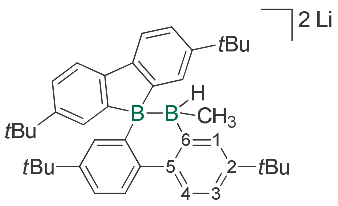

Figure S7. NMR numbering scheme for Li_2[**4**].

*Li_2[**4**]*
^{1}H NMR (300.0 MHz, THF-*d_8*): δ = 8.1 (vbr, 1H), 7.64 (d, ^3J(H,H) = 8.0 Hz, 1H; H-4), 7.53 (n.r., 1H), 7.2 (vbr, 2H), 7.19 (d, ^3J(H,H) = 7.9 Hz, 1H; H-4), 6.9 (vbr, 1H), 6.88 (dd, ^3J(H,H) = 7.9 Hz, ^4J(H,H) = 2.4 Hz, 1H; H-3), 6.76 (vbr, 2H), 1.35 (vbr, 9H; CCH_3), 1.26 (s, 9H; CCH_3), 1.20 (br, 9H; CCH_3), 1.08 (vbr, 9H; CCH_3), –0.1 (vbr, 3H; BCH_3). *Note*: An aryl resonance contributing the missing 2H is likely present at approximately 7.3 ppm, however, due to the broad line shapes and signal overlaps it cannot be unequivocally detected. Integration of the entire aryl region gives a sufficiently high integral value to match the required overall 12 aryl protons.
^{11}B NMR (96.3 MHz, THF-*d_8*): δ = –10.3 ($h_{1/2}$ = 80 Hz), –12.8 ($h_{1/2}$ = 200 Hz, *B*(H)CH_3). *Note*: Only the signal at –12.8 ppm becomes significantly sharper upon proton decoupling.

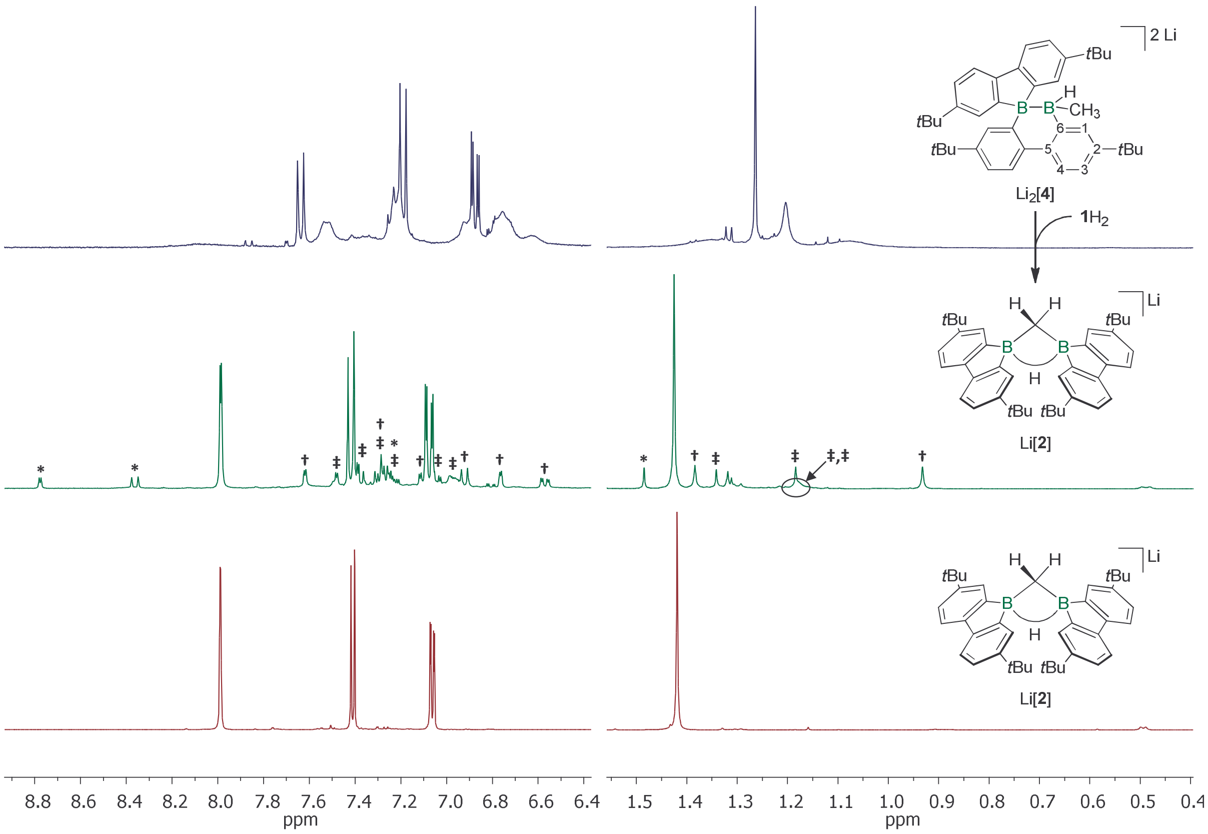

Figure S8. ^{1}H NMR spectrum of Li_2[**4**], obtained from the reaction H_3CLi/Li[**1**H] (top; 300.0 MHz). Middle: ^{1}H NMR spectrum showing the formation of Li[**2**] as major product of the reaction H_3CLi/Li[**1**H], after the addition of 1 equiv. **1**H_2 (middle; 300.0 MHz). The marked minor signals belong to Li[**1**H] (*) and the primary hydride trapping product Li[**10**] (†), which isomerizes over time to give Li[**7**] (‡). ^{1}H NMR spectrum of an authentic sample of Li[**2**] (bottom; 500.2 MHz).
All NMR spectra were recorded in THF-d_8; aromatic and alkyl regions are scaled differently.

Formation of Li_2[11]

Method A: *Reaction of Li[**1**H] with tBuCCLi.* Yellow [Li(thf)$_3$][**1**H] (20 mg, 26 µmol) in THF-*d*$_8$ (0.5 mL) was added at room temperature to an NMR tube charged with solid colorless *t*BuCCLi (2.3 mg, 26 µmol). A ^{1}H NMR spectroscopic investigation of the orange-colored solution revealed an almost quantitative consumption of the starting material and the concomitant formation of Li_2[**11**]. The reaction mixture was layered with hexane/12-crown-4 and stored at room temperature, whereupon orange single crystals of [Li(thf)(12-crown-4)][Li(thf)$_2$][**11**] formed.

Method B: *Reaction of Li$_2$[**1**] with HCCtBu***.** A twofold excess of neat *t*BuCCH (4 µL, 2.7 mg, 33 µmol) was added at room temperature to a solution of [Li(thf)$_3$]$_2$[**1**] (15 mg, 15 µmol) in THF-*d*$_8$ (0.5 mL). The progress of the slow reaction was monitored by ^{1}H NMR spectroscopy (Figure S10). After 1 month, the signal pattern of Li_2[**11**] had developed to a significant extent.

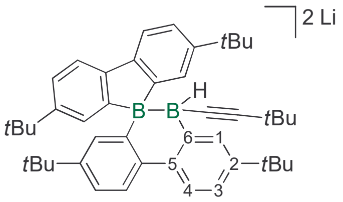

Figure S9. Schematic representation of Li_2[**11**].

*Li$_2$[**11**]*
^{1}H NMR (300.0 MHz, THF-*d*$_8$): δ = 8.2 (vbr, 1H), 7.61 (d, ^3J(H,H) = 8.0 Hz, 1H; H-4), 7.50 (n.r., 1H), 7.32 (n.r., 2H), 7.23 (d, ^3J(H,H) = 7.9 Hz, 1H; H-4), 7.12-6.95 (m, 2H), 6.82 (dd, ^3J(H,H) = 7.8 Hz, ^4J(H,H) = 2.1 Hz, 2H; H-3), 1.36 (vbr, 9H; C(CH_3)$_3$), 1.30 ppm (s, 9H; C(CH_3)$_3$), 1.19 (s, 9H; C(CH_3)$_3$), 1.09 (vbr, 9H; C(CH_3)$_3$), 0.97 (br, 9H; C≡CC(CH_3)$_3$). *Note*: An aryl resonance contributing the missing 2H cannot be unequivocally detected. Integration of the entire aryl region gives a sufficiently high integral value to match the required overall 12 aryl protons.
^{11}B NMR (96.3 MHz, THF-*d*$_8$): δ = –11.8 ($h_{1/2}$ = 60 Hz), –23.3 ($h_{1/2}$ = 190 Hz; *B*(H)CC*t*Bu). *Note*: Only the signal at –23.3 ppm becomes significantly sharper upon proton decoupling.

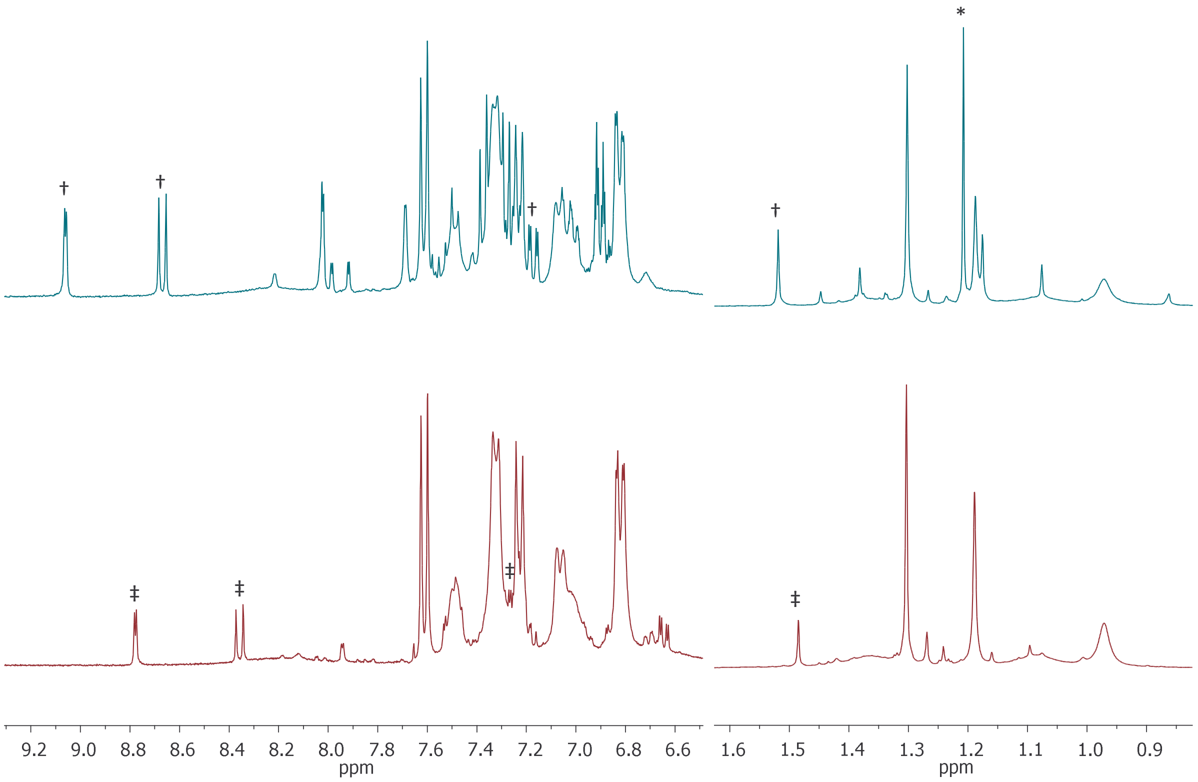

Figure S10: ^{1}H NMR spectra (300.0 MHz; THF-d_8) of the reaction mixtures 2 *t*BuCCH/Li_2[**1**] after 30 d (top) and *t*BuCCLi/Li[**1**H] after 1 d (bottom). Additional signals belong to residual Li_2[**1**] (†), Li[**1**H] (‡), and *t*BuCCH (*).

Reaction of Li_2[1] with 1 equiv. of H_3C–I. Neat H_3C–I (0.9 µL, 2.1 mg, 15 µmol) was added at room temperature with stirring to a dark red solution of $[Li(thf)_3]_2$[**1**] (15 mg, 15 µmol) in THF-d_8 (0.6 mL). A ^{1}H, ^{11}B, and ^{13}C{^{1}H} NMR spectroscopic investigation of the resulting pale yellow solution revealed the conversion of Li_2[**1**] to Li[**2**] (major product) and **13** (minor product).
For the NMR data of Li[**2**] see the reaction H_3CLi/**1**H_2.

Reaction of Li_2[1] with excess H_3CCl. Neat dark red $[Li(thf)_3]_2$[**1**] (25 mg, 25 µmol) was placed in a J. Young flask (30 mL) and dissolved in THF (2 mL). The solution was frozen at –196 °C. The flask was evacuated, closed, allowed to warm to room temperature, and filled with H_3CCl gas (1 atm), whereupon the stirred solution instantaneously changed its color from red to orange. All volatiles were removed in a dynamic vacuum and the orange solid residue was dissolved in THF-d_8. A ^{1}H and ^{11}B NMR spectroscopic investigation revealed the quantitative conversion of Li_2[**1**] to Li[**2**]. The entire sample was transferred to a small vial, which was placed in a larger vessel containing hexane (2 mL). The outer vessel was covered with a lid to allow for gas-phase diffusion of the solvents in a closed environment. Colorless crystalline material of $[Li(thf)_4]$[**2**] (17 mg, 20 µmol, 80%) was obtained. For the NMR data of Li[**2**], see the reaction H_3CLi/**1**H_2.

Reaction of Li_2[1] with 3 equiv. of H_3C–I. Neat H_3C–I (2.8 µL, 6.4 mg, 45 µmol) was added at room temperature with stirring to a dark red solution of $[Li(thf)_3]_2$[**1**] (15 mg, 15 µmol) in THF-d_8 (0.6 mL). A ^{1}H, ^{11}B, and ^{13}C{^{1}H} NMR spectroscopic investigation of the resulting pale yellow solution revealed the quantitative conversion of Li_2[**1**] to **13**.

Reaction of Li_2[1] with 2 equiv. of methyl triflate. Neat methyl triflate (2.8 µL, 4.0 mg, 25 µmol) was added at room temperature with stirring to a dark red solution of $[Li(thf)_3]_2$[**1**] (12 mg, 12 µmol) in C_6H_6 (0.5 mL). All volatiles were removed in a dynamic vacuum and the residue was dissolved in THF-d_8. A ^{1}H, ^{11}B, and ^{13}C{^{1}H} NMR spectroscopic investigation of the yellow solution confirmed the quantitative conversion of Li_2[**1**] to **13**. The addition of only 1.1 equiv. of methyl triflate to $[Li(thf)_3]_2$[**1**] in C_6H_6 furnished Li[**2**] as the major product, which crystallizes as at room temperature from the reaction solution in the form of $[Li(thf)_3]$[**2**]. For the NMR data of Li[**2**], see the reaction H_3CLi/**1**H_2.

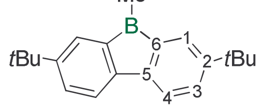

Figure S11. NMR numbering scheme for **13**.

13
^{1}H NMR (500.2 MHz, THF-d_8): δ = 7.50 (dd, ^4J(H,H) = 2.0 Hz, ^5J(H,H) = 0.6 Hz, 2H; H-1), 7.38 (dd, ^3J(H,H) = 7.9 Hz, ^5J(H,H) = 0.6 Hz, 2H; H-4), 7.16 (dd, ^3J(H,H) = 7.9 Hz, ^4J(H,H) = 2.0 Hz, 2H; H-3), 1.33 (s, 18H; CCH_3), 0.31 (s, 3H; BCH_3).
^{11}B NMR (160.5 MHz, THF-d_8): δ = 13.7.
^{13}C{^{1}H} NMR (125.8 MHz, THF-d_8): δ = 152.6 (br, C-6), 148.6 (C-2), 147.3 (C-5), 127.3 (C-1), 124.5 (C-3), 118.5 (C-4), 34.9 (*C*CH_3), 31.9 (CH_3), 3.2 (BCH_3).
Note: An authentic sample of **13**, prepared from 2,7-di(*t*Bu)-9-Br-9-borafluorene and H_3CMgI in Et_2O/toluene, gave identical NMR shift values.

Reaction of Li_2[1] with 1 equiv. ethyl bromide. Neat ethyl bromide (1.3 µL, 1.9 mg, 17 µmol) was added at room temperature with stirring to a dark red solution of $[Li(thf)_3]_2$[**1**] (15 mg, 15 µmol) in THF-d_8 (0.5 mL). A ^{1}H, ^{11}B, and ^{13}C{^{1}H} NMR spectroscopic investigation of the resulting yellow solution revealed the quantitative conversion of Li_2[**1**] to Li[**19**].

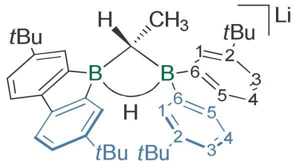

Figure S12. NMR numbering scheme for Li[**19**].

*Li[**19**]*

^{1}H NMR (500.2 MHz, THF-*d*$_8$): δ = 8.14 (d, ^{4}J(H,H) = 1.6 Hz, 2H; H-1), 7.99 (d, ^{4}J(H,H) = 1.6 Hz, 2H; H-1), 7.45 (d, ^{3}J(H,H) = 7.8 Hz, 2H; H-4), 7.42 (d, ^{3}J(H,H) = 7.8 Hz, 2H; H-4), 7.09 (dd, ^{3}J(H,H) = 7.8 Hz, ^{4}J(H,H) = 1.6 Hz, 2H; H-3), 7.06 (dd, ^{3}J(H,H) = 7.8 Hz, ^{4}J(H,H) = 1.6 Hz, 2H; H-3), 2.02 (br, 1H; μ-H), 1.47 (d, ^{3}J(H,H) = 7.0 Hz, 3H; CHC*H*$_3$), 1.44 (s, 18H, C(CH$_3$)$_3$), 1.43 (s, 18H, C(CH$_3$)$_3$), 0.95 (qd, ^{3}J(H,H) = 7.0 Hz, ^{3}J(H,H) = 3.0 Hz, 1H; C*H*CH$_3$).

^{7}Li NMR (116.6 MHz, THF-*d*$_8$): δ = –1.8.

^{11}B NMR (160.5 MHz, THF-*d*$_8$): δ = –12.6 (br).

^{13}C{^{1}H} NMR (125.8 MHz, THF-*d*$_8$): δ = 159.6 (C-6), 156.5 (C-6), 148.3 (C-5), 147.4 (C-5), 146.3 (C-2), 146.0 (C-2), 130.1 (C-1), 127.9 (C-1), 121.7 (C-3), 121.6 (C-3), 117.7 (C-4), 117.6 (C-4), 35.2 (*C*(CH$_3$)$_3$), 35.2 (*C*(CH$_3$)$_3$), 32.6 (C(*C*H$_3$)$_3$), 14.3 (CH*C*H$_3$), 11.0 (br, *C*HCH$_3$).

Note: The tentative assignment of resonances to the blue *vs* black aromatic rings is based on 2D NMR experiments and a comparison with the NMR spectra of the corresponding compound Li[**2**] featuring a symmetrical CH$_2$ bridge.

Reaction of Li_2[1] with benzyl chloride. A dark red solution of $[Li(thf)_3]_2$[**1**] (30 mg, 30 µmol) in THF-d_8 (0.3 mL) was added dropwise with stirring at room temperature to benzyl chloride (4.1 µL, 4.5 mg, 36 µmol) in THF-d_8 (0.2 mL). A ^{1}H, ^{11}B, and ^{13}C{^{1}H} NMR spectroscopic investigation of the resulting yellow solution revealed the selective conversion of Li_2[**1**] to Li[**16**] (> 90%). Upon addition of the reaction mixture to hexane (2 mL), a colorless precipitate formed. The mother liquor was removed, the precipitate washed with hexane (3 x 0.2 mL), and dried under vacuum to obtain pure Li[**16**] (according to NMR spectroscopy). Colorless single crystals of $[Li(thf)_4]$[**16**] were grown by gas-phase diffusion of hexane into a THF solution of Li[**16**] (3 d, room temperature). *Note*: Li[**16**] was also observed as the major product (> 90%) when 1 equiv. of benzyl bromide was used instead of benzyl chloride.

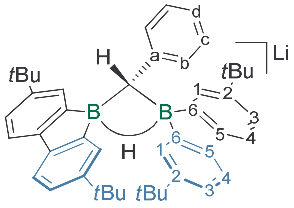

Figure S13. NMR numbering scheme for Li[**16**].

*Li[**16**]*
^{1}H NMR (500.2 MHz, THF-*d*$_8$): δ = 8.09 (n.r., 2H; H-1), 7.51 (n.r., 2H; H-1), 7.45 (d, ^{3}J(H,H) = 7.9 Hz, 2H; H-4), 7.43 (d, ^{3}J(H,H) = 7.9 Hz, 2H; H-4), 7.10 (dd, ^{3}J(H,H) = 7.9 Hz, ^{4}J(H,H) = n.r., 2H; H-3), 7.03-7.02 (m, 4H; H-3, H-b), 6.80-6.77 (m, 2H; H-c), 6.71-6.68 (m, 1H; H-d), 2.73 (s, 1H; BCH), 2.26 (br, 1H; BHB), 1.44 (s, 18H; CH_3), 1.23 (s, 18H; CH_3).
^{11}B NMR (160.5 MHz, THF-*d*$_8$): δ = −13.1 (br).
^{13}C{^{1}H} NMR (125.8 MHz, THF-*d*$_8$): δ = 158.4 (br; C-6), 155.5 (br; C-6), 150.7 (C-a), 148.1 (C-5), 147.8 (C-5), 146.4 (C-2), 145.8 (C-2), 134.6 (C-b), 131.5 (C-1), 127.8 (C-1), 126.4 (C-c), 122.1 (C-3), 121.8 (C-d), 121.7 (C-3), 117.7 (C-4), 117.5 (C-4), 35.2 (CCH$_3$), 35.0 (CCH$_3$), 32.6 (CH_3), 32.4 (CH_3), 27.3 (br; BCH).

Reaction of Li_2[1] with the radical clock (bromomethyl)cyclopropane. A dark red solution of $[Li(thf)_3]_2$[**1**] (12 mg, 12 µmol) in THF-d_8 (0.3 mL) was added dropwise with stirring at room temperature to (bromomethyl)cyclopropane (1.4 µL, 1.9 mg, 14 µmol) in THF-d_8 (0.2 mL). A ^{1}H, ^{11}B, and ^{13}C{^{1}H} NMR spectroscopic investigation of the resulting yellow solution revealed the selective conversion of Li_2[**1**] to Li[**17**] (> 90%). No signals were observed in the allylic region of the ^{1}H NMR spectrum. Note: Li[**17**] decomposes in THF-d_8 at room temperature within days.

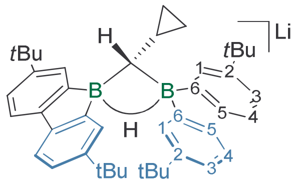

Figure S14. NMR numbering scheme for Li[**17**].

*Li[**17**]*

^{1}H NMR (300.0 MHz, THF-d_8): δ = 8.27 (d, ^4J(H,H) = 1.8 Hz, 2H; H-1), 7.92 (d, ^4J(H,H) = 1.8 Hz, 2H; H-1), 7.44 (d, ^3J(H,H) = 7.9 Hz, 2H; H-4), 7.41 (d, ^3J(H,H) = 7.9 Hz, 2H; H-4), 7.08 (dd, ^3J(H,H) = 7.9 Hz, ^4J(H,H) = 1.8 Hz, 2H; H-3), 7.05 (dd, ^3J(H,H) = 7.9 Hz, ^4J(H,H) = 1.8 Hz, 2H; H-3), 2.09 (br, 1H; BHB), 1.58-1.50 (m, 1H; CH_2CH), 1.44 (s, 18H; CH_3), 1.41 (s, 18H; CH_3), 0.86 (dd, ^3J(H,H) = 6.3 Hz, ^3J(H,H) = 2.9 Hz, 1H; BCH), 0.25-0.19 (m, 2H; CH_2), –0.14-(–0.19) (m, 2H; CH_2).

^{11}B NMR (96.3 MHz, THF-d_8): δ = –14.9 (br).

^{13}C{^{1}H} NMR (125.8 MHz, THF-d_8): δ = 159.4 (C-6), 156.8 (C-6), 148.2 (C-5), 147.5 (C-5), 146.4 (C-2), 145.9 (C-2), 130.6 (C-1), 127.7 (C-1), 121.8 (C-3), 121.6 (C-3), 117.7 (C-4), 117.6 (C-4), 35.2 (CCH_3) 32.6 (CH_3), 24.6 (br; BCH), 13.1 ($CHCH_2$), 9.5 (CH_2).

Reactions of Li_2[1] with α,ω-dihaloalkanes

All reactions were conducted by adding a dark red solution of $[Li(thf)_3]_2$[**1**] in THF-d_8 (0.3 mL) at room temperature with stirring to a small excess of an α,ω-dihaloalkane $X(CH_2)_nX$ in THF-d_8 (0.2 mL). The amounts of starting materials used in each individual experiment and the products obtained are listed in Table S1.

Table S1: Starting materials and products of the reactions of α,ω-dihaloalkanes $X(CH_2)_nX$ with $[Li(thf)_3]_2$[**1**]. The percentages of conversion of the boron compound (determined through integration of the ^{1}H NMR spectra) are listed in parentheses.

	starting materials	product(s)
	**1,2-dihaloethane**	
Cl	1,2-dichloroethane (1.7 µL, 2.1 mg, 21 µmol), $[Li(thf)_3]_2$[**1**] (17 mg, 17 µmol)	**14**^{C2} (100%)
Br	1,2-dibromoethane (1.8 µL, 3.9 mg, 21 µmol), $[Li(thf)_3]_2$[**1**] (17 mg, 17 µmol)	**14**^{C2} (100%)
	**1,3-dihalopropane**	
Cl	1,3-dichloropropane (3.2 µL, 3.8 mg, 34 µmol), $[Li(thf)_3]_2$[**1**] (30 mg, 30 µmol)	Complex mixture of yet unidentified products
Br	1,3-dibromopropane (3.6 µL, 7.2 mg, 35 µmol), $[Li(thf)_3]_2$[**1**] (30 mg, 30 µmol)	**14**^{C3} (> 90%; yield: 15 mg, 25 µmol, 83%)
	**1,4-dihalobutane**	
Cl	1,4-dichlorobutane (3.9 µL, 4.5 mg, 36 µmol), $[Li(thf)_3]_2$[**1**] (30 mg, 30 µmol)	Li[**15**^{C4,Cl}] (92%; yield: 17 mg, 21 µmol, 70% (calcd for $[Li(thf)_{2.5}]$[**15**^{C4,Cl}])), **14**^{C4} (8%)
Br	1,4-dibromobutane (6.0 µL, 11 mg, 50 µmol), $[Li(thf)_3]_2$[**1**] (42 mg, 42 µmol)	**14**^{C4} (92%), Li[**15**^{C4,Br}] (8%)
	**1,5-dihalopentane**	
Cl	1,5-dichloropentane (4.4 µL, 4.8 mg, 34 µmol), $[Li(thf)_3]_2$[**1**] (29 mg, 29 µmol)	Li[**15**^{C5,Cl}] (> 90%)
Br	1,5-dibromopentane (4.8 µL, 8.1 mg, 35 µmol), $[Li(thf)_3]_2$[**1**] (30 mg, 30 µmol)	Li[**15**^{C5,Br}] (> 90%)
	**1,6-dihalohexane**	
Cl	1,6-dichlorohexane (5.0 µL, 5.3 mg, 34 µmol), $[Li(thf)_3]_2$[**1**] (29 mg, 29 µmol)	Li[**15**^{C6,Cl}] (> 90%)
Br	1,6-dibromohexane (5.0 µL, 7.9 mg, 33 µmol), $[Li(thf)_3]_2$[**1**] (28 mg, 28 µmol)	Li[**15**^{C6,Br}] (> 90%)

The neutral compounds **14**^{C2}, **14**^{C3}, and **14**^{C4} were crystallized by slow evaporation of the solvent. The crystals were rinsed with small volumes of hexane (**14**^{C2}: 0 mL, **14**^{C3}: 3 x 0.2 mL, **14**^{C4}: 3 x 0.2 mL) and THF (**14**^{C2}: 0 mL, **14**^{C3} 2 x 0.1 mL, **14**^{C4}: 0 mL), and dried in a dynamic vacuum. The salts Li[**15**^{C4,Cl}], Li[**15**^{C4,Br}], Li[**15**^{C5,Cl}], Li[**15**^{C5,Br}], Li[**15**^{C6,Cl}], and Li[**15**^{C6,Br}] were precipitated from the reaction mixtures by addition of hexane (2 mL). The precipitates were washed with hexane (3 x 0.2 mL) and dried in a dynamic vacuum. Colorless single crystals of $[Li(12\text{-crown-}4)_2]$[**15**^{C5,Cl}] were obtained by layering a solution of Li[**15**^{C5,Cl}] in THF with hexane/12-crown-4 (3 d; room temperature).

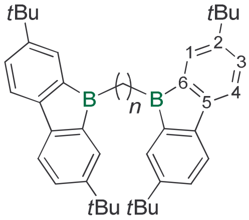

Figure S15. NMR numbering scheme for **14**^{Cn}.

***14**^{C2}*

^{1}H NMR (500.2 MHz, THF-*d*$_8$): δ = 7.66 (d, ^{4}J(H,H) = 1.9 Hz, 4H; H-1), 7.39 (d, ^{3}J(H,H) = 7.8 Hz, 4H; H-4), 7.17 (dd, ^{3}J(H,H) = 7.8 Hz, ^{4}J(H,H) = 1.9 Hz, 4H; H-3), 1.35 (s, 36H; CH_3), 1.07 (s, 4H; C_2H_4).
^{11}B NMR (160.5 MHz, THF-*d*$_8$): δ = 15.9.
^{13}C{^{1}H} NMR (125.8 MHz, THF-*d*$_8$): δ = 153.0 (C-6), 148.2 (C-2), 147.6 (C-5), 128.0 (C-1), 124.3 (C-3), 118.5 (C-4), 34.9 (*C*CH_3), 32.0 (CH_3), 16.1 (C_2H_4).

***14**^{C3}*

^{1}H NMR (500.2 MHz, THF-*d*$_8$): δ = 7.48 (d, ^{4}J(H,H) = 1.9 Hz, 4H; H-1), 7.37 (d, ^{3}J(H,H) = 7.9 Hz, 4H; H-4), 7.13 (dd, ^{3}J(H,H) = 7.9 Hz, ^{4}J(H,H) = 1.9 Hz, 4H; H-3), 1.53-1.47 (m, 2H; CCH_2), 1.30 (s, 36H; CH_3), 0.95-0.92 (m, 4H; BCH_2).
^{11}B NMR (160.5 MHz, THF-*d*$_8$): δ = 15.4 (vbr).
^{13}C{^{1}H} NMR (125.8 MHz, THF-*d*$_8$): δ = 152.7 (br; C-6), 148.3 (C-2), 147.6 (C-5), 128.1 (C-1), 124.2 (C-3), 118.4 (C-4), 34.9 (*C*CH_3), 32.0 (CH_3), 26.0 (br; BCH_2), 23.0 (C*C*H_2).

***14**^{C4}*

^{1}H NMR (500.2 MHz, THF-*d*$_8$): δ = 7.51 (d, ^{4}J(H,H) = 1.9 Hz, 4H; H-1), 7.37 (d, ^{3}J(H,H) = 7.9 Hz, 4H; H-4), 7.14 (dd, ^{3}J(H,H) = 7.9 Hz, ^{4}J(H,H) = 1.9 Hz, 4H; H-3), 1.32 (s, 36H; CH_3), 1.32-1.29 (m, 4H; $BCCH_2$), 0.92-0.90 (m, 4H; BCH_2).
^{11}B NMR (160.5 MHz, THF-*d*$_8$): δ = 14.4 (vbr).
^{13}C{^{1}H} NMR (125.8 MHz, THF-*d*$_8$): δ = 152.4 (br; C-6), 148.3 (C-2), 147.6 (C-5), 127.9 (C-1), 124.3 (C-3), 118.4 (C-4), 34.9 (*C*CH_3), 32.0 (CH_3), 30.9 (BC*C*H_2), 21.5 (br; BCH_2).

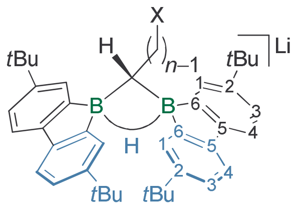

Figure S16. NMR numbering scheme for **15**^{Cn,X}.

*Li[**15**^{C4,Cl}]*

^{1}H NMR (500.2 MHz, THF-*d*$_8$): δ = 8.15 (d, ^{4}J(H,H) = 1.9 Hz, 2H; H-1), 8.01 (d, ^{4}J(H,H) = 1.9 Hz, 2H; H-1), 7.46 (d, ^{3}J(H,H) = 7.9 Hz, 2H; H-4), 7.43 (d, ^{3}J(H,H) = 7.9 Hz, 2H; H-4), 7.10 (dd, ^{3}J(H,H) = 7.9 Hz, ^{4}J(H,H) = 1.9 Hz, 2H; H-3), 7.07 (dd, ^{3}J(H,H) = 7.9 Hz, ^{4}J(H,H) = 1.9 Hz, 2H; H-3), 3.19 (t, ^{3}J(H,H) = 7.4 Hz, 2H; $ClCH_2$), 2.16 (vq, ^{3}J(H,H) = 7.4 Hz, 2H; $BCCH_2$), 2.04 (br, 1H; BHB), 1.52 (vquint, ^{3}J(H,H) = 7.4 Hz, 2H; $ClCCH_2$), 1.45 (s, 18H; CH_3), 1.43 (s, 18H; CH_3), 0.94 (dt, ^{3}J(H,H) = 7.8 Hz, ^{3}J(H,H) = 2.6 Hz, 1H; BCH).

^{11}B NMR (160.5 MHz, THF-*d*$_8$): δ = −13.0 (br).

^{13}C{^{1}H} NMR (125.8 MHz, THF-*d*$_8$): δ = 159.0 (br; C-6), 156.4 (br; C-6), 148.3 (C-5), 147.4 (C-5), 146.5 (C-2), 146.1 (C-2), 129.8 (C-1), 127.8 (C-1), 121.8 (C-3), 121.7 (C-3), 117.8 (C-4), 117.7 (C-4), 46.8 ($ClCH_2$), 38.8 ($ClCCH_2$), 35.3 (CCH_3), 35.2 (CCH_3), 32.7 (CH_3 or CH_3), 32.6 (CH_3 or CH_3), 28.2 ($BCCH_2$), 19.3 (br; BCH).

*Li[**15**^{C5,Cl}]*

^{1}H NMR (500.2 MHz, THF-*d*$_8$): δ = 8.16 (d, ^{4}J(H,H) = 1.8 Hz, 2H; H-1), 8.01 (d, ^{4}J(H,H) = 1.8 Hz, 2H; H-1), 7.46 (d, ^{3}J(H,H) = 7.9 Hz, 2H; H-4), 7.42 (d, ^{3}J(H,H) = 7.9 Hz, 2H; H-4), 7.09 (dd, ^{3}J(H,H) = 7.9 Hz, ^{4}J(H,H) = 1.8 Hz, 2H; H-3), 7.07 (dd, ^{3}J(H,H) = 7.9 Hz, ^{4}J(H,H) = 1.8 Hz, 2H; H-3), 3.14 (t, ^{3}J(H,H) = 7.3 Hz, 2H; $ClCH_2$), 2.07 (vq, ^{3}J(H,H) = 7.5 Hz, 2H; $BCCH_2$), 2.04 (br, 1H; BHB), 1.51 (vquint, ^{3}J(H,H) = 7.3 Hz, 2H; $ClCCH_2$), 1.45 (s, 18H; CH_3), 1.43 (s, 18H; CH_3), 1.14 (vquint, ^{3}J(H,H) = 7.3 Hz, 2H; $ClCCCH_2$), 0.93 (td, ^{3}J(H,H) = 7.6 Hz, ^{3}J(H,H) = 2.3 Hz, 1H; BCH).

^{11}B NMR (160.5 MHz, THF-*d*$_8$): δ = −13.7 (br).

^{13}C{^{1}H} NMR (125.8 MHz, THF-*d*$_8$): δ = 159.3 (br; C-6), 156.6 (br; C-6), 148.3 (C-5), 147.3 (C-5), 146.4 (C-2), 146.1 (C-2), 129.8 (C-1), 127.8 (C-1), 121.7 (C-3), 121.7 (C-3), 117.8 (C-4), 117.7 (C-4), 46.0 ($ClCH_2$), 35.3 (CCH_3), 35.2 (CCH_3), 34.1 ($ClCCH_2$), 32.7 (CH_3, CH_3), 32.4 ($ClCCCH_2$), 29.7 ($BCCH_2$), 20.4 (br; BCH).

*Li[**15**^{C5,Br}]*

^{1}H NMR (500.2 MHz, THF-*d*$_8$): δ = 8.15 (d, ^{4}J(H,H) = 1.8 Hz, 2H; H-1), 8.01 (d, ^{4}J(H,H) = 1.8 Hz, 2H; H-1), 7.46 (d, ^{3}J(H,H) = 7.9 Hz, 2H; H-4), 7.43 (d, ^{3}J(H,H) = 7.9 Hz, 2H; H-4), 7.10 (dd, ^{3}J(H,H) = 7.9 Hz, ^{4}J(H,H) = 1.8 Hz, 2H; H-3), 7.07 (dd, ^{3}J(H,H) = 7.9 Hz, ^{4}J(H,H) = 1.8 Hz, 2H; H-3), 3.03 (t, ^{3}J(H,H) = 7.3 Hz, 2H; $BrCH_2$), 2.07 (vq, ^{3}J(H,H) = 7.5 Hz, 2H; $BCCH_2$), 2.04 (br, 1H; BHB), 1.60 (vquint, ^{3}J(H,H) = 7.3 Hz, 2H; $BrCCH_2$), 1.45 (s, 18H; CH_3), 1.43 (s, 18H; CH_3), 1.15 (vquint, ^{3}J(H,H) = 7.3 Hz, 2H; $BrCCCH_2$), 0.93 (td, ^{3}J(H,H) = 7.6 Hz, ^{3}J(H,H) = 2.3 Hz, 1H; BCH).

^{11}B NMR (160.5 MHz, THF-*d*$_8$): δ = −12.9 (br).

^{13}C{^{1}H} NMR (125.8 MHz, THF-*d*$_8$): δ = 159.4 (br; C-6), 156.6 (br; C-6), 148.2 (C-5), 147.3 (C-5), 146.5 (C-2), 146.1 (C-2), 129.8 (C-1), 127.8 (C-1), 121.8 (C-3), 121.7 (C-3), 117.8 (C-4), 117.7 (C-4), 35.3 (CCH_3), 35.2 ($BrCH_2$, CCH_3), 34.3 ($BrCCH_2$), 33.7 ($BrCCCH_2$), 32.7 (CH_3), 29.5 ($BCCH_2$), 20.4 (br; BCH).

*Li[**15**^{C6,Cl}]*

^{1}H NMR (500.2 MHz, THF-*d*$_8$): δ = 8.17 (d, ^{4}J(H,H) = 1.8 Hz, 2H; H-1), 8.01 (d, ^{4}J(H,H) = 1.8 Hz, 2H; H-1), 7.45 (d, ^{3}J(H,H) = 7.9 Hz, 2H; H-4), 7.42 (d, ^{3}J(H,H) = 7.9 Hz, 2H; H-4), 7.09 (dd, ^{3}J(H,H) = 7.9 Hz, ^{4}J(H,H) = 1.8 Hz, 2H; H-3), 7.06 (dd, ^{3}J(H,H) = 7.9 Hz, ^{4}J(H,H) = 1.8 Hz, 2H; H-3), 3.15 (t, ^{3}J(H,H) = 7.1 Hz, 2H; $ClCH_2$), 2.05 (vq, ^{3}J(H,H) = 7.5 Hz, 2H; $BCCH_2$), 2.04 (br, 1H; BHB), 1.45 (s, 18H; CH_3), 1.44 (vquint, ^{3}J(H,H) = 7.2 Hz, 2H; $ClCCH_2$), 1.43 (s, 18H; CH_3), 1.16 (vquint, ^{3}J(H,H) = 7.5 Hz, 2H; $ClCCCH_2$), 1.07 (vquint, ^{3}J(H,H) = 7.3 Hz, 2H; $BCCCH_2$), 0.94 (td, ^{3}J(H,H) = 7.6 Hz, ^{4}J(H,H) = 2.6 Hz, 1H; BCH).

^{11}B NMR (160.5 MHz, THF-*d*$_8$): δ = −14.0 (br).

^{13}C{^{1}H} NMR (125.8 MHz, THF-*d*$_8$): δ = 159.6 (br; C-6), 156.8 (br; C-6), 148.2 (C-5), 147.4 (C-5), 146.4 (C-2), 146.0 (C-2), 129.9 (C-1), 127.8 (C-1), 121.6 (C-3), 121.6 (C-3), 117.7 (C-4), 117.6 (C-4), 45.8 ($ClCH_2$), 35.3 (*C*CH_3), 35.2 (*C*CH_3), 34.4 ($BCCCH_2$), 33.8 ($ClCCH_2$), 32.7 (CH_3), 30.2 ($BCCH_2$), 27.7 ($ClCCCH_2$), 20.6 (br; BCH).

*Li[**15**^{C6,Br}]*

^{1}H NMR (500.2 MHz, THF-*d*$_8$): δ = 8.16 (d, ^{4}J(H,H) = 1.8 Hz, 2H; H-1), 8.01 (d, ^{4}J(H,H) = 1.8 Hz, 2H; H-1), 7.46 (d, ^{3}J(H,H) = 7.9 Hz, 2H; H-4), 7.43 (d, ^{3}J(H,H) = 7.9 Hz, 2H; H-4), 7.10 (dd, ^{3}J(H,H) = 7.9 Hz, ^{4}J(H,H) = 1.8 Hz, 2H; H-3), 7.07 (dd, ^{3}J(H,H) = 7.9 Hz, ^{4}J(H,H) = 1.8 Hz, 2H; H-3), 3.05 (t, ^{3}J(H,H) = 7.2 Hz, 2H; $BrCH_2$), 2.05 (vq, ^{3}J(H,H) = 7.5 Hz, 2H; $BCCH_2$), 2.03 (br, 1H; BHB), 1.54 (vquint, ^{3}J(H,H) = 7.3 Hz, 2H; $BrCCH_2$), 1.45 (s, 18H; CH_3), 1.44 (s, 18H; CH_3), 1.17 (vquint, ^{3}J(H,H) = 7.3 Hz, 2H; $BrCCCH_2$), 1.07 (vquint, ^{3}J(H,H) = 7.3 Hz, 2H; $BCCCH_2$), 0.94 (td, ^{3}J(H,H) = 7.5 Hz, ^{3}J(H,H) = 2.2 Hz, 1H; BCH).

^{11}B NMR (160.5 MHz, THF-*d*$_8$): δ = −13.6 (br).

^{13}C{^{1}H} NMR (125.8 MHz, THF-*d*$_8$): δ = 159.5 (br; C-6), 156.8 (br; C-6), 148.2 (C-5), 147.3 (C-5), 146.5 (C-2), 146.1 (C-2), 129.9 (C-1), 127.9 (C-1), 121.7 (C-3), 121.7 (C-3), 117.8 (C-4), 117.7 (C-4), 35.3 (*C*CH_3), 35.2 (*C*CH_3), 34.8 ($BrCH_2$), 34.2 (BCC*C*H_2), 34.1 (BrC*C*H_2), 32.7 (CH_3, CH_3), 30.2 ($BCCH_2$), 29.0 (BrCC*C*H_2), 20.7 (br; BCH).

2. Plots of NMR spectra

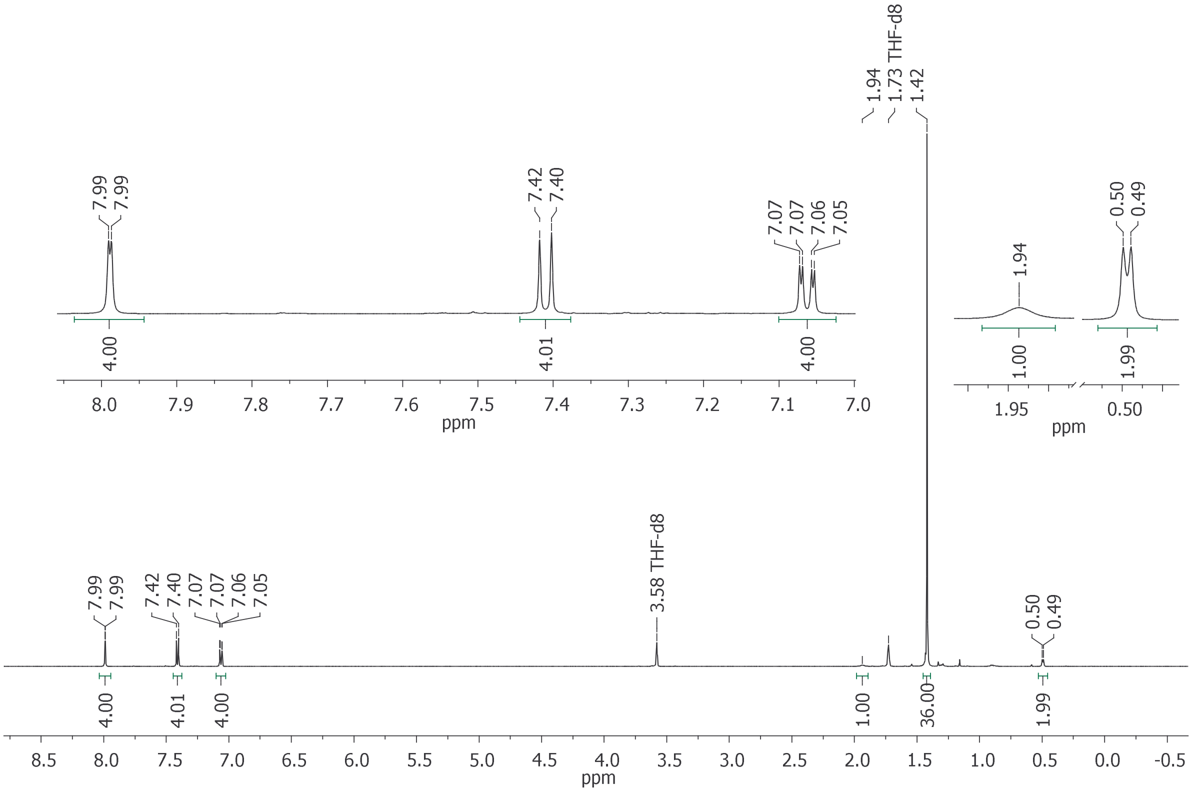

Figure S17: ^{1}H NMR spectrum of Li[**2**] (500.2 MHz, THF-d_8).

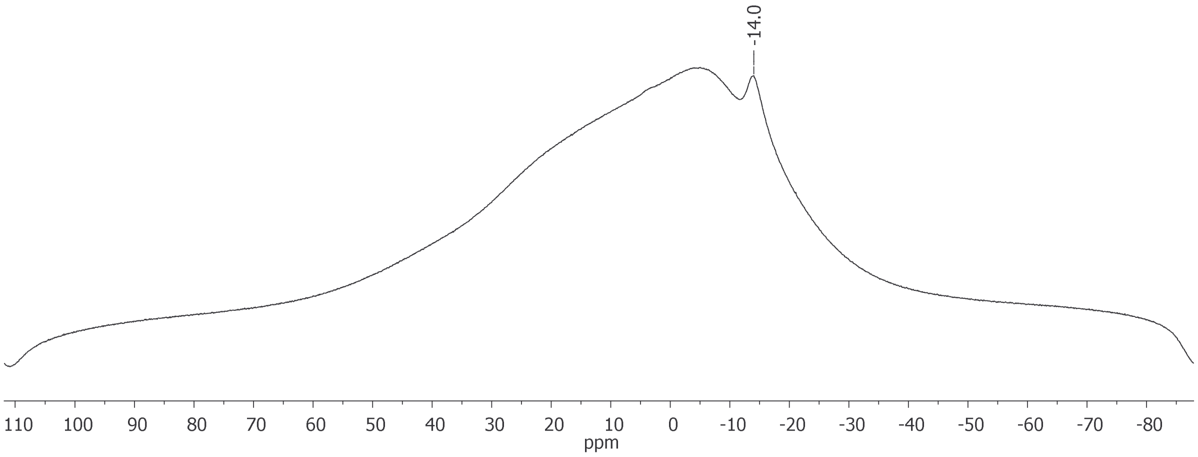

Figure S18: ^{11}B NMR spectrum of Li[**2**] (160.5 MHz, THF-d_8).

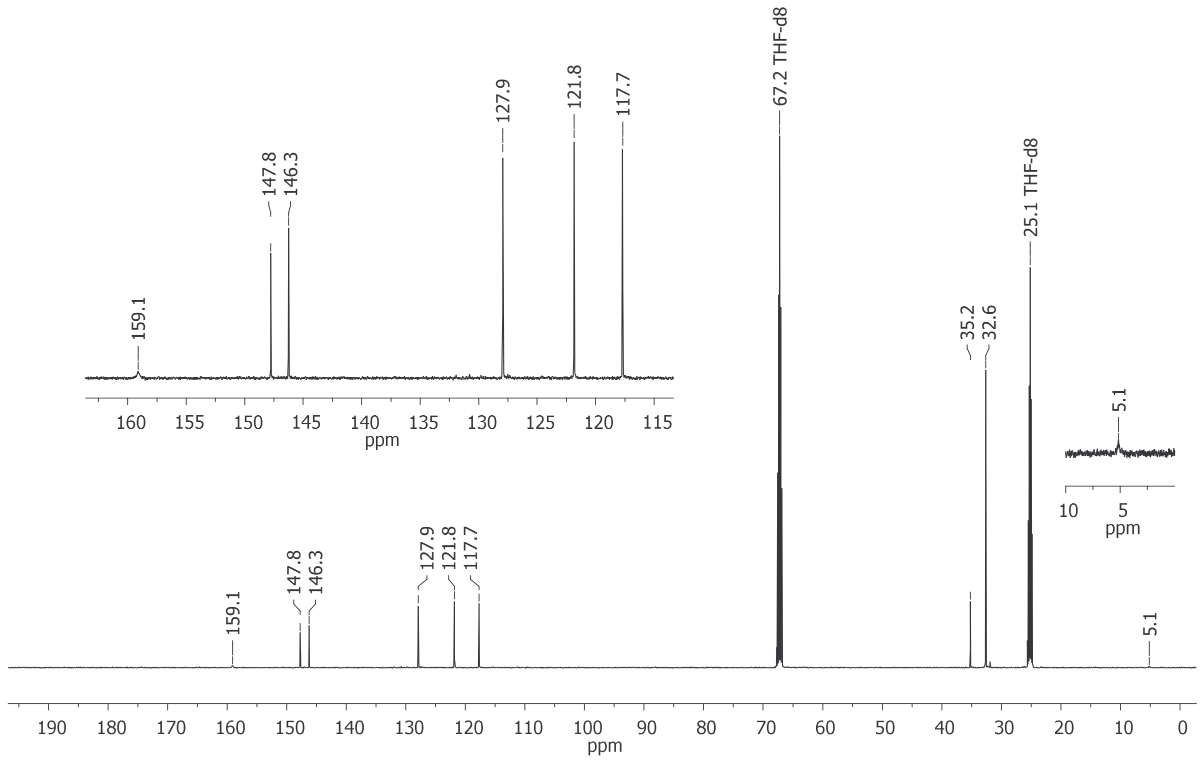

Figure S19: $^{13}C\{^1H\}$ NMR spectrum of Li[**2**] (125.8 MHz, THF-d_8).

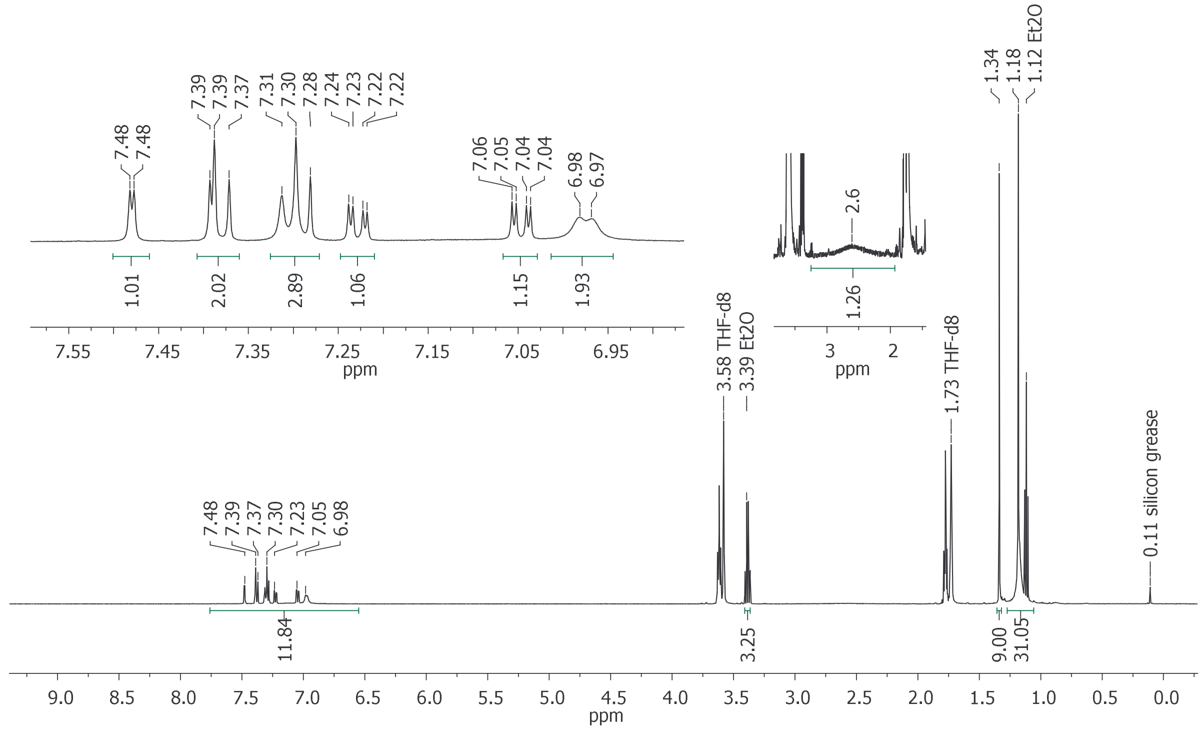

Figure S20: ^1H NMR spectrum of Li[**7**] (500.2 MHz, THF-d_8).

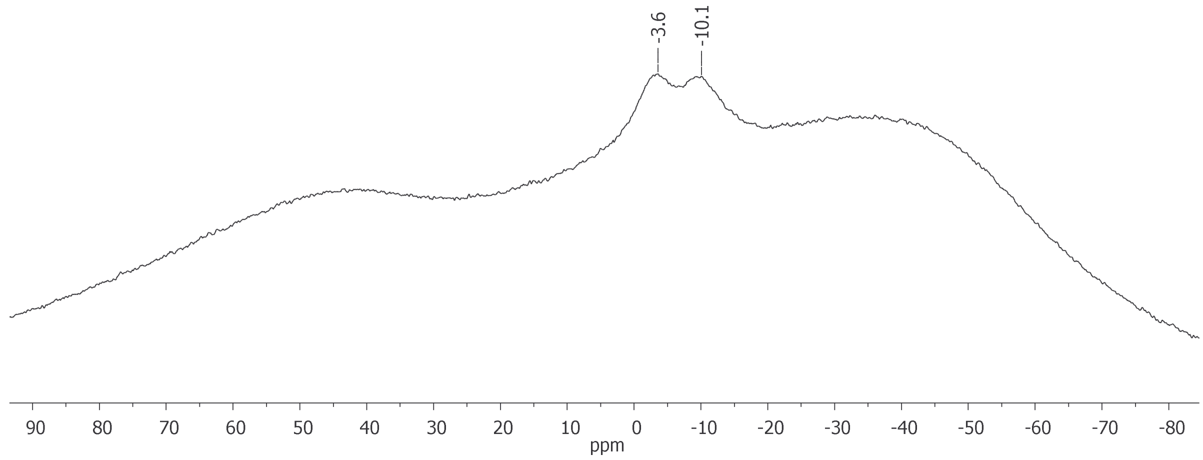

Figure S21: ^{11}B NMR spectrum of Li[**7**] (96.3 MHz, THF-d_8).

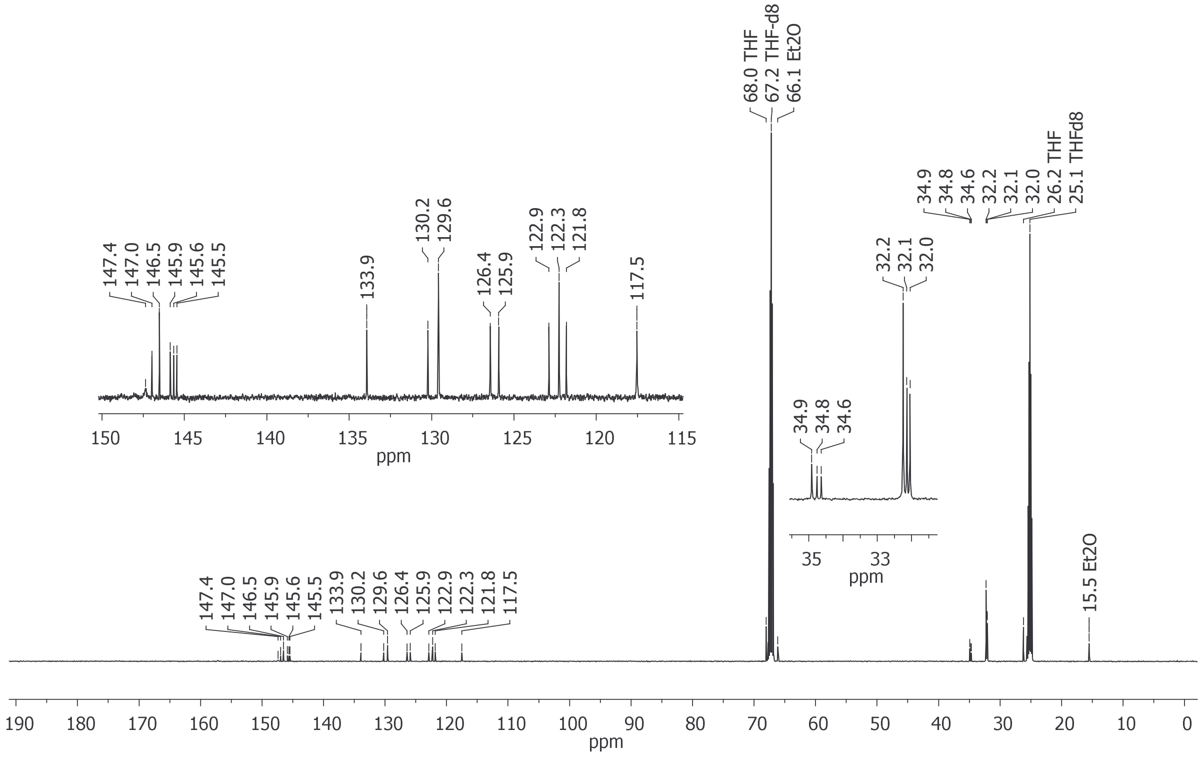

Figure S22: ^{13}C{^{1}H} NMR spectrum of Li[**7**] (125.8 MHz, THF-d_8).

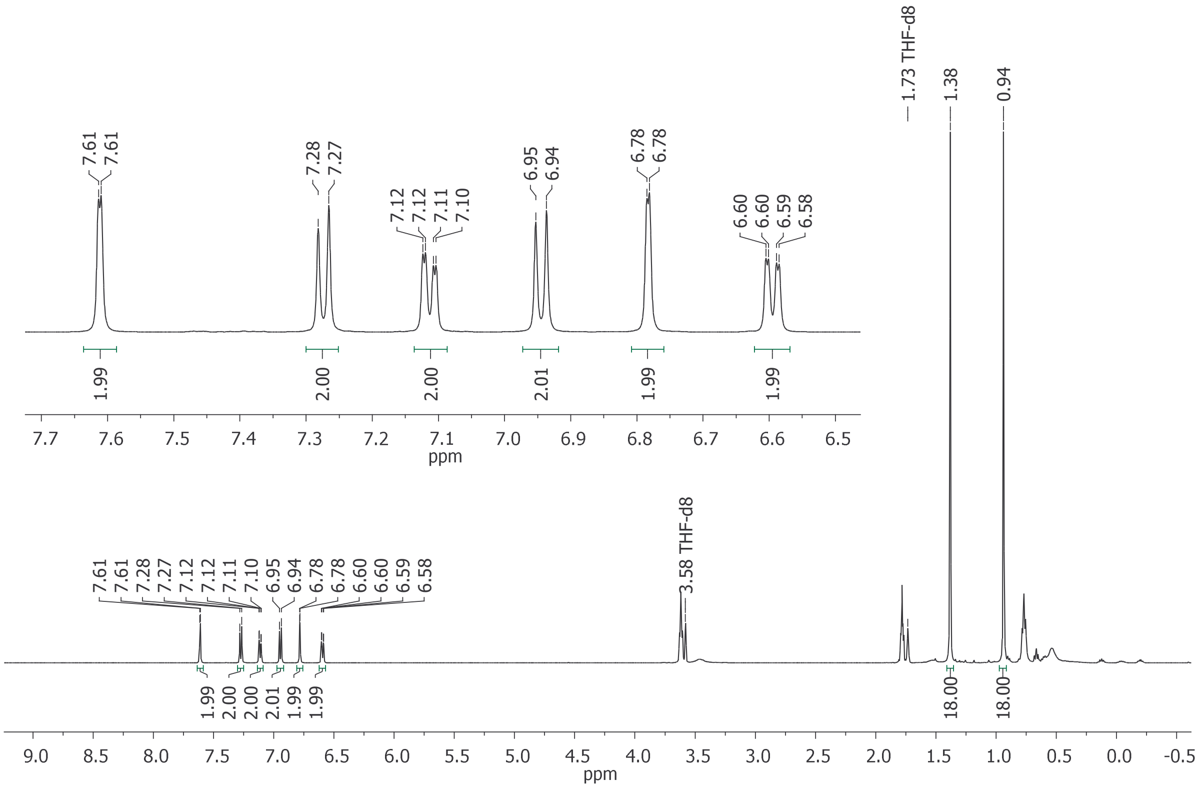

Figure S23: ^{1}H NMR spectrum of Li[**10**] (500.2 MHz, THF-d_8).

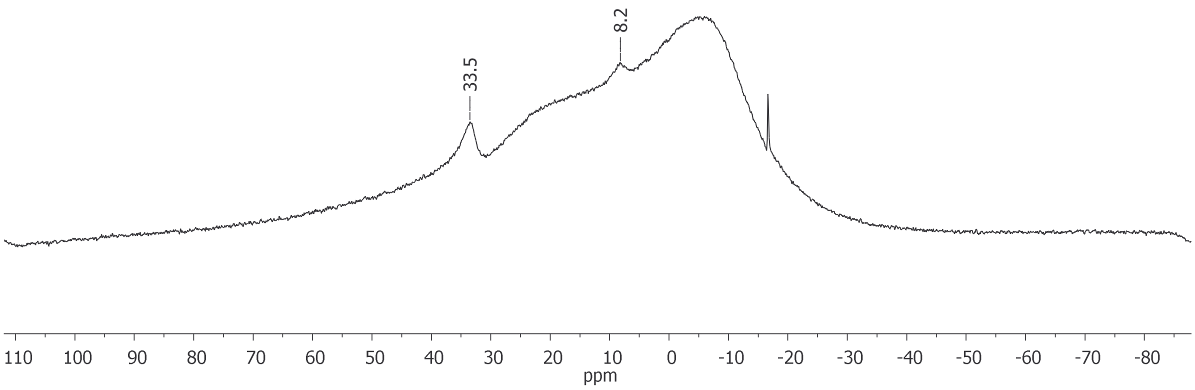

Figure S24: ^{11}B NMR spectrum of Li[**10**] (96.3 MHz, THF-d_8).

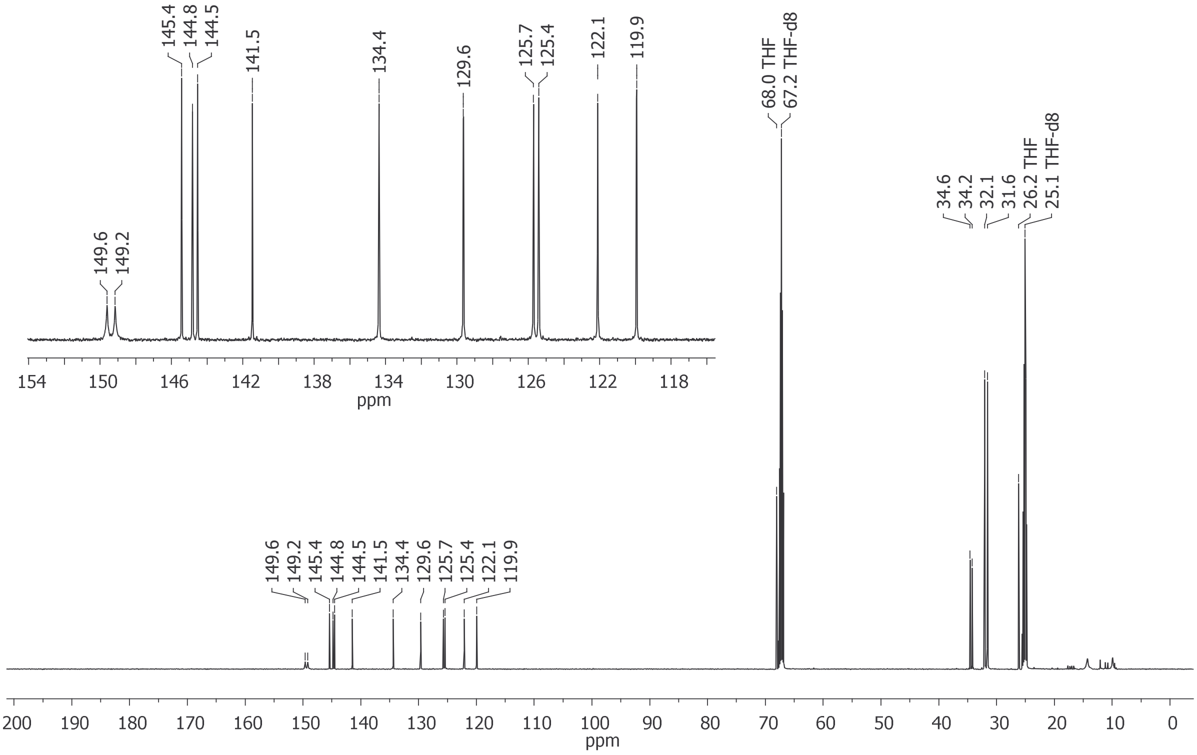

Figure S25: $^{13}C\{^1H\}$ NMR spectrum of Li[**10**] (125.8 MHz, THF-d_8).

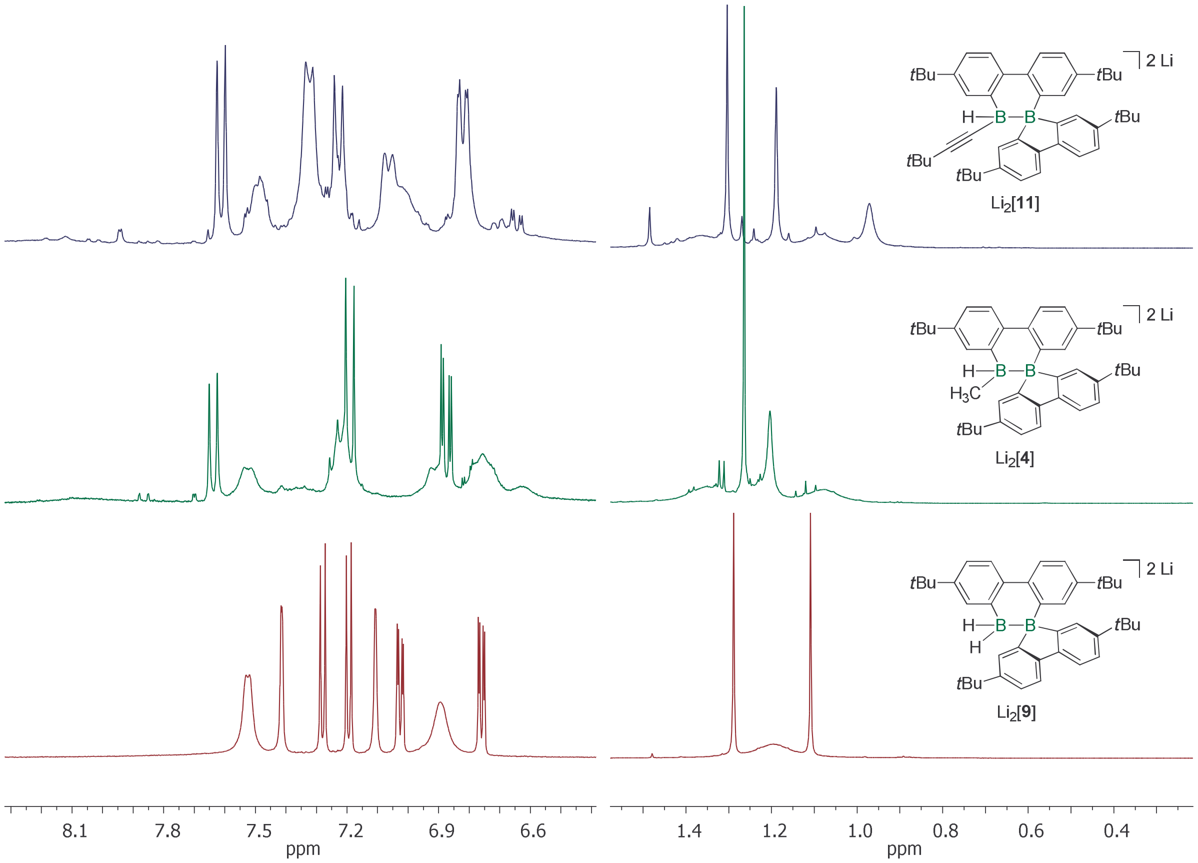

Figure S26: ^{1}H NMR spectra of Li_2[**11**] (top; 300.0 MHz), Li_2[**4**] (middle; 300.0 MHz), and Li_2[**9**] (bottom; 500.2 MHz) in THF-d_8. Aryl and alkyl regions are scaled differently. In the case of Li_2[**9**], the poor resolution of some signals in the aromatic and aliphatic spectral region originates from a dynamic behavior of the system in solution, which arises from conformational changes of the twisted B_2C_4 ring and/or from an association-dissociation equilibrium between the cations and the anions.[S5] In the cases of Li_2[**4**] and Li_2[**11**], the phenomenon is even more pronounced due to further symmetry breaking by the boron-bonded organyl substituents.

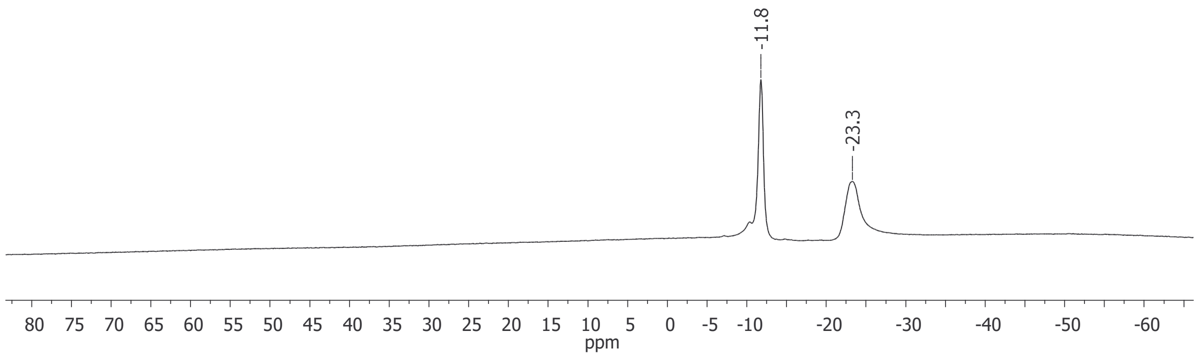

Figure S27: ^{11}B NMR spectrum of Li_2[**11**] (96.3 MHz, THF-d_8).

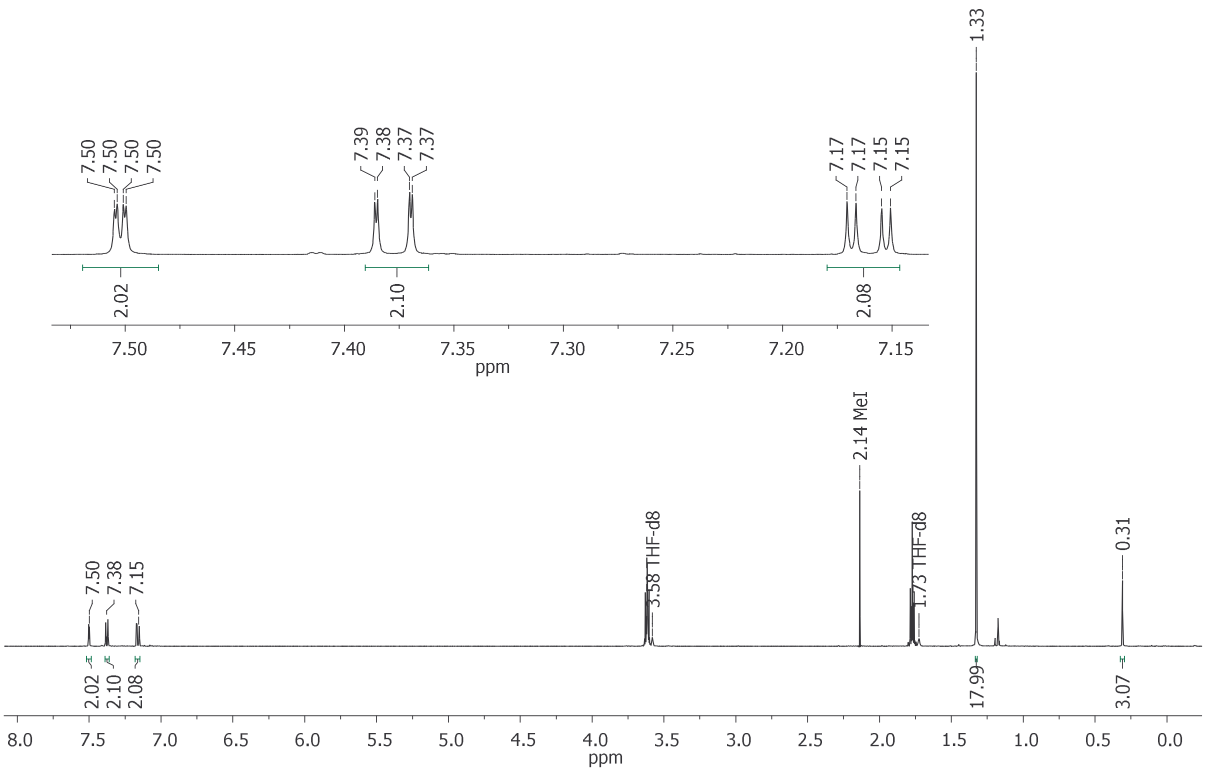

Figure S28: ^{1}H NMR spectrum of **13** (500.2 MHz, THF-d_8).

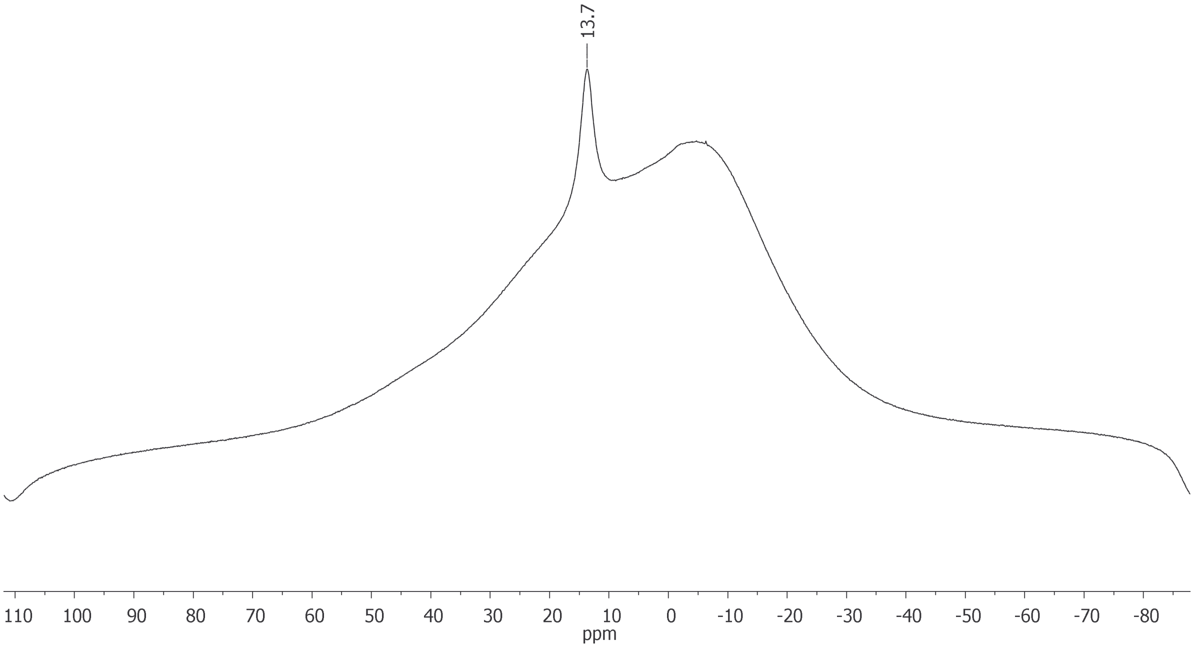

Figure S29: ^{11}B NMR spectrum of **13** (160.5 MHz, THF-d_8).

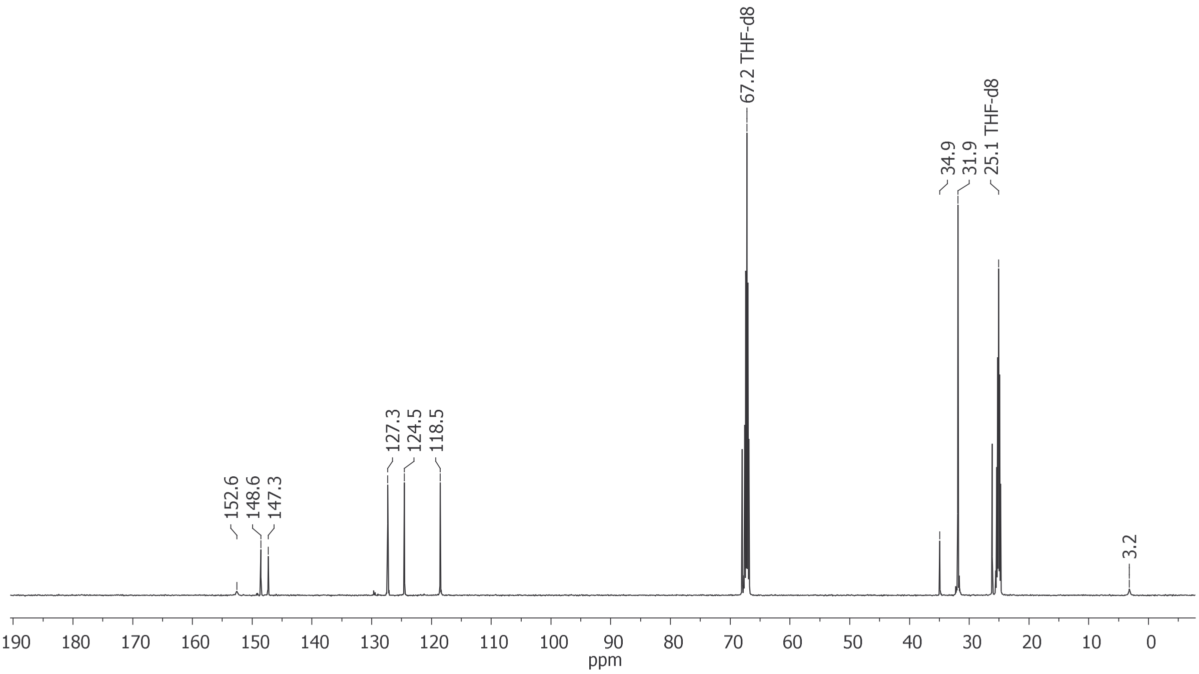

Figure S30: $^{13}C\{^1H\}$ NMR spectrum of **13** (125.8 MHz, THF-d_8).

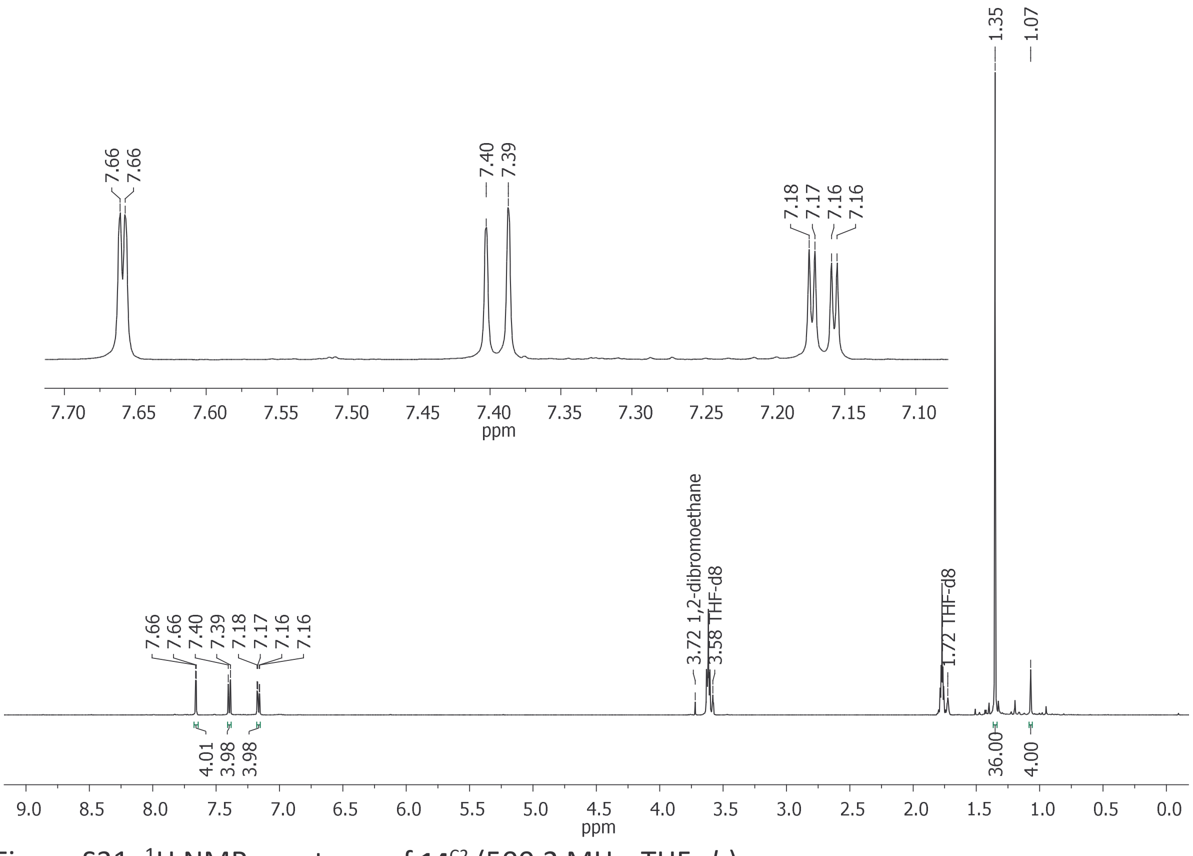

Figure S31: ^1H NMR spectrum of $\mathbf{14}^{C2}$ (500.2 MHz, THF-d_8).

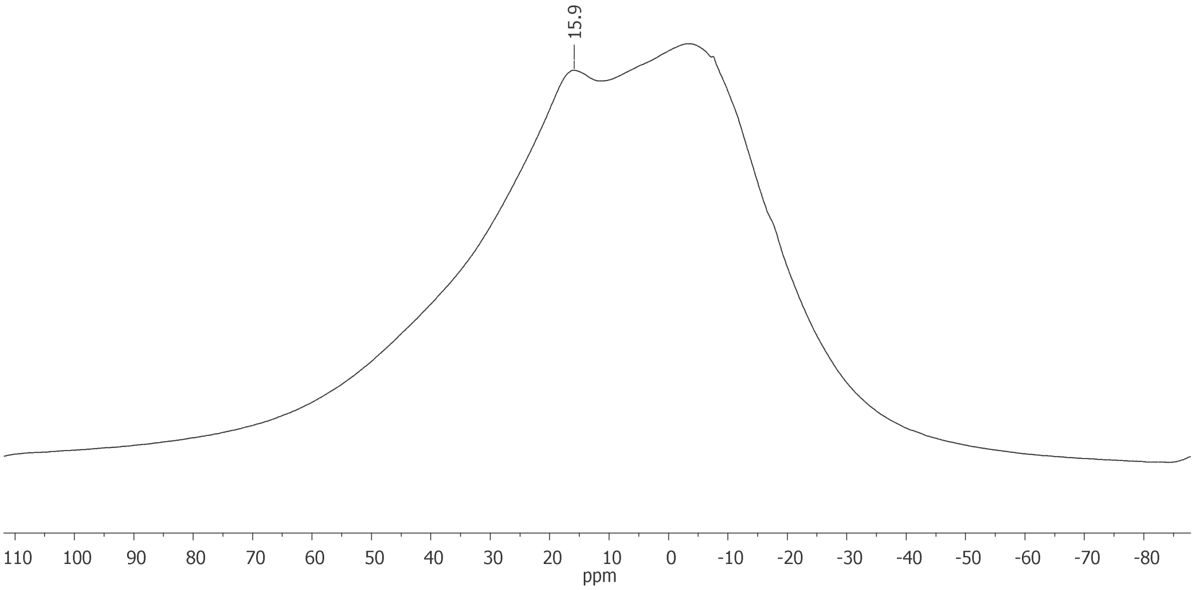

Figure S32: ^{11}B NMR spectrum of **14**^{C2} (160.5 MHz, THF-d_8).

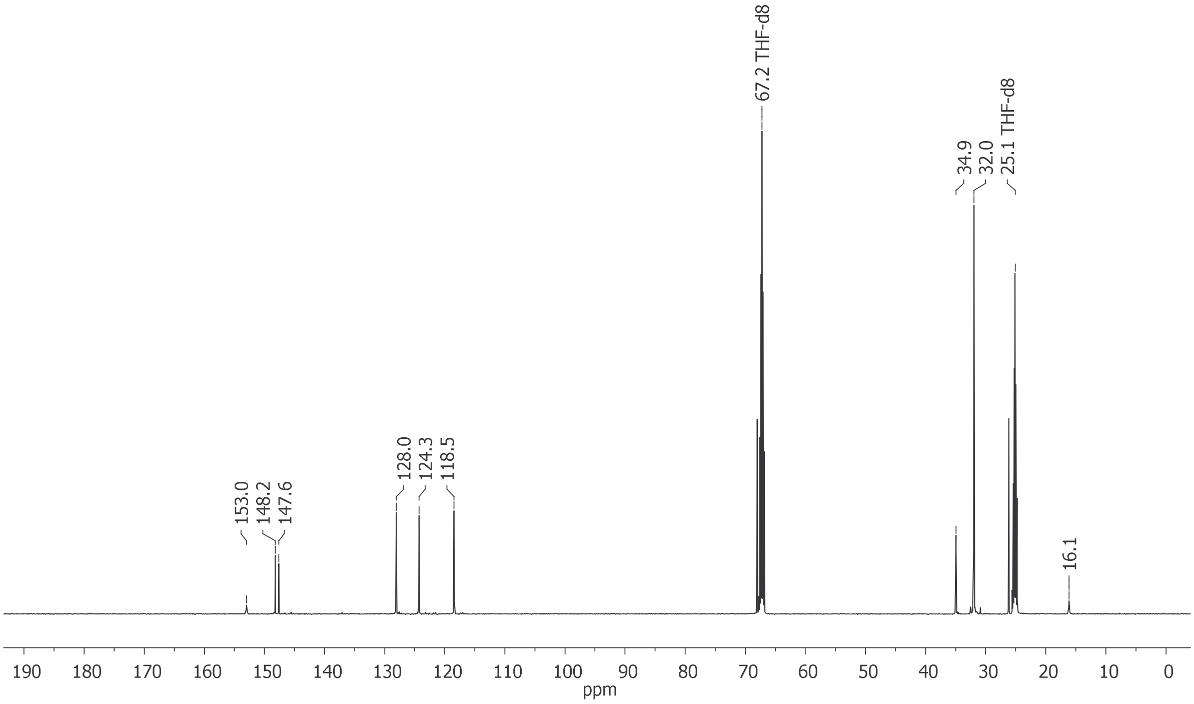

Figure S33: $^{13}C\{^{1}H\}$ NMR spectrum of **14**^{C2} (125.8 MHz, THF-d_8).

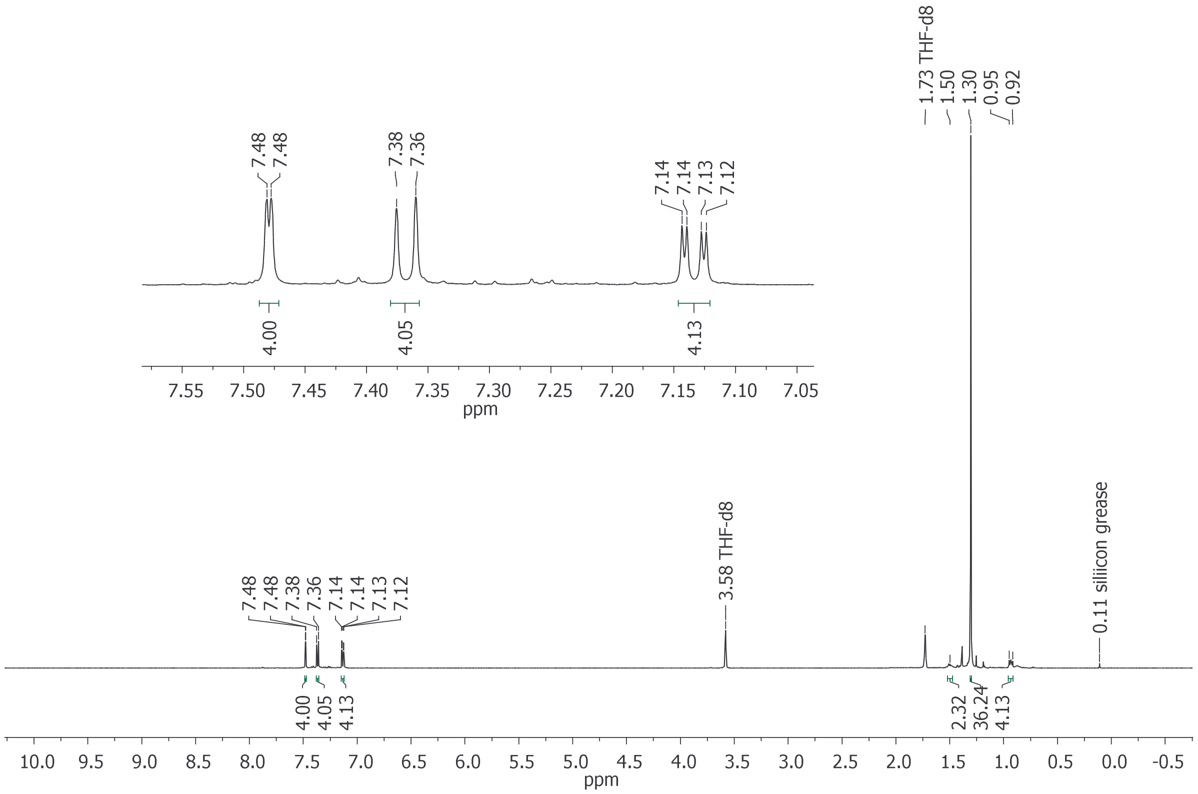

Figure S34: ^{1}H NMR spectrum of **14**^{C3} (500.2 MHz, THF-d_8).

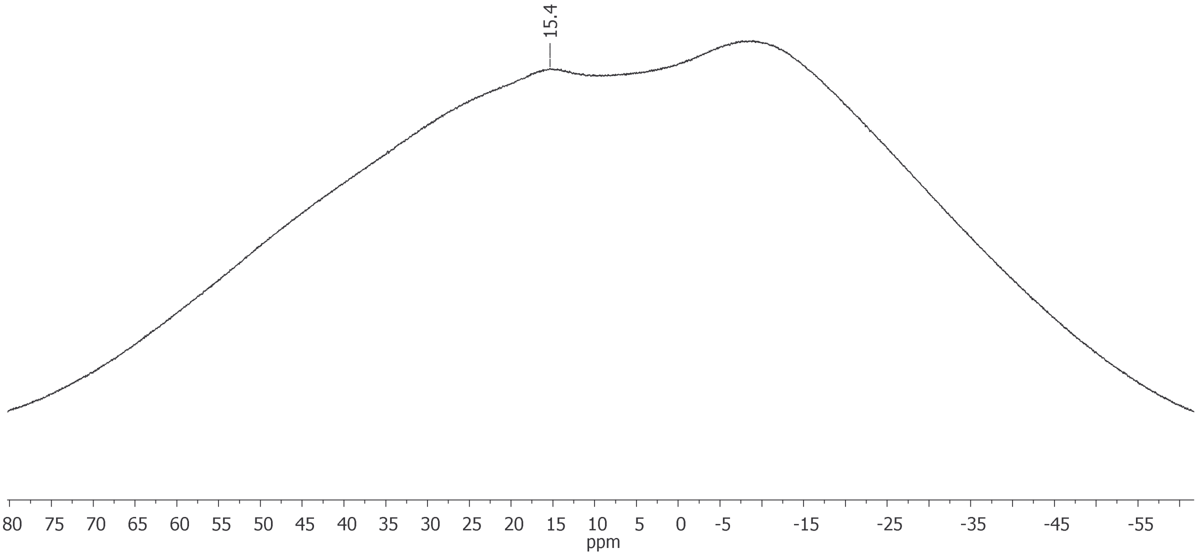

Figure S35: ^{11}B NMR spectrum of **14**^{C3} (160.5 MHz, THF-d_8).

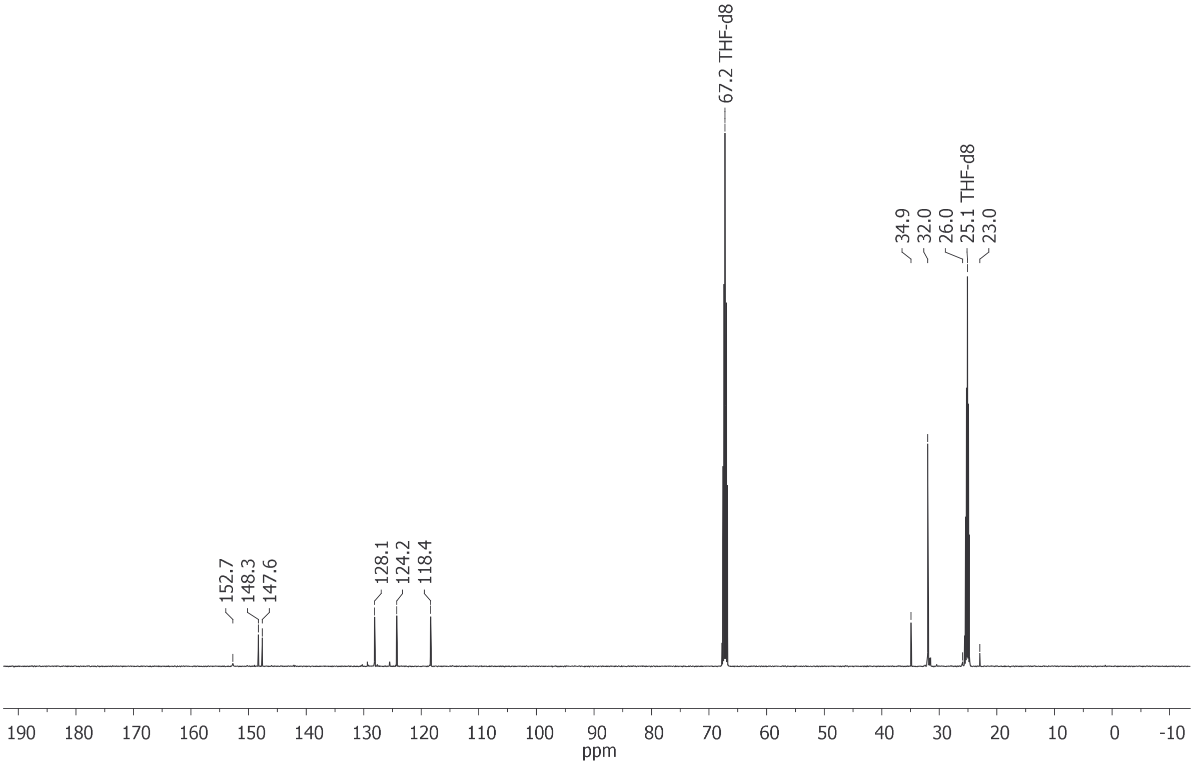

Figure S36: $^{13}C\{^{1}H\}$ NMR spectrum of **14**^{C3} (125.8 MHz, THF-d_8).

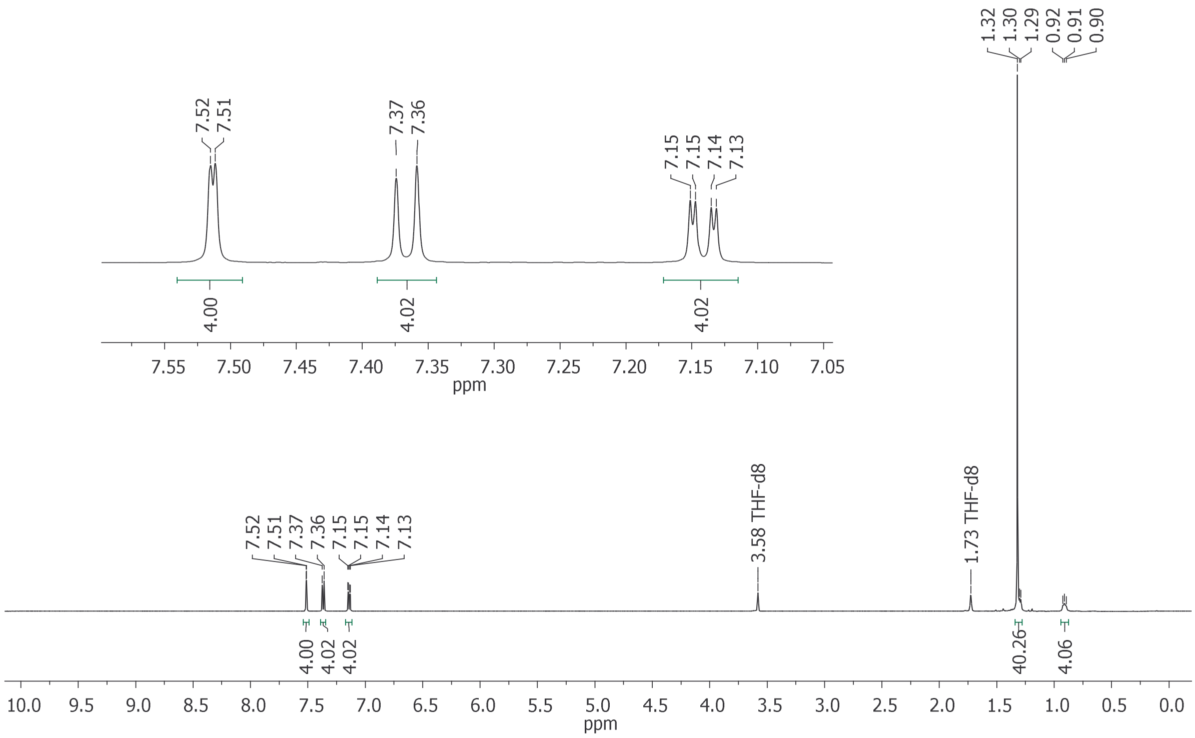

Figure S37: ^{1}H NMR spectrum of **14**^{C4} (500.2 MHz, THF-d_8).

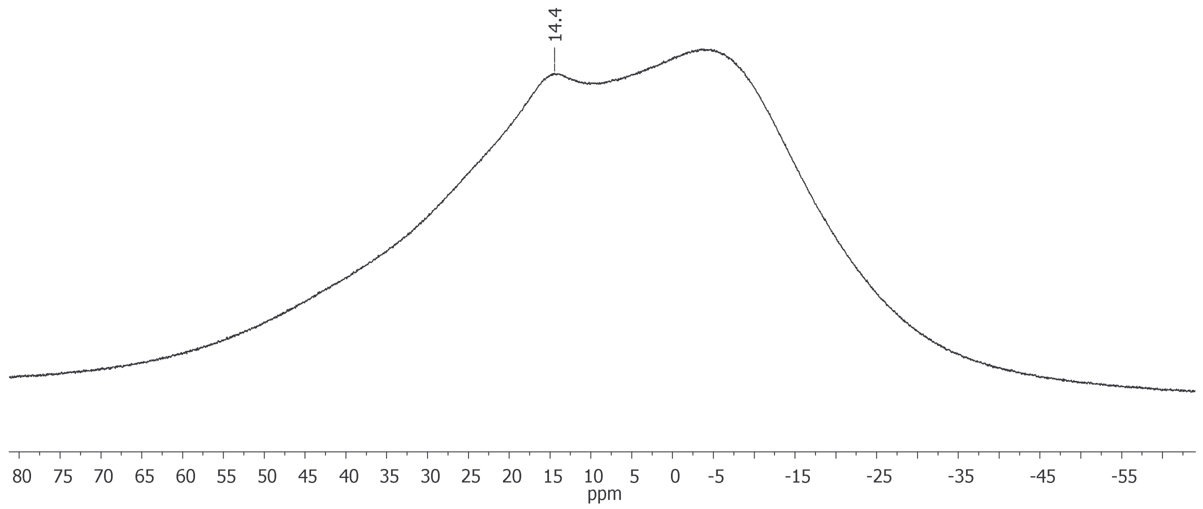

Figure S38: ^{11}B NMR spectrum of **14**^{C4} (160.5 MHz, THF-d_8).

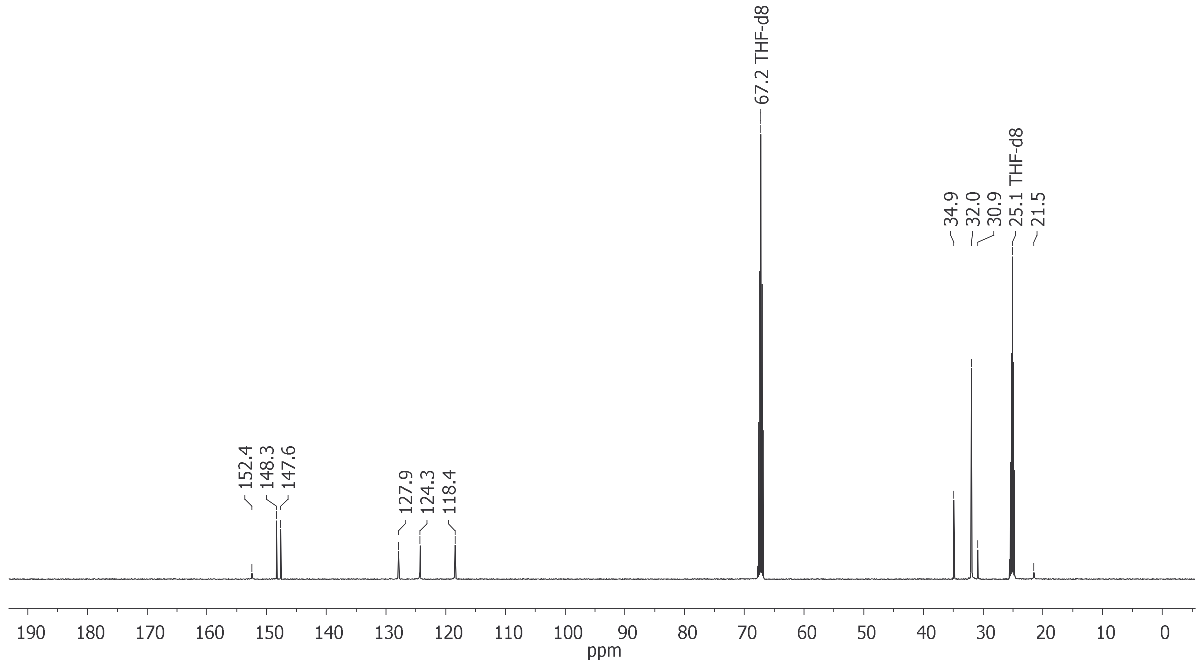

Figure S39: ^{13}C{^{1}H} NMR spectrum of **14**^{C4} (125.8 MHz, THF-d_8).

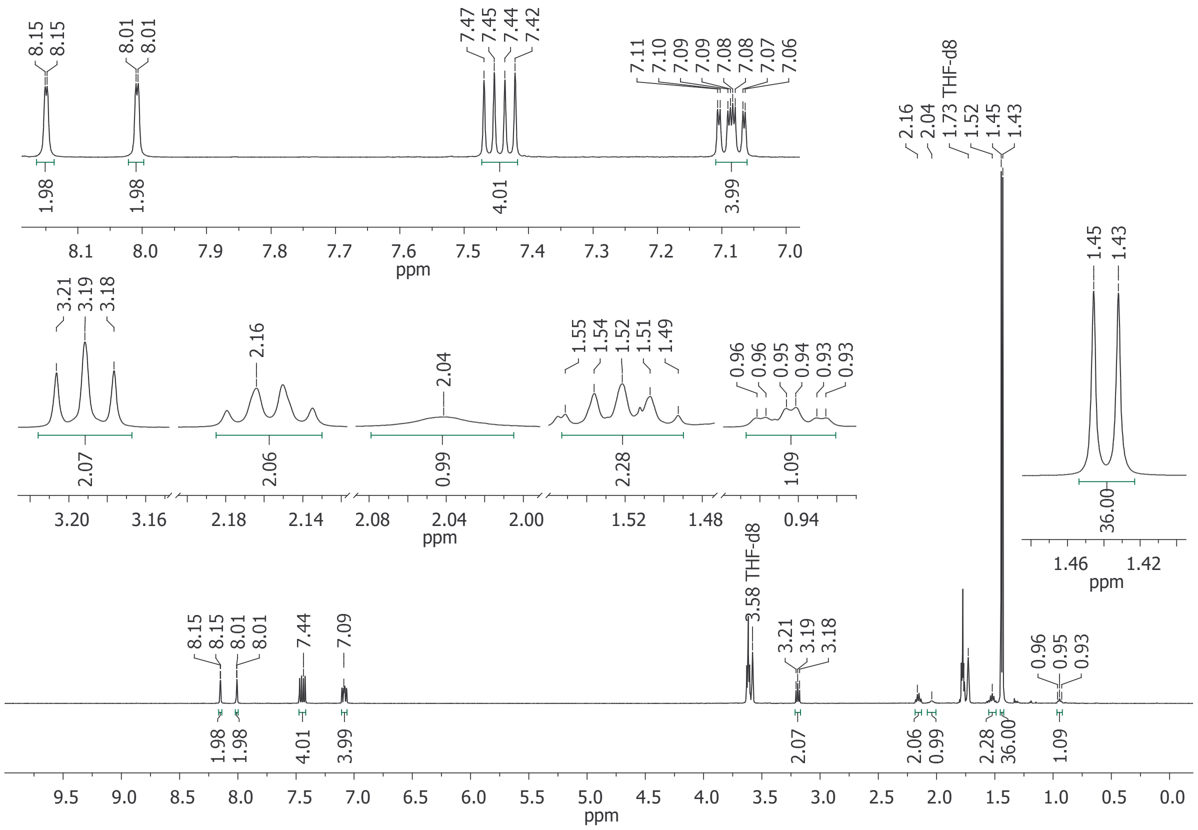

Figure S40: ^{1}H NMR spectrum of Li[**15**^{C4,Cl}] (500.2 MHz, THF-d_8).

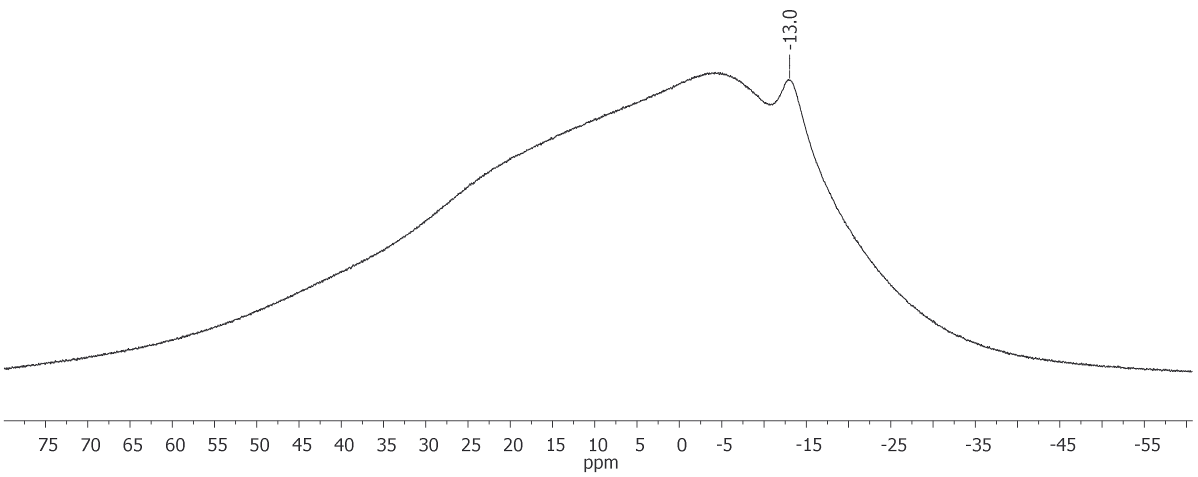

Figure S41: ^{11}B NMR spectrum of Li[**15**^{C4,Cl}] (160.5 MHz, THF-d_8).

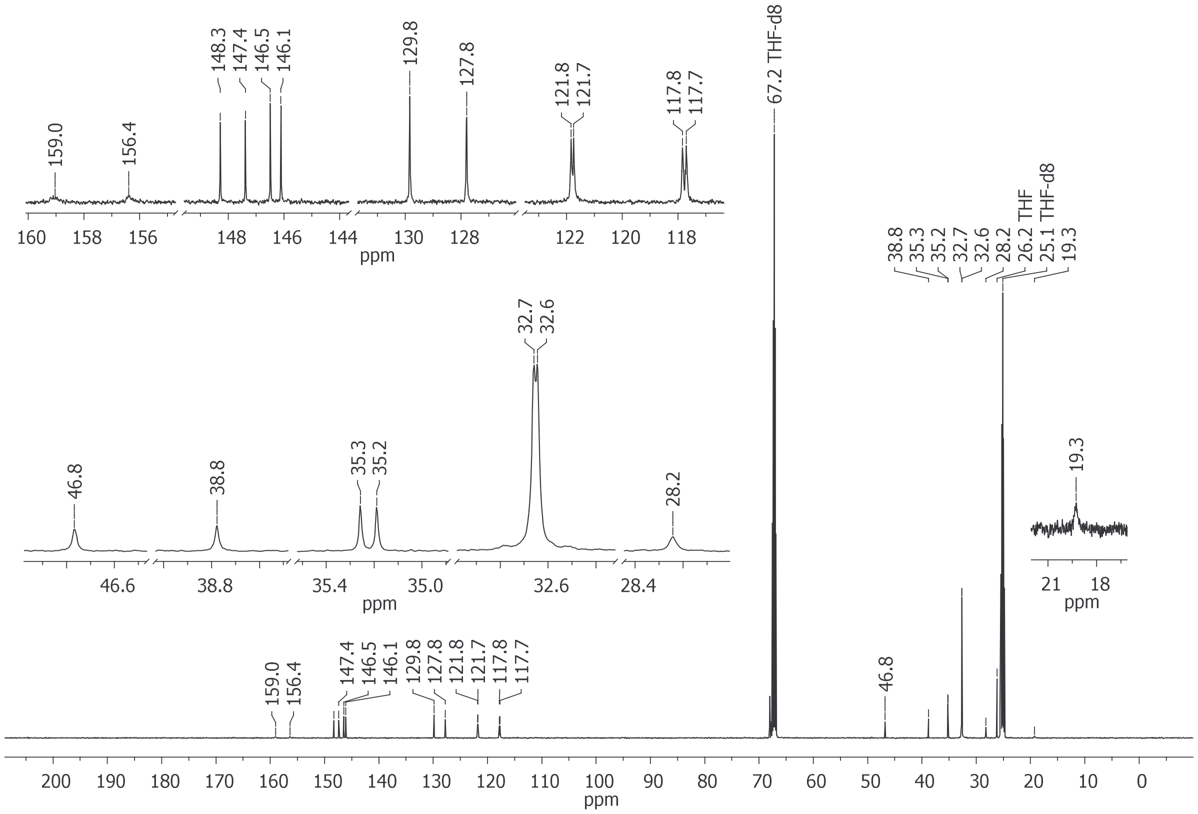

Figure S42: $^{13}C\{^{1}H\}$ NMR spectrum of Li[**15**^{C4,Cl}] (125.8 MHz, THF-d_8).

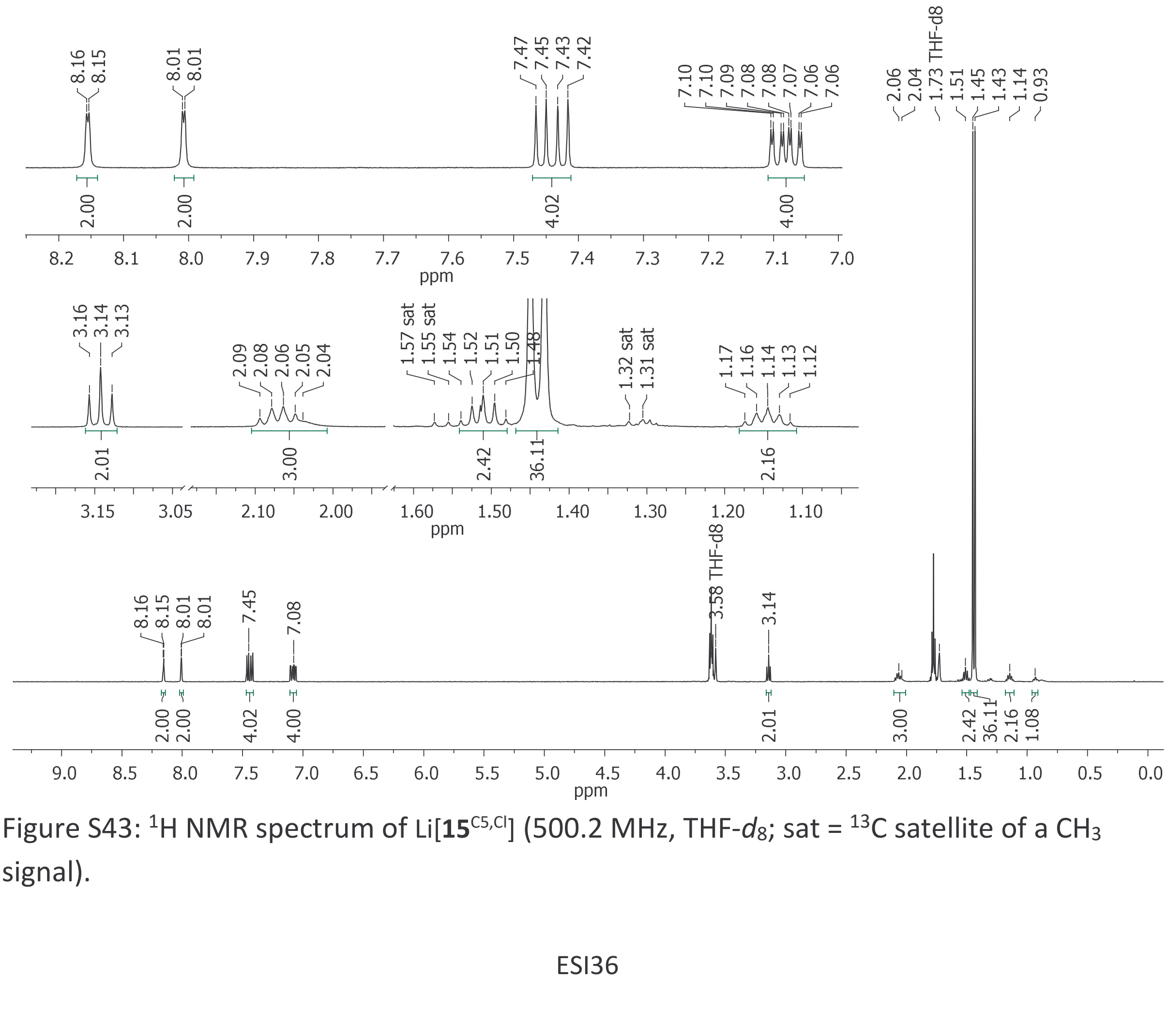

Figure S43: ^{1}H NMR spectrum of Li[**15**^{C5,Cl}] (500.2 MHz, THF-d_8; sat = ^{13}C satellite of a CH_3 signal).

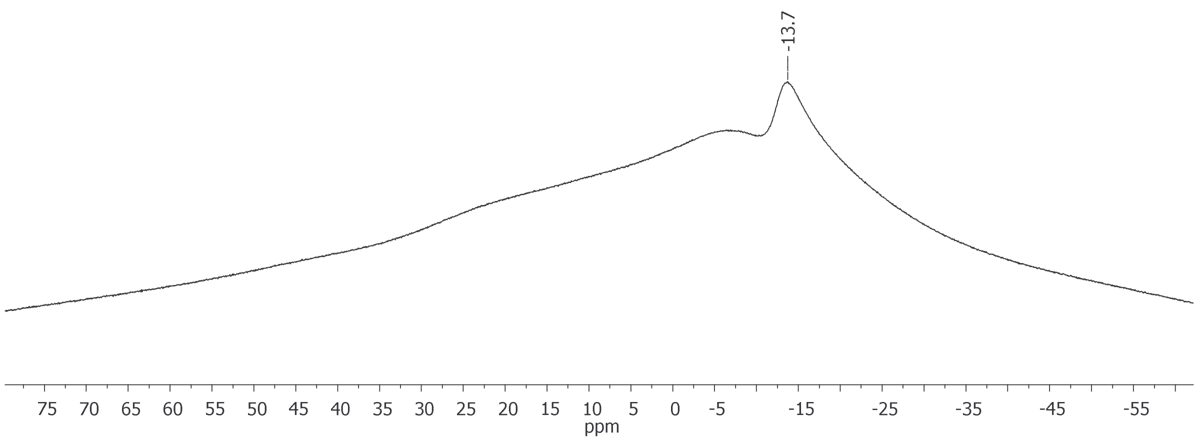

Figure S44: ^{11}B NMR spectrum of Li[**15**^{C5,Cl}] (160.5 MHz, THF-d_8).

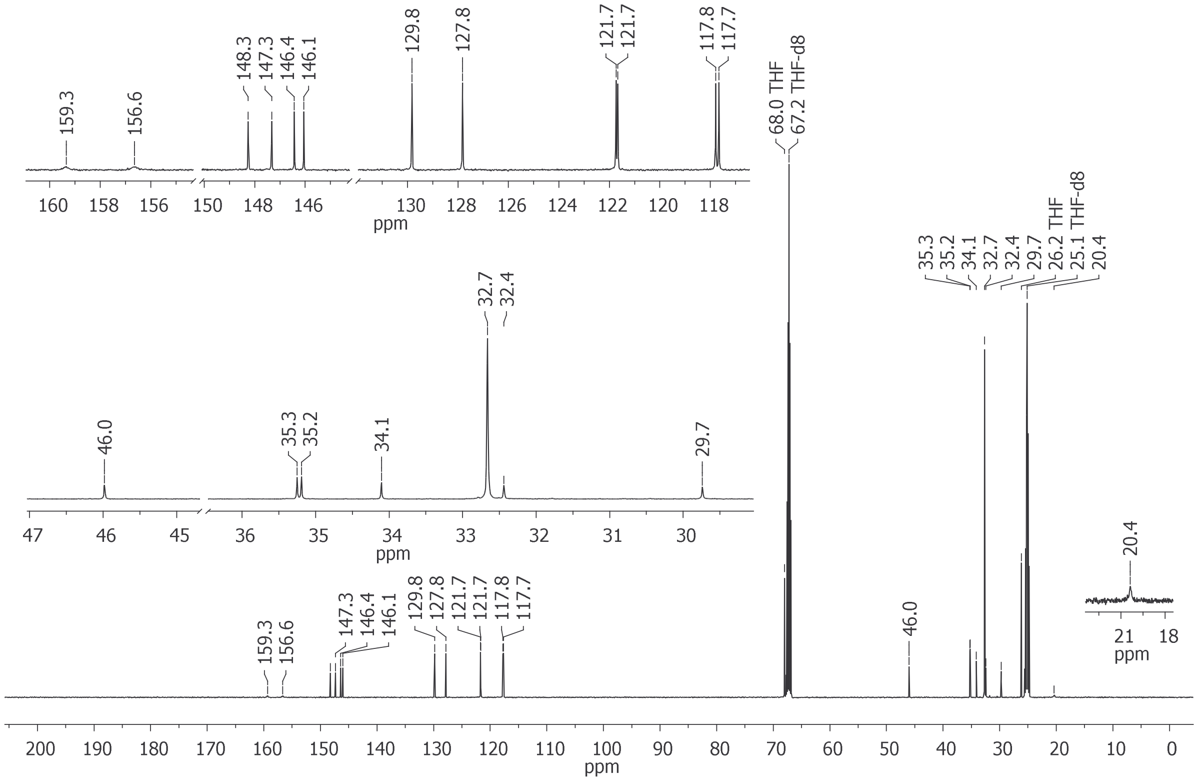

Figure S45: ^{13}C{^{1}H} NMR spectrum of Li[**15**^{C5,Cl}] (125.8 MHz, THF-d_8).

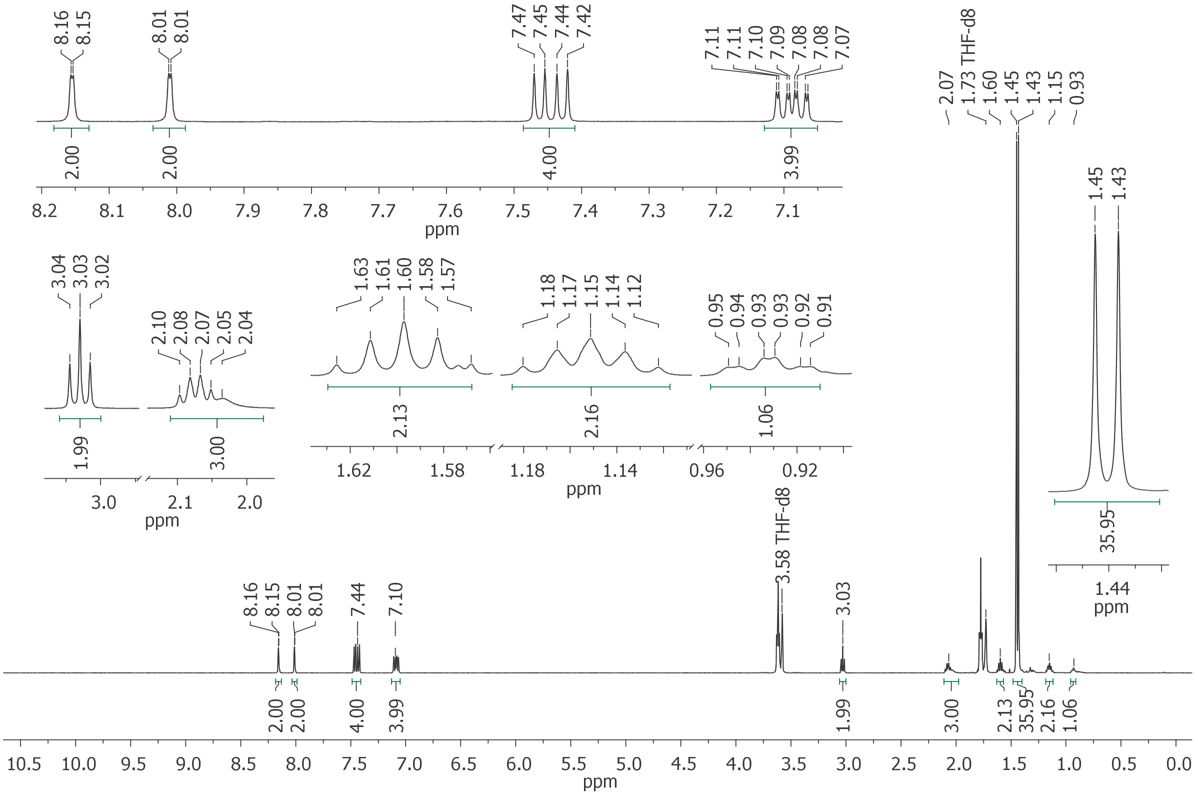

Figure S46: ^{1}H NMR spectrum of Li[**15**^{C5,Br}] (500.2 MHz, THF-d_8).

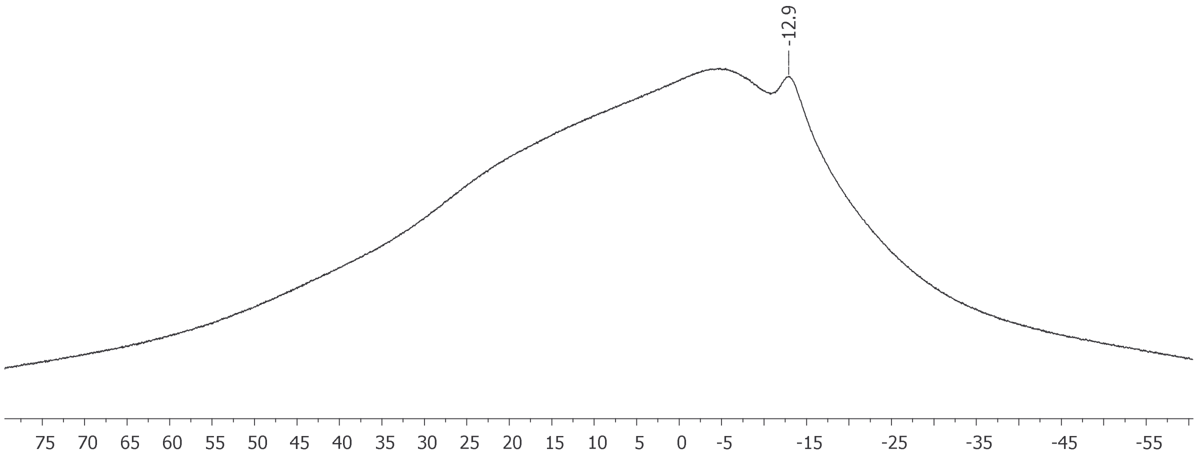

Figure S47: ^{11}B NMR spectrum of Li[**15**^{C5,Br}] (160.5 MHz, THF-d_8).

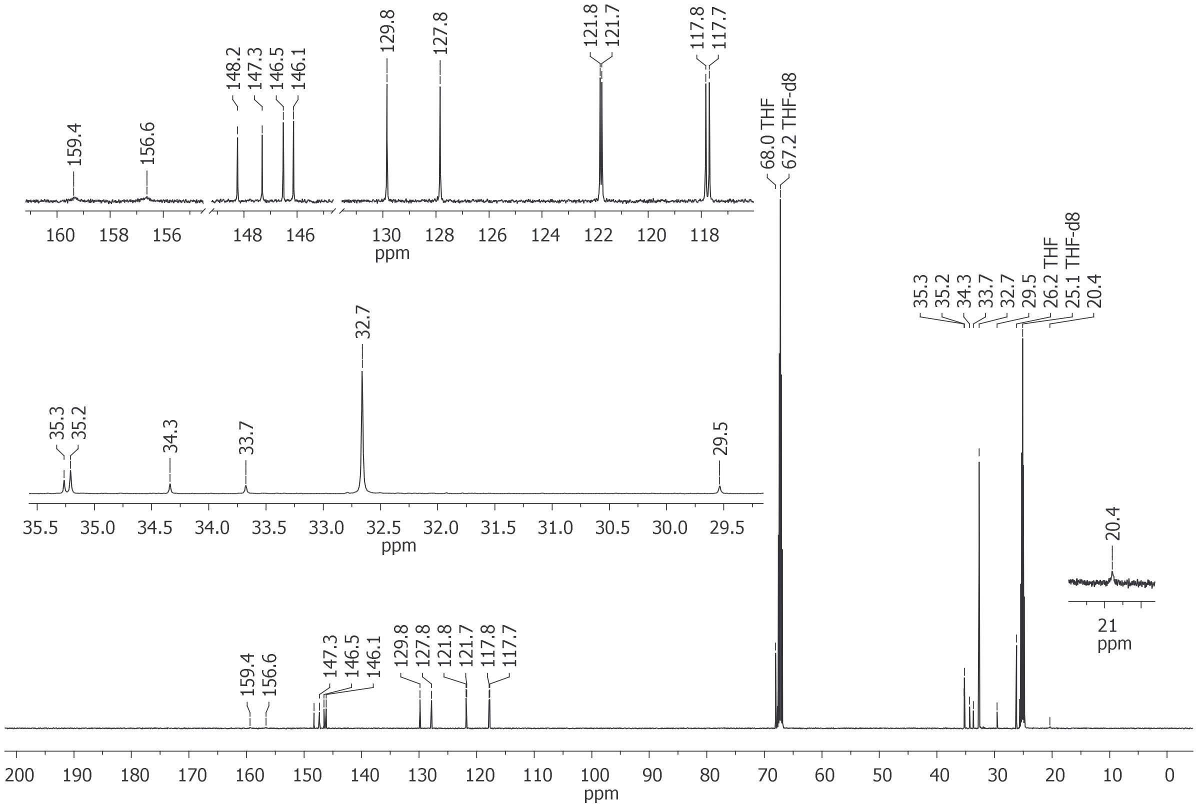

Figure S48: $^{13}C\{^{1}H\}$ NMR spectrum of Li[**15**^{C5,Br}] (125.8 MHz, THF-d_8).

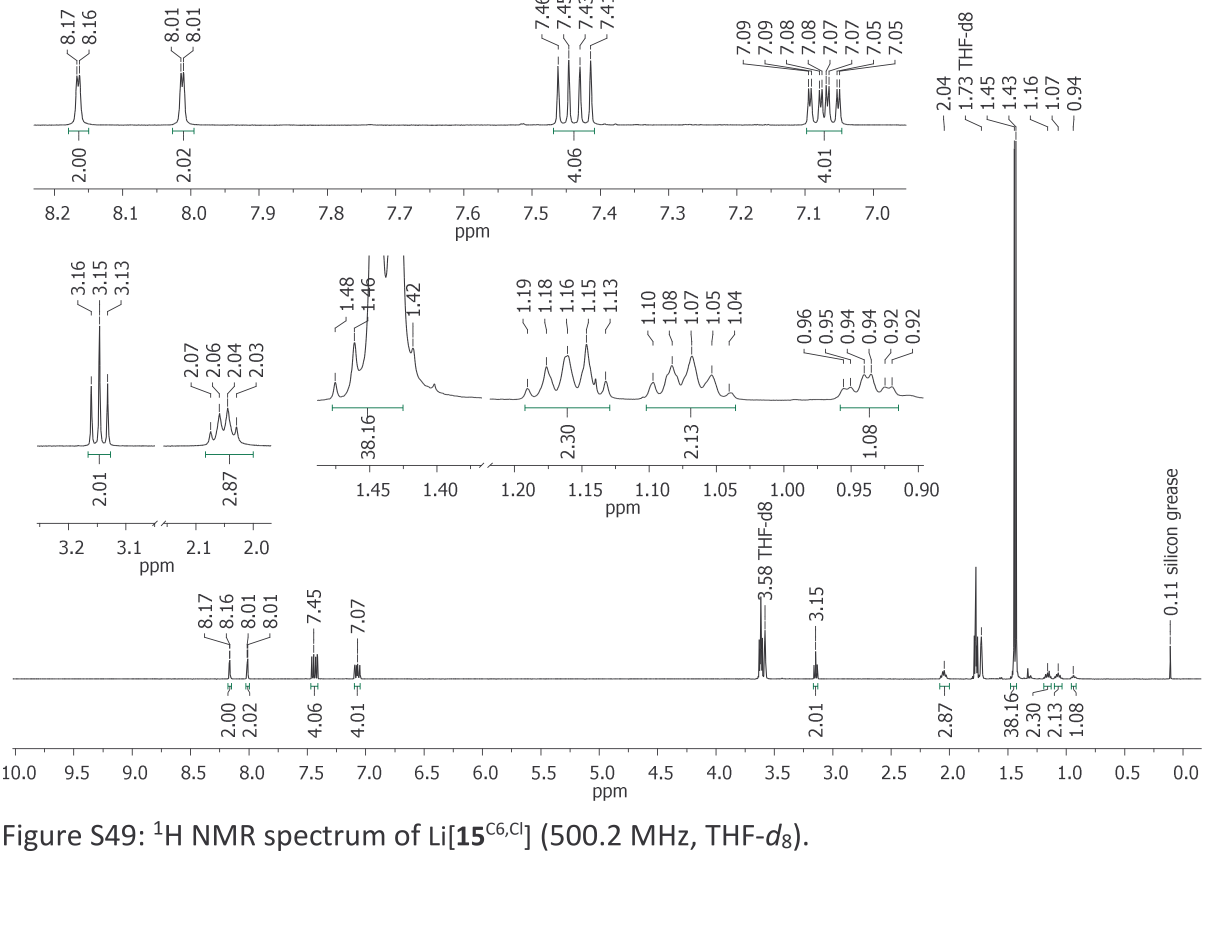

Figure S49: ^{1}H NMR spectrum of Li[**15**^{C6,Cl}] (500.2 MHz, THF-d_8).

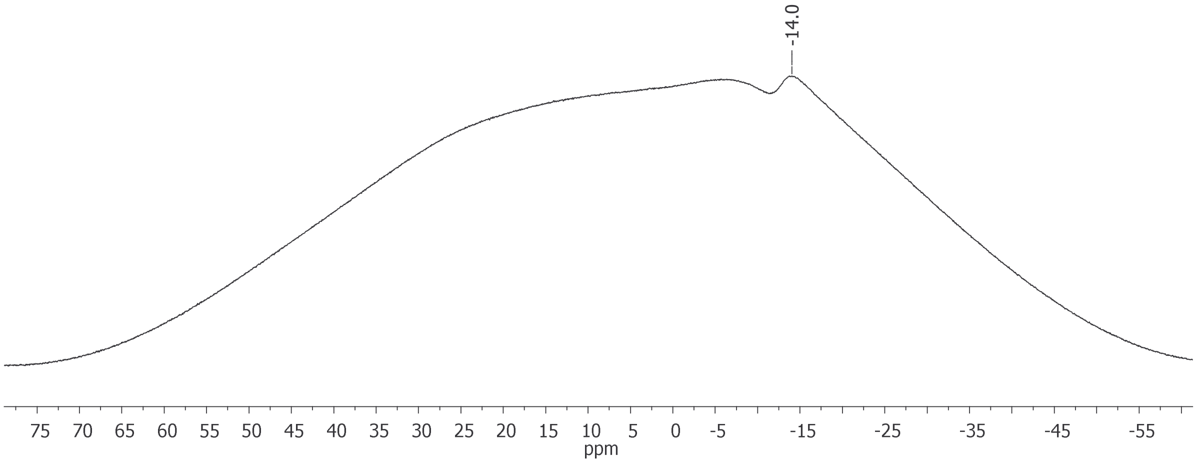

Figure S50: ^{11}B NMR spectrum of Li[**15**^{C6,Cl}] (160.5 MHz, THF-d_8,).

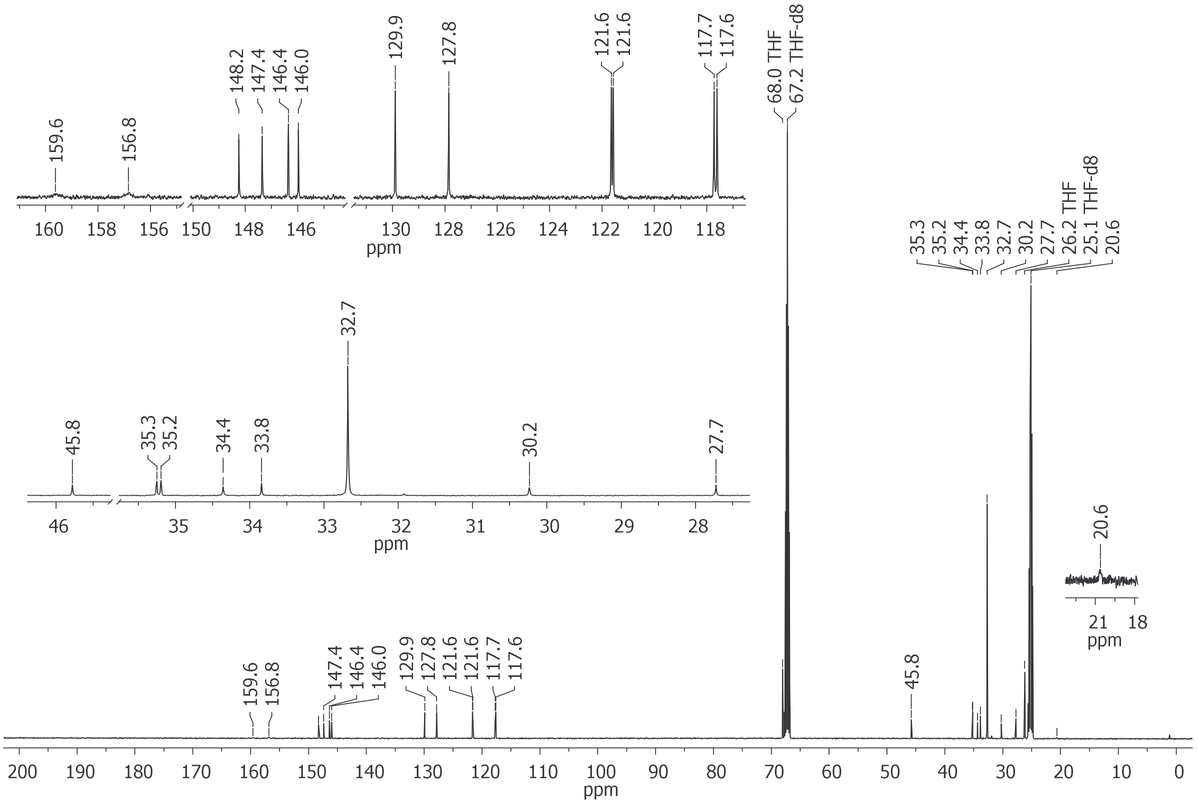

Figure S51: ^{13}C{^{1}H} NMR spectrum of Li[**15**^{C6,Cl}] (125.8 MHz, THF-d_8,).

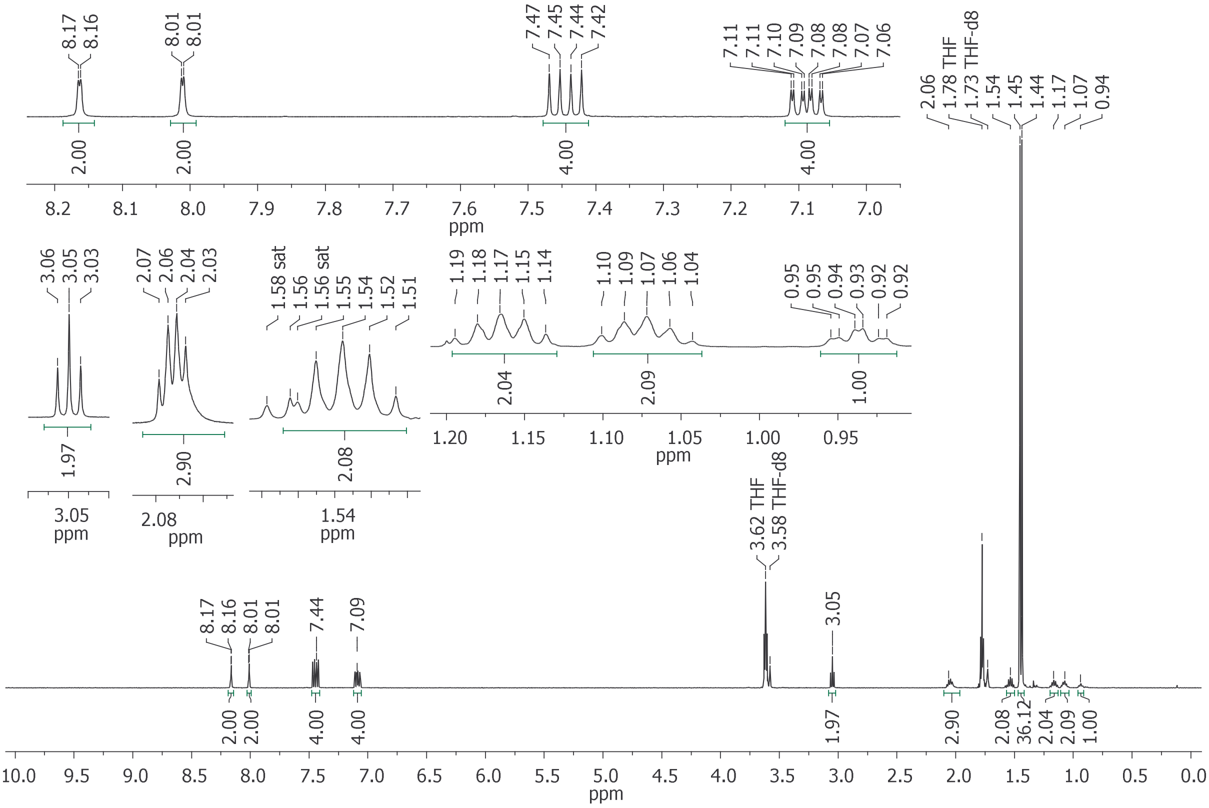

Figure S52: ^{1}H NMR spectrum of Li[**15**^{C6,Br}] (500.2 MHz, THF-d_8; sat = ^{13}C satellite of a CH_3 signal).

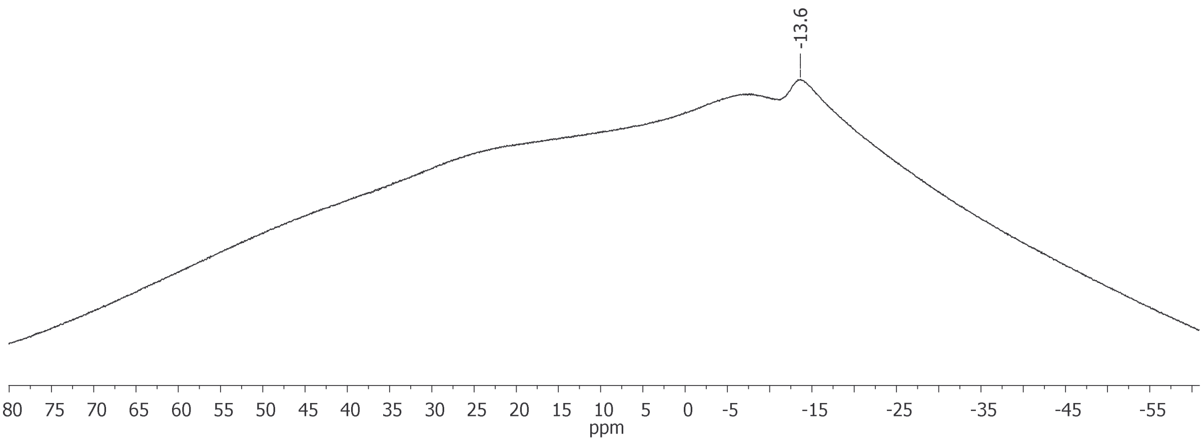

Figure S53: ^{11}B NMR spectrum of Li[**15**^{C6,Br}] (160.5 MHz, THF-d_8).

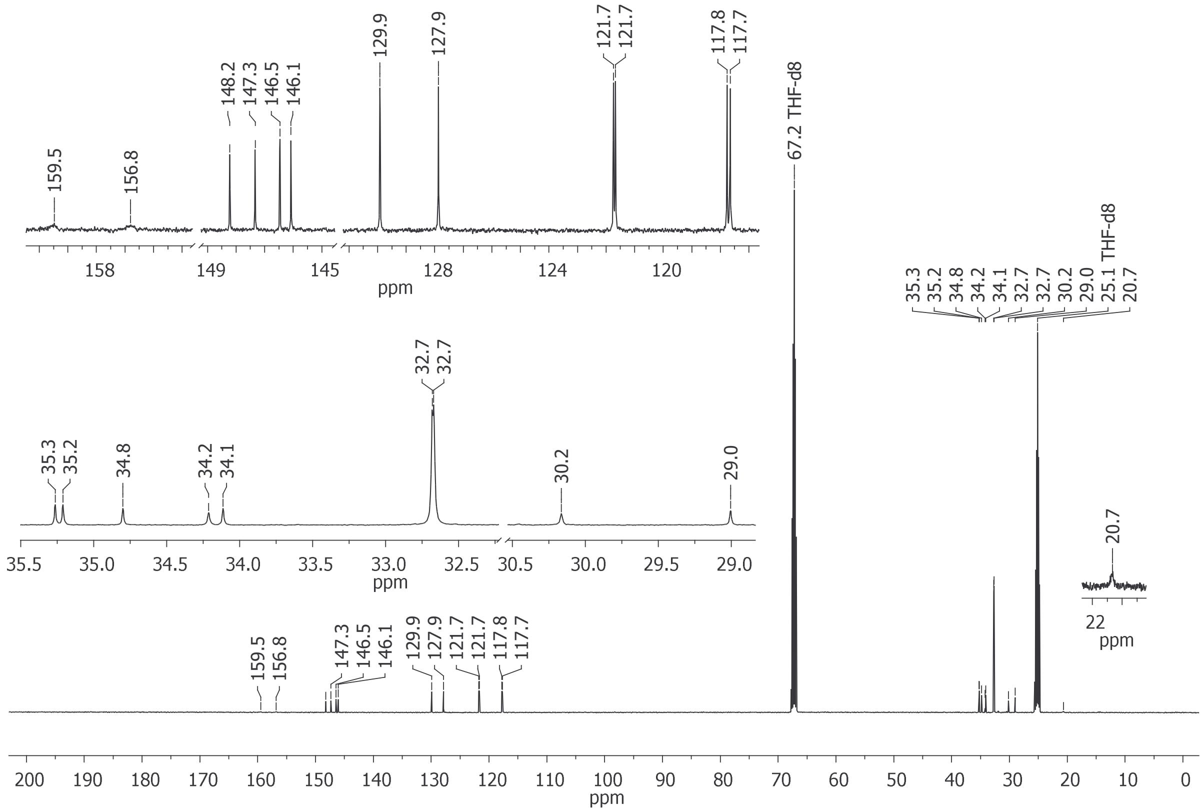

Figure S54: $^{13}C\{^1H\}$ NMR spectrum of Li[**15**^{C6,Br}] (125.8 MHz, THF-d_8).

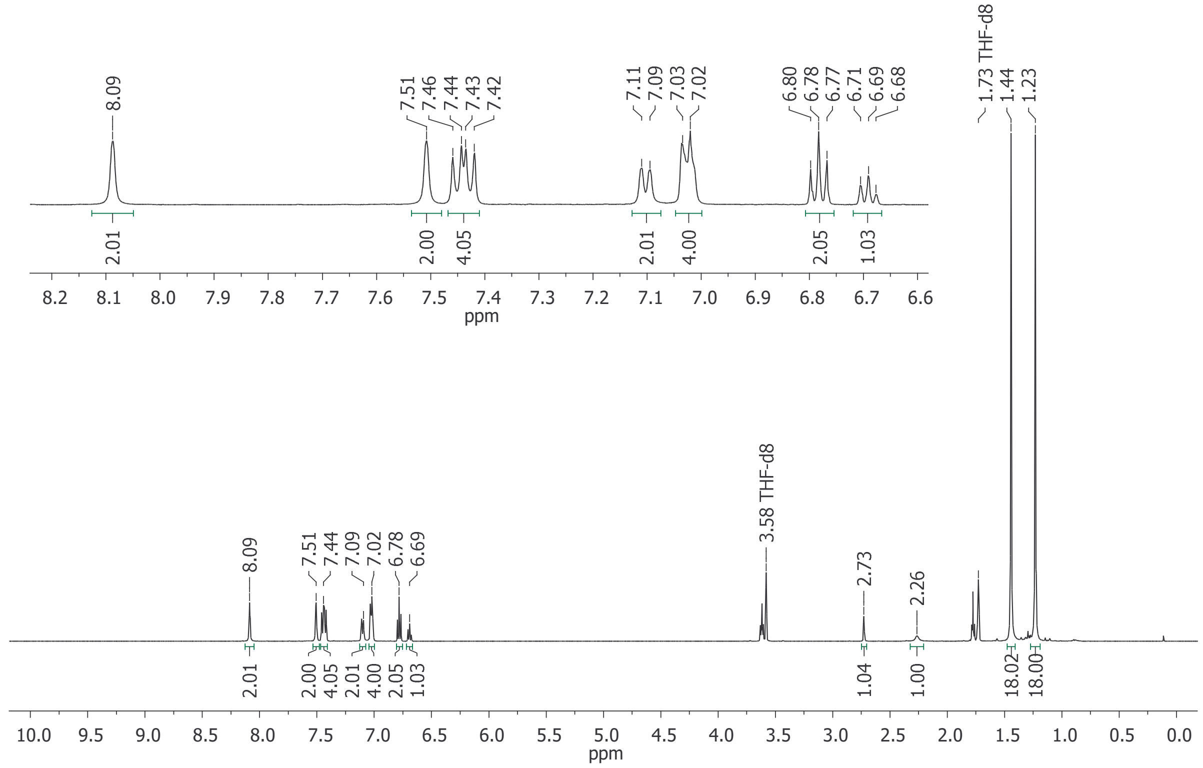

Figure S55: ^1H NMR spectrum of Li[**16**] (500.2 MHz, THF-d_8).

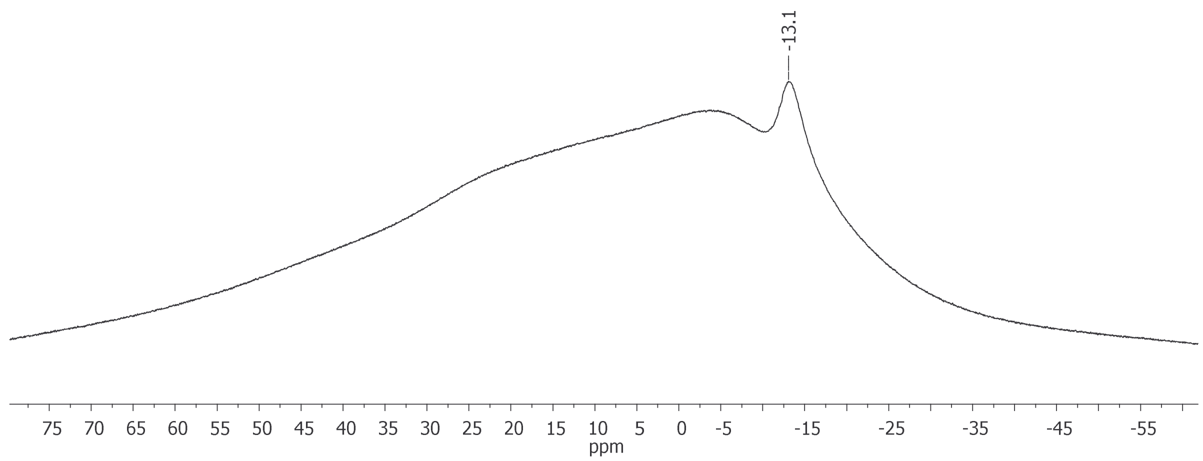

Figure S56: ^{11}B NMR spectrum of Li[**16**] (160.5 MHz, THF-d_8).

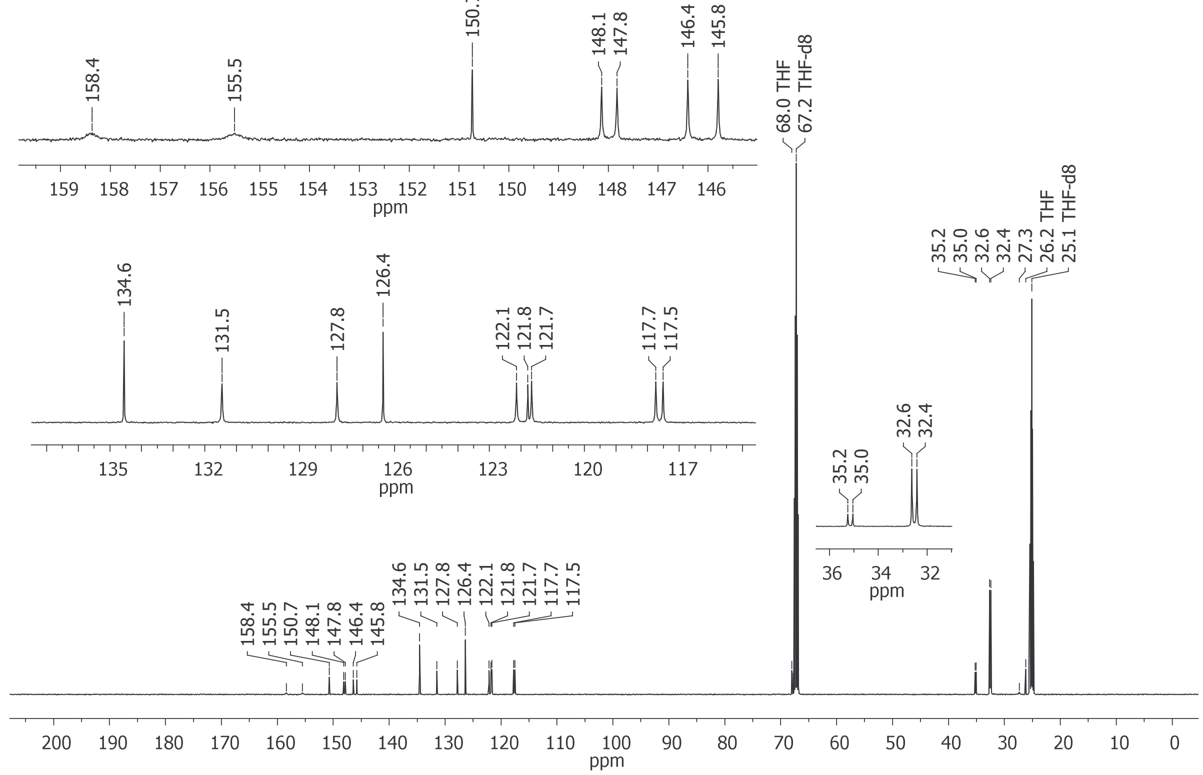

Figure S57: ^{13}C{^{1}H} NMR spectrum of Li[**16**] (125.8 MHz, THF-d_8).

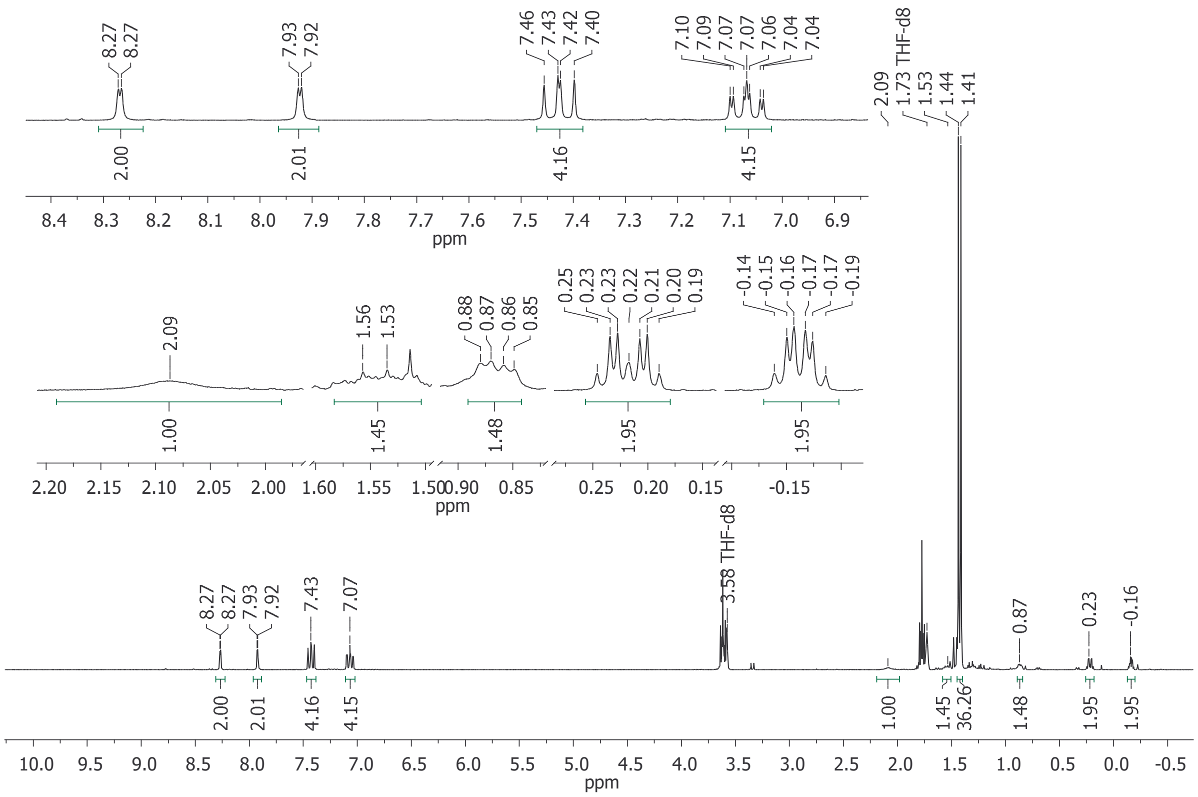

Figure S58: ^{1}H NMR spectrum of Li[**17**] (300.0 MHz, THF-d_8).

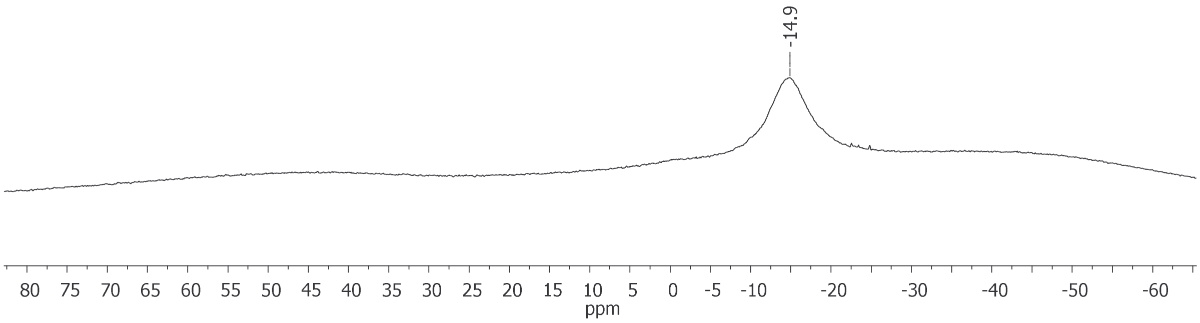

Figure S59: ^{11}B NMR spectrum of Li[**17**] (96.3 MHz, THF-d_8).

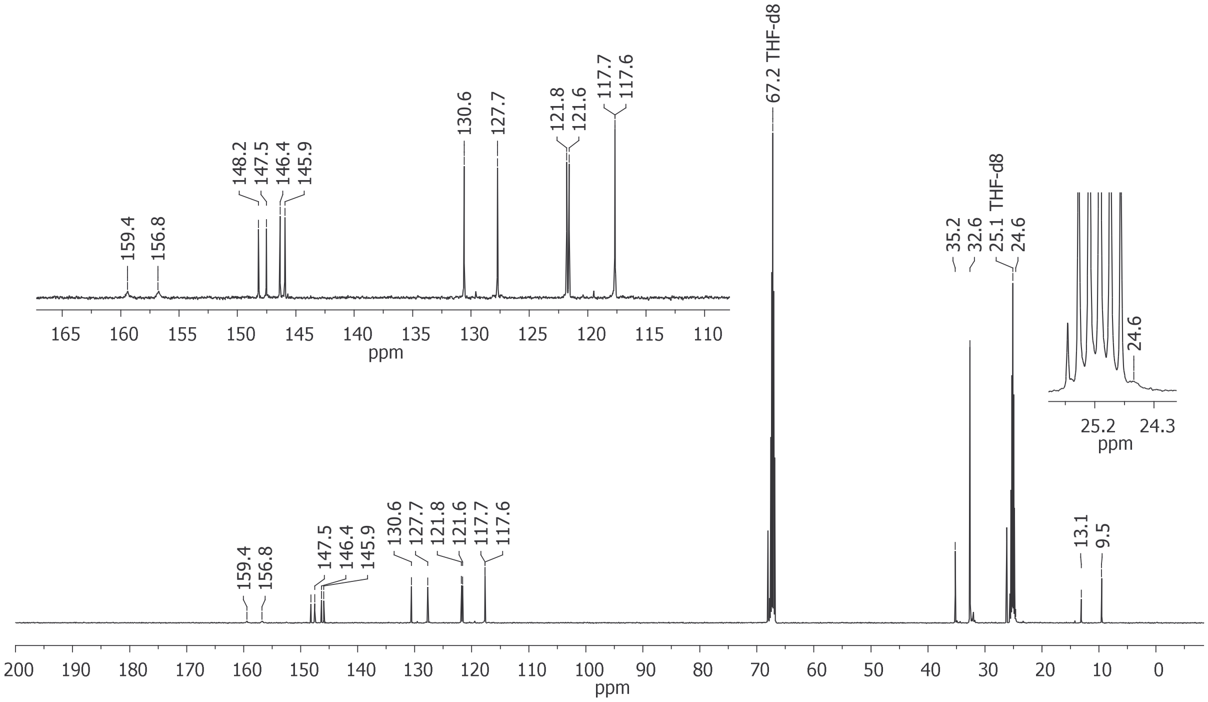

Figure S60: $^{13}C\{^{1}H\}$ NMR spectrum of Li[**17**] (125.8 MHz, THF-d_8).

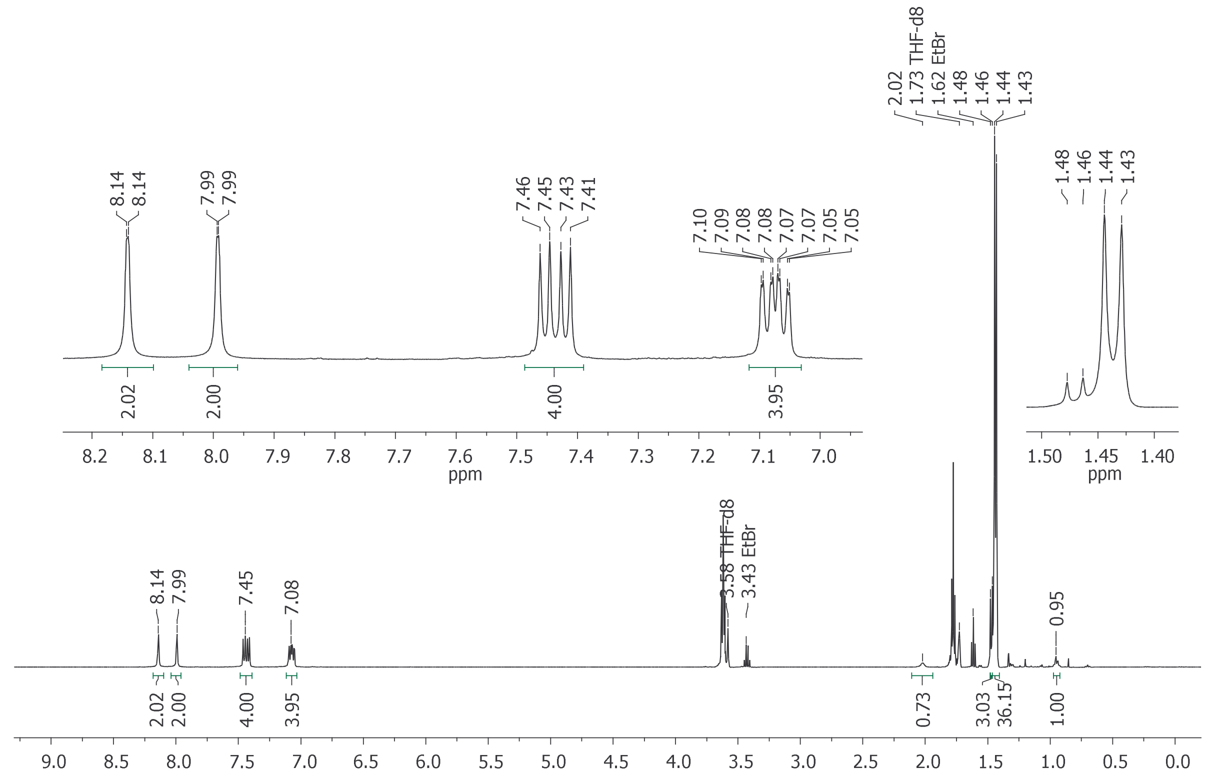

Figure S61: ^{1}H NMR spectrum of Li[**19**] (500.2 MHz, THF-d_8).

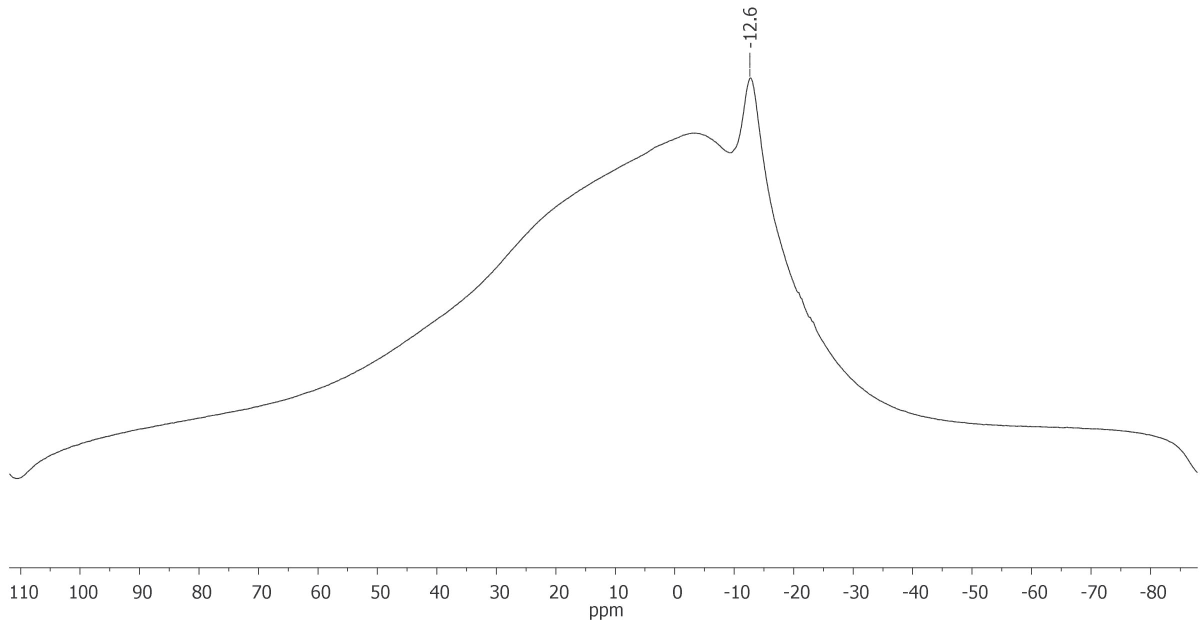

Figure S62: ^{11}B NMR spectrum of Li[**19**] (160.5 MHz, THF-d_8).

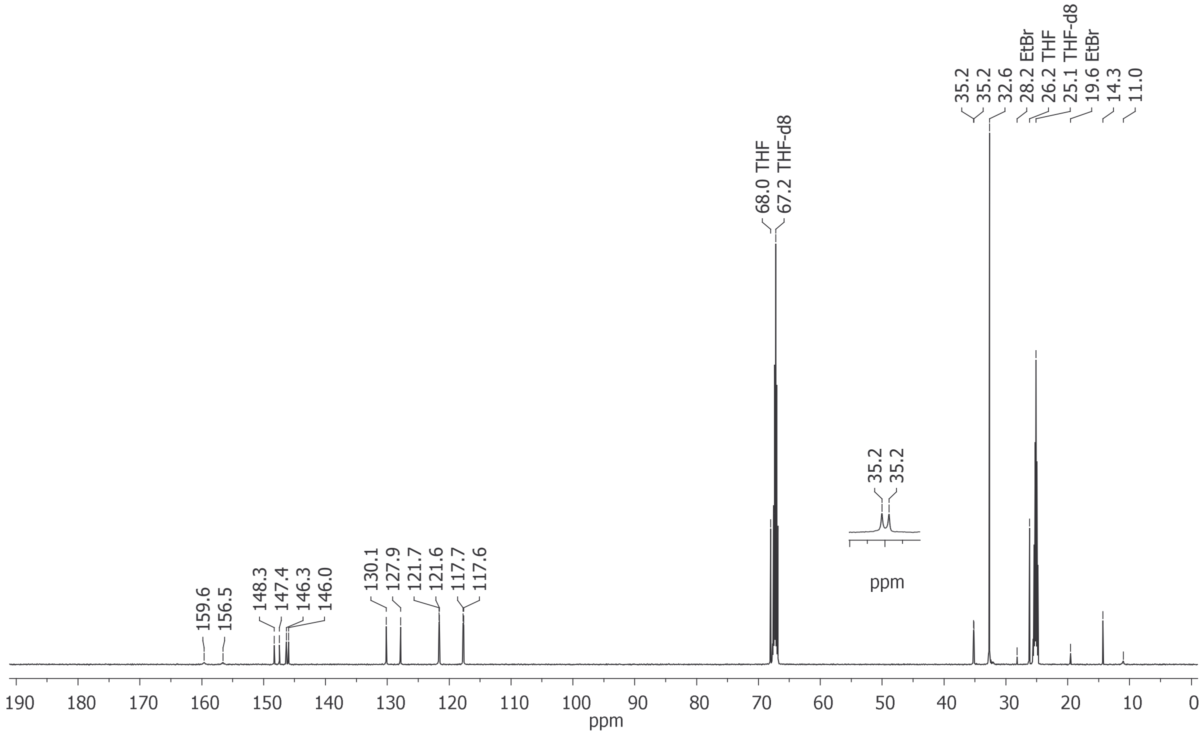

Figure S63: ^{13}C{^{1}H} NMR spectrum of Li[**19**] (125.8 MHz, THF-d_8).

3. X-ray

Data for all structures were collected on a STOE IPDS II two-circle diffractometer with a Genix Microfocus tube with mirror optics using MoK_{α} radiation (λ = 0.71073 Å). The data were scaled using the frame-scaling procedure in the *X-AREA* program system.[S6] The structures were solved by direct methods using the program *SHELXS*[S7] and refined against F^2 with full-matrix least-squares techniques using the program *SHELXL*.[S7]

Structure	Internal code	CCDC reference number
[Li(thf)$_4$][**2**]	wa2120	1819687
[Li(thf)$_3$][**2**]	wa2506	1819688
[Li(thf)$_3$(Et$_2$O)][**7**]	wa2456	1819689
[Li(12-crown-4)(thf)][Li(thf)$_2$][**11**]	wa2374	1819690
[Li(thf)$_4$][**16**]	wa2467	1819691
(**14**^{C2}·thf)·4C_6H_6	wa2473	1819692
**14**^{C3}	wa2461	1819693
**14**^{C4}	wa2477	1819694
[Li(12-crown-4)$_2$][**15**^{C5,Cl}]	wa2518	1819695

*[Li(thf)$_4$][**2**]*

Yellow single crystals of [Li(thf)$_4$][**2**] were grown by gas-phase diffusion of hexane into a THF solution of Li[**2**] (3 d, room temperature).

The H atom bridging B1 and B2 was isotropically refined; the coordinates of the two H atoms bonded to C1 were also refined. One *t*Bu group is disordered over two positions with a site occupation factor of 0.793(9) for the major occupied site. In two thf ligands, two methylene groups are disordered over two positions with site occupation factors of 0.66(2) and 0.51(2) for the major occupied sites. In one thf ligand, three methylene groups are disordered over two positions, each with a site occupation factor of 0.63(2) for the major occupied site. The lengths of the C–C bonds involving the disordered atoms in the thf ligands were restrained to 1.50(1) Å and the 1-3 distances involving disordered C atoms in the thf ligands were restrained to 2.3(1) Å or 2.3(3) Å. The displacement ellipsoids of all disordered atoms were restrained to an isotropic behavior.

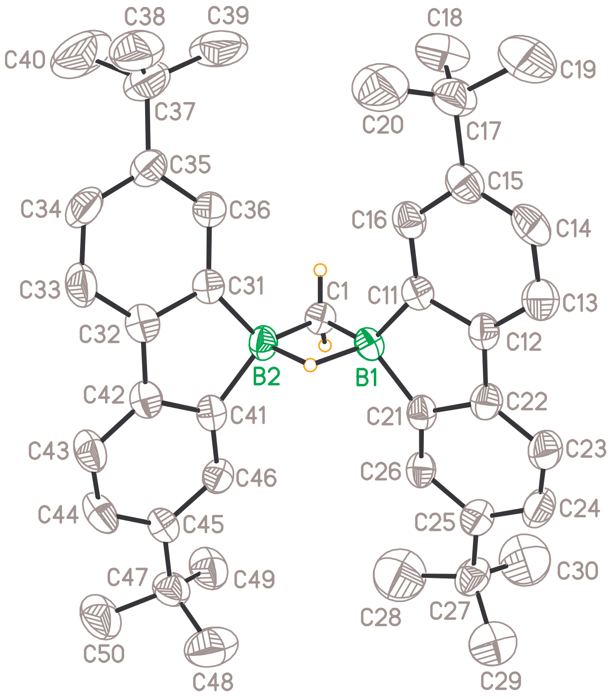

Figure S64: Molecular structure of [Li(thf)$_4$][**2**] in the solid state. The solvent-separated [Li(thf)$_4$]$^+$ cation and the C*H* atoms (except on C1) are omitted for clarity. Displacement ellipsoids are drawn at the 50% probability level. Selected atom···atom distance [Å], bond lengths [Å], and bond angles [°]: B(1)···B(2) = 1.974(6); B(1)–C(1) = 1.598(6), B(1)–C(11) = 1.624(5), B(1)–C(21) = 1.615(6), B(2)–C(1) = 1.581(6), B(2)–C(31) = 1.613(6), B(2)–C(41) = 1.629(6); B(1)–C(1)–B(2) = 76.8(3), C(11)–B(1)–C(21) = 100.7(3), C(31)–B(2)–C(41) = 100.2(3).

*[Li(thf)$_3$][**2**]*

Colorless single crystals of [Li(thf)$_3$][**2**] precipitated from a C_6H_6 solution of methyl triflate/[Li(thf)$_3$]$_2$[**1**] (1.1:1; 4 d, room temperature).

The asymmetric unit contains two crystallographically independent molecules of [Li(thf)$_3$][**2**]. The H atom bridging the boron atoms and the H atoms located on the C atoms bonded to both boron atoms were isotropically refined for both independent molecules. In the asymmetric unit, two *t*Bu groups are disordered over two positions with site occupation factors of 0.78(2) and 0.53(4) for the major occupied sites. In two thf ligands, two methylene groups are disordered over two positions with site occupation factors of 0.63(2) and 0.53(1) for the major occupied sites. In one thf ligand, three methylene groups are disordered over two positions, each with a site occupation factor of 0.62(4) for the major occupied site. The displacement ellipsoids of all atoms in the coordinating [Li(thf)$_3$]$^+$ ion were refined with a rigid bond restraint. The displacement ellipsoids of the disordered atoms were restrained to an isotropic behavior. Due to the absence of anomalous scatterers, the absolute structure could not be determined (Flack-x-parameter 1.5(10)).

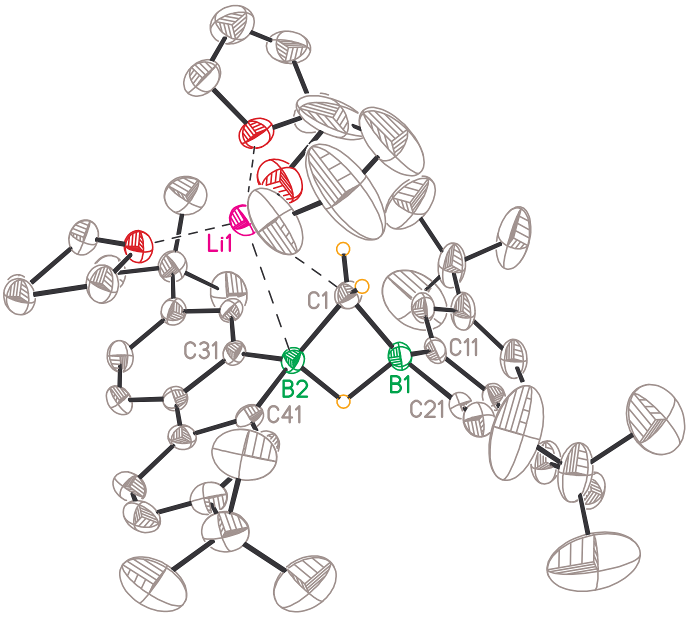

Figure S65: Molecular structure of one of the two crystallographically independent molecules of [Li(thf)$_3$][**2**] in the solid state. The C*H* atoms (except on C1) are omitted for clarity. Displacement ellipsoids are drawn at the 30% probability level. Selected atom···atom distance [Å], bond lengths [Å], and bond angles [°] of the two crystallographically independent molecules: B(1)···B(2) = 1.967(9)/1.950(9); B(1)–C(1) = 1.608(8)/1.606(8), B(1)–C(11) = 1.592(9)/1.618(8), B(1)–C(21) = 1.602(9)/1.613(9), B(2)–C(1) = 1.600(9)/1.601(8), B(2)–C(31) = 1.611(9)/1.622(9), B(2)–C(41) = 1.613(9)/1.606(9); B(1)–C(1)–B(2) = 75.7(4)/74.9(4), C(11)–B(1)–C(21) = 101.3(5)/101.1(5), C(31)–B(2)–C(41) = 100.9(5)/100.6(5).

*[Li(thf)$_3$(Et$_2$O)][**7**]*

Colorless single crystals of [Li(thf)$_3$(Et$_2$O)][**7**] precipitated from the THF/Et$_2$O filtrate of the reaction Li[HBEt$_3$]/**1**H$_2$ (1 d, room temperature).

All the H atoms bonded to B were isotropically refined. Three *t*Bu groups are disordered over two positions with site occupation factors of 0.62(1), 0.59(2), and 0.52(2) for the major occupied sites. In one thf ligand, three methylene groups are disordered over two positions, each with a site occupation factor of 0.62(2) for the major occupied site. The disordered atoms were isotropically refined. Bond lengths and bond angles of one disordered *t*Bu group were restrained to be equal to those of the non-disordered *t*Bu group. Bond lengths and bond angles of the disordered thf ligand were restrained to be equal to those of a non-disordered thf ligand.

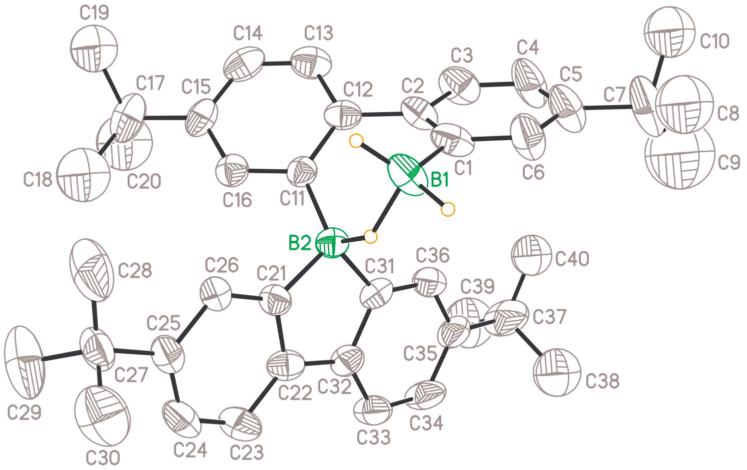

Figure S66: Molecular structure of [Li(thf)$_3$(Et$_2$O)][**7**] in the solid state. The solvent-separated [Li(thf)$_3$(Et$_2$O)]$^+$ cation and all C*H* atoms are omitted for clarity. Displacement ellipsoids are drawn at the 50% probability level. Selected atom···atom distance [Å], bond lengths [Å], bond angles [°], and torsion angle [°]: B(1)···B(2) = 2.382(8); B(1)–C(1) = 1.603(7), B(2)–C(11) = 1.623(7); B(1)–C(1)–C(2) = 122.5(5), B(2)–C(11)–C(12) = 124.6(4), C(21)–B(2)–C(31) = 100.3(3); C(1)–C(2)–C(12)–C(11) = –36.0(7).

*[Li(12-crown-4)(thf)][Li(thf)$_2$][**11**]*

The reaction mixture *t*BuCCLi/Li[**1**H] in THF was layered with hexane/12-crown-4 and stored at room temperature, whereupon orange single crystals of [Li(12-crown-4)(thf)][Li(thf)$_2$][**11**] formed.

The H atom bonded to B1 was isotropically refined. Two *t*Bu groups are disordered over two positions with site occupation factors of 0.58(2) and 0.51(2) for the major occupied sites. In one thf ligand, one methylene group is disordered over two positions with a site occupation factor of 0.79(3) for the major occupied site. The displacement ellipsoids of the disordered atoms were restrained to an isotropic behavior.

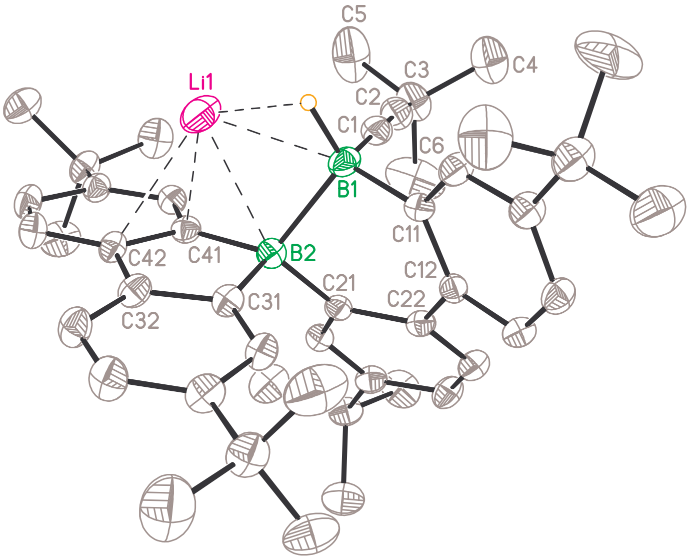

Figure S67: Molecular structure of [Li(12-crown-4)(thf)][Li(thf)$_2$][**11**] in the solid state. The solvent-separated [Li(12-crown-4)(thf)]$^+$ cation, thf ligands, and all C*H* atoms are omitted for clarity. Displacement ellipsoids are drawn at the 30% probability level. Selected bond lengths [Å], bond angles [°], and torsion angle [°]: B(1)–B(2) = 1.879(5), B(1)–C(1) = 1.612(6), B(1)–C(11) = 1.617(5), B(2)–C(21) = 1.625(5), B(2)–C(31) = 1.631(5), B(2)–C(41) = 1.630(5), C(1)–C(2) = 1.221(5); B(1)–B(2)–C(21) = 97.5(2), B(1)–C(1)–C(2) = 175.1(3), B(1)–C(11)–C(12) = 120.3(3), B(2)–B(1)–C(1) = 105.9(2), B(2)–B(1)–C(11) = 102.2(2), B(2)–C(21)–C(22) = 121.3(2), C(1)–B(1)–C(11) = 114.4(3); C(11)–C(12)–C(22)–C(21) = –31.8(4).

*[Li(thf)$_4$][**16**]*

Colorless single crystals of [Li(thf)$_4$][**16**] were grown by gas-phase diffusion of hexane into a THF solution of Li[**16**] (3 d, room temperature).

There are two crystallographically independent molecules of [Li(thf)$_4$][**16**] in the asymmetric unit. Boron-bridging H atoms were isotropically refined. In one of the independent molecules three *t*Bu groups are disordered over two positions with site occupation factors of 0.54(1), 0.75(2), and 0.83(2). In the other independent molecule, three *t*Bu groups are disordered over two positions with site occupation factors of 0.52(3), 0.55(1), and 0.57(1). In one thf ligand, the O atom and two methylene groups are disordered over two positions, each with a site occupation factor of 0.69(1) for the major occupied site. In one thf ligand, two methylene groups are disordered over two positions, each with a site occupation factor of 0.51(1) for the major occupied site. The displacement ellipsoids of all disordered atoms and all atoms in the [Li(thf)$_4$]$^+$ ions were restrained to an isotropic behavior. Bond lengths and angles in the disordered thf ligands were restrained to be equal to those in a non-disordered thf ligand.

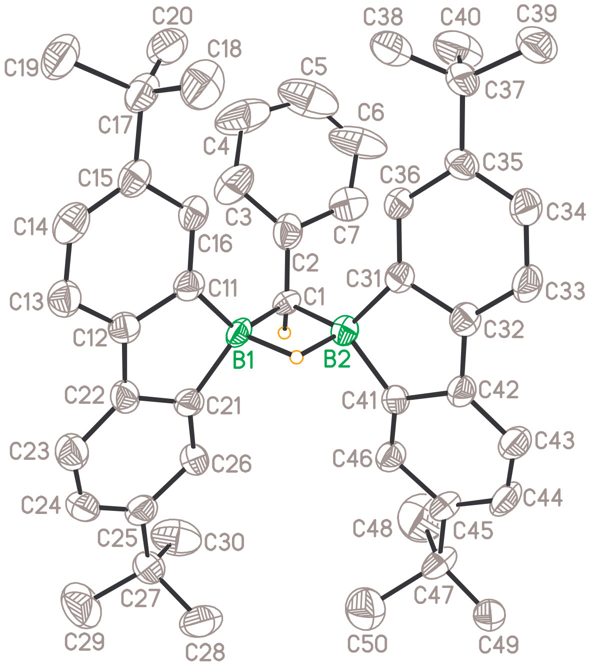

Figure S68: Molecular structure of one of the crystallographically independent molecules of [Li(thf)$_4$][**16**] in the solid state. The solvent-separated [Li(thf)$_4$]$^+$ cation and the C*H* atoms (except on C1) are omitted for clarity. Displacement ellipsoids are drawn at the 30% probability level. Selected atom···atom distance [Å], bond lengths [Å], and bond angles [°] of the two crystallographically independent molecules: B(1)···B(2) = 1.974(8)/1.990(8); B(1)–C(1) = 1.597(8)/1.625(7), B(1)–C(11) = 1.624(8)/1.615(8), B(1)–C(21) = 1.618(8)/1.601(8), B(2)–C(1) = 1.605(7)/1.613(9), B(2)–C(31) = 1.633(8)/1.609(8), B(2)–C(41) = 1.630(8)/1.602(9); B(1)–C(1)–B(2) = 76.1(4)/75.8(4), C(11)–B(1)–C(21) = 100.2(4)/102.3(4), C(31)–B(2)–C(41) = 101.0(4)/102.2(5).

($\mathbf{14}^{C2}$·thf)·4C_6H_6

Yellow single crystals of ($\mathbf{14}^{C2}$·thf)·4C_6H_6 were grown from the reaction mixture 1,2-dichloroethane/[Li(thf)$_3$]$_2$[**1**] in C_6H_6 by slow evaporation of all volatiles at room temperature.

One of the two boron atoms is tetracoordinated by the thf ligand. Two *t*Bu groups are disordered over two positions with site occupation factors of 0.818(6) and 0.61(2) for the major occupied sites. The displacement ellipsoids of the disordered atoms were restrained to an isotropic behavior.

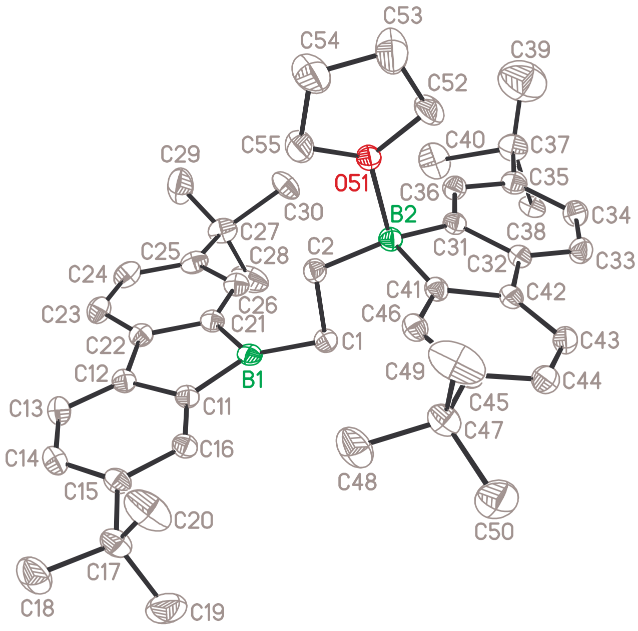

Figure S69: Molecular structure of ($\mathbf{14}^{C2}$·thf)·4C_6H_6 in the solid state. C*H* atoms and C_6H_6 molecules are omitted for clarity. Displacement ellipsoids are drawn at the 30% probability level. Selected bond lengths [Å], bond angles [°], and torsion angle [°]: B(1)–C(1) = 1.544(2), B(1)–C(11) = 1.577(2), B(1)–C(21) = 1.577(2), B(2)–C(2) = 1.609(2), B(2)–C(31) = 1.625(2), B(2)–C(41) = 1.620(2), B(2)–O(51) = 1.644(2), C(1)–C(2) = 1.566(2); C(1)–B(1)–C(11) = 128.0(1), C(1)–B(1)–C(21) = 128.3(1), C(11)–B(1)–C(21) = 102.8(1), C(2)–B(2)–C(31) = 118.0(1), C(2)–B(2)–C(41) = 118.1(1), C(31)–B(2)–C(41) = 100.0(1); B(1)–C(1)–C(2)–B(2) = –179.1(1); ∑(C–B(1)–C) = 359°; ∑(C–B(2)–C)= 336°.

14^{C3}

Yellow plates of **14**^{C3} suitable for X-ray crystallography were grown from the reaction mixture 1,3-dibromopropane/Li_2[**1**] in THF by slow evaporation of all volatiles at room temperature.

Due to the absence of anomalous scatterers, the absolute structure could not be determined (Flack-x-parameter 1.8(10)).

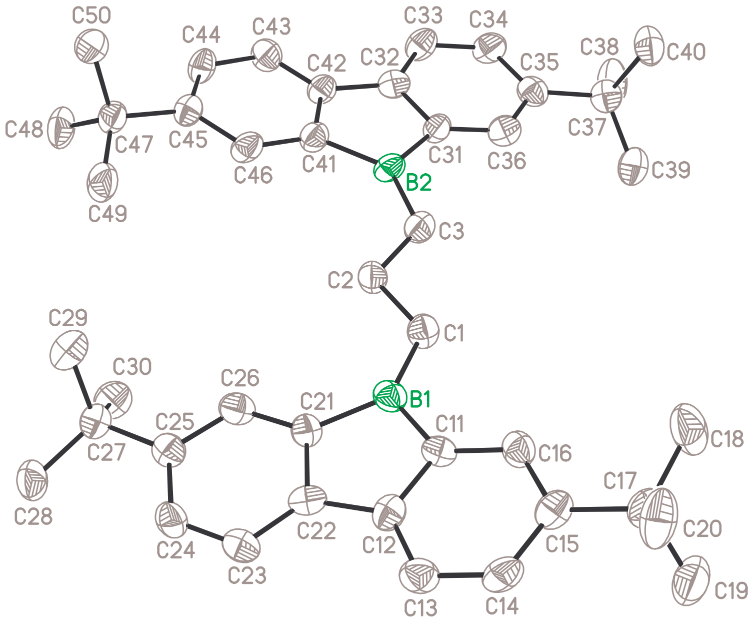

Figure S70: Molecular structure of **14**^{C3} in the solid state. C*H* atoms are omitted for clarity. Displacement ellipsoids are drawn at the 50% probability level. Selected bond lengths [Å] and bond angles [°]: B(1)–C(1) = 1.563(9), B(1)–C(11) = 1.569(9), B(1)–C(21) = 1.571(9), B(2)–C(3) = 1.577(9), B(2)–C(31) = 1.560(9), B(2)–C(41) = 1.571(10); C(1)–B(1)–C(11) = 125.1(6), C(1)–B(1)–C(21) = 131.8(6), C(11)–B(1)–C(21) = 103.0(5), C(3)–B(2)–C(31) = 121.9(6), C(3)–B(2)–C(41) = 132.6(6), C(31)–B(2)–C(41) = 105.5(5). Angle between the planes of the borol rings [°]: 22.0. ∑(C–B(1)–C) = 359.9°; ∑(C–B(2)–C) = 360.0°.

14^{C4}

Yellow single crystals of **14**^{C4} were grown from the reaction mixture 1,4-dibromobutane/Li_2[**1**] in THF by slow evaporation of all volatiles at room temperature.

The asymmetric unit contains two half crystallographically independent molecules of **14**^{C4} both located on a crystallographic center of inversion. The crystal was non-merohedrally twinned with a fractional contribution of 0.341(3) of the minor component.

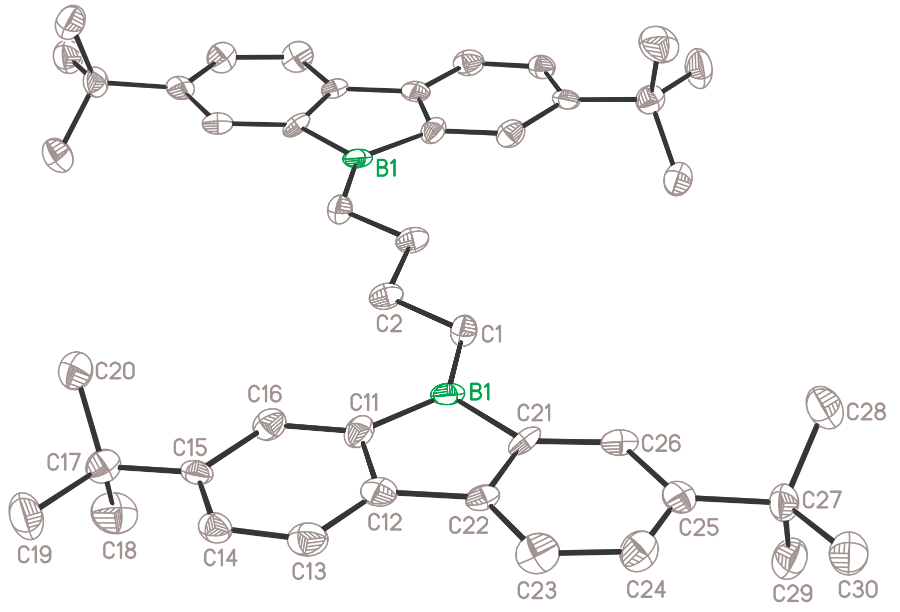

Figure S71: One of the two half crystallographically independent molecules of **14**^{C4} in the solid state. C*H* atoms are omitted for clarity. Displacement ellipsoids are drawn at the 50% probability level. Selected bond lengths [Å] and bond angles [°] of the two half crystallographically independent molecules: B(1)–C(1) = 1.566(10)/1.577(9), B(1)–C(11) = 1.558(10)/1.595(9), B(1)–C(21) = 1.579(9)/1.558(10); C(1)–B(1)–C(11) = 131.7(6)/125.4(6), C(1)–B(1)–C(21) = 125.1(6)/131.4(5), C(11)–B(1)–C(21) = 103.2(6)/103.1(5). Angle between the planes of the borol rings [°]: 0.0. ∑(C–B(1)–C) = 360.0°/359.9°. Symmetry transformations used to generate equivalent atoms: 1) –x+1, –y+1, –z–1; 2) –x, –y+1, –z–2.

*[Li(12-crown-4)$_2$][**15**^{C5,Cl}]*

The reaction mixture 1,5-dichloropentane/Li$_2$[**1**] in THF was layered with hexane/12-crown-4 and stored at room temperature, whereupon colorless single crystals of [Li(12-crown-4)$_2$][**15**^{C5,Cl}] formed.

The asymmetric unit contains one-half of a molecule of [Li(12-crown-4)$_2$][**15**^{C5,Cl}] located on a crystallographic C_2 axis. Three methylene groups and the terminal Cl atom of the pentyl chain are disordered over the C_2 axis with two equally occupied positions. One *t*Bu group is disordered over two positions with a site occupation factor of 0.83(3) for the major occupied site. The C–C bond lengths in the pentyl chain were restrained to 1.50(1) Å and 1-3 C-C distances in this chain were restrained to 2.50(1) Å. The C–Cl bond length was restrained to 1.80(1) Å. The displacement ellipsoids of the disordered C atoms were restrained to an isotropic behavior. The Flack-x-parameter refined to –0.2(2). The boron-bridging H atom was isotropically refined.

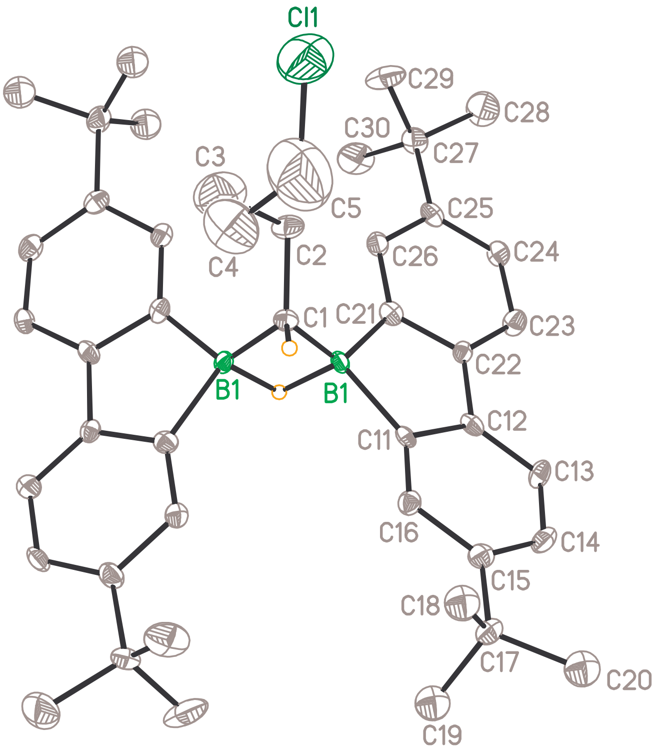

Figure S72: Molecular structure of [Li(12-crown-4)$_2$][**15**^{C5,Cl}] in the solid state. The solvent-separated [Li(12-crown-4)$_2$]$^+$ cation and C*H* atoms (except on C1) are omitted for clarity. Displacement ellipsoids are drawn at the 30% probability level. Selected atom···atom distance [Å], bond lengths [Å], and bond angles [°]: B(1)···B(1) = 1.93(1); B(1)–C(1) = 1.60(1), B(1)–C(11) = 1.656(9), B(1)–C(21) = 1.643(9); C(1)–B(1)–C(11) = 122.8(5), C(1)–B(1)–C(21) = 124.6(5), C(11)–B(1)–C(21) = 98.6(5). Symmetry transformation used to generate equivalent atoms: –x+1, –y+1, z.

Table S2. Selected crystallographic data for [Li(thf)$_4$][**2**], [Li(thf)$_3$][**2**], and [Li(thf)$_3$(Et$_2$O)][**7**].

	[Li(thf)$_4$][**2**]	[Li(thf)$_3$][**2**]	[Li(thf)$_3$(Et$_2$O)][**7**]
formula	$C_{57}H_{83}B_2LiO_4$	$C_{53}H_{75}B_2LiO_3$	$C_{56}H_{85}B_2LiO_4$
M_r	860.79	788.69	850.79
color, shape	colorless, needle	colorless, block	colorless, needle
T [K]	173(2)	173(2)	173(2)
radiation, λ [Å]	MoK_α, 0.71073	MoK_α, 0.71073	MoK_α, 0.71073
crystal system	orthorhombic	monoclinic	monoclinic
space group	*Pccn*	$P2_1$	$P2_1/n$
a [Å]	21.5605(15)	14.7750(10)	10.7242(12)
b [Å]	26.1802(19)	19.9973(10)	21.4641(16)
c [Å]	19.2608(13)	17.9098(12)	24.027(3)
α [°]	90	90	90
β [°]	90	109.105(5)	97.208(9)
γ [°]	90	90	90
V [Å^3]	10871.9(13)	5000.2(6)	5487.0(10)
Z	8	4	4
D_{calcd} [g cm^{-3}]	1.052	1.048	1.030
μ [mm^{-1}]	0.063	0.062	0.061
F(000)	3760	1720	1864
crystal size [mm]	0.28 x 0.18 x 0.12	0.24 x 0.22 x 0.17	0.21 x 0.11 x 0.02
rflns collected	42030	44665	66414
independent rflns (R_{int})	9821 (0.1664)	18711 (0.0674)	10391 (0.1431)
data/restraints/parameters	9821 / 190 / 681	18711 / 484 / 1209	10391 / 164 / 572
GOF on F^2	0.946	0.910	1.443
R_1, wR_2 [$I > 2\sigma(I)$]	0.0860, 0.1598	0.0712, 0.1404	0.1344, 0.2573
R_1, wR_2 (all data)	0.2038, 0.2033	0.1458, 0.1670	0.2251, 0.2877
largest diff peak and hole [e Å^{-3}]	0.418, –0.233	0.421, –0.211	0.933, –0.296

Table S3. Selected crystallographic data for [Li(12-crown-4)(thf)][Li(thf)$_2$][**11**], [Li(thf)$_4$][**16**], and (**14**^{C2}·thf)·4C_6H_6.

	[Li(12-crown-4)(thf)][Li(thf)$_2$][**11**]	[Li(thf)$_4$][**16**]	(**14**^{C2}·thf)·4C_6H_6
formula	$C_{66}H_{98}B_2Li_2O_7$	$C_{63}H_{87}B_2LiO_4$	$C_{70}H_{84}B_2O$
M_r	1038.94	936.88	962.99
color, shape	orange, plate	colorless, plate	yellow, block
T [K]	173(2)	173(2)	173(2)
radiation, λ [Å]	MoK_α, 0.71073	MoK_α, 0.71073	MoK_α, 0.71073
crystal system	monoclinic	monoclinic	triclinic
space group	$P2_1/c$	$P2_1/c$	P-1
a [Å]	12.9451(8)	19.4970(15)	10.4041(3)
b [Å]	18.3459(7)	24.7864(18)	11.5484(3)
c [Å]	27.0962(17)	23.972(2)	25.7360(7)
α [°]	90	90	79.323(2)
β [°]	101.393(5)	92.463(7)	85.313(2)
γ [°]	90	90	81.220(2)
V [Å^3]	6308.3(6)	11574.0(16)	2998.51(14)
Z	4	8	2
D_{calcd} [g cm^{-3}]	1.094	1.075	1.067
μ [mm^{-1}]	0.068	0.064	0.060
F(000)	2264	4080	1044
crystal size [mm]	0.24 x 0.19 x 0.04	0.17 x 0.17 x 0.03	0.24 x 0.19 x 0.14
rflns collected	59630	87491	69450
independent rflns (R_{int})	11144 (0.0915)	21394 (0.1445)	12158 (0.0402)
data/restraints/parameters	11144 / 72 / 764	21394 / 891 / 1484	12158 / 84 / 714
GOF on F^2	1.134	1.302	1.037
R_1, wR_2 [$I > 2\sigma(I)$]	0.0785, 0.1585	0.1210, 0.2070	0.0673, 0.1755
R_1, wR_2 (all data)	0.1447, 0.1797	0.2818, 0.2432	0.0782, 0.1838
largest diff peak and hole [e Å^{-3}]	0.306, –0.212	0.704, –0.334	0.539, –0.424

Table S4. Selected crystallographic data for **14**^{C3}, **14**^{C4}, and [Li(12-crown-4)$_2$][**15**^{C5,Cl}].

	**14**^{C3}	**14**^{C4}	[Li(12-crown-4)$_2$][**15**^{C5,Cl}]
formula	$C_{43}H_{54}B_2$	$C_{44}H_{56}B_2$	$C_{61}H_{90}B_2ClLiO_8$
M_r	592.48	606.50	1015.33
color, shape	yellow, plate	yellow, block	colorless, plate
T [K]	173(2)	173(2)	173(2)
radiation, λ [Å]	MoK_α, 0.71073	MoK_α, 0.71073	MoK_α, 0.71073
crystal system	orthorhombic	triclinic	orthorhombic
space group	$Pca2_1$	P-1	Fdd2
a [Å]	41.344(3)	5.9740(9)	12.2698(10)
b [Å]	14.2720(12)	14.639(2)	43.946(3)
c [Å]	6.1349(5)	21.461(3)	21.961(2)
α [°]	90	82.273(12)	90
β [°]	90	89.910(12)	90
γ [°]	90	86.790(12)	90
V [Å^3]	3620.0(5)	1856.9(5)	11841.6(17)
Z	4	2	8
D_{calcd} [g cm^{-3}]	1.087	1.085	1.139
μ [mm^{-1}]	0.060	0.060	0.116
F(000)	1288	660	4400
crystal size [mm]	0.17 x 0.13 x 0.08	0.14 x 0.09 x 0.04	0.26 x 0.24 x 0.11
rflns collected	24978	16800	29925
independent rflns (R_{int})	6651 (0.1309)	16800	5586 (0.1140)
data/restraints/parameters	6651 / 1 / 406	16800 / 0 / 416	5586 / 63 / 379
GOF on F^2	0.840	1.207	1.121
R_1, wR_2 [$I > 2\sigma(I)$]	0.0673, 0.1049	0.0880, 0.1686	0.0854, 0.1603
R_1, wR_2 (all data)	0.1448, 0.1282	0.1850, 0.1839	0.1296, 0.1780
largest diff peak and hole [e Å^{-3}]	0.177, –0.162	0.359, –0.328	0.649, –0.220

4. Computational details

DFT calculations were carried out with the Gaussian program package.[S8] The PBE0[S9-12] hybrid functional was used and combined with the D3BJ atom-pairwise dispersion correction with Becke-Johnson damping as devised by Grimme.[S13, 14] Geometry optimizations and harmonic frequency calculations were computed under gas-phase conditions with the TZVP basis set.[S15] All stationary points reported were characterized as minima or first order saddle points by eigenvalue analysis of the diagonalized Hessians. Gibbs energies reported correspond to the total energies of single point calculations with the SMD solvation model[S16] (to account for effects of the THF solvent), corrected by thermal contributions from the gas-phase frequency analyses. Graphical representations of molecular geometries were produced with the *CYLview* software.[S17]

Minima

$[5^{c}]^{-}$

Total energy = –1013.13778138 Hartree

Thermal correction to Gibbs free energy = 0.315916 Hartree

d(B···B) = 1.690623 Å

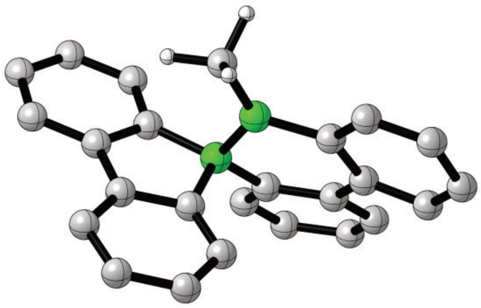

B	-0.441967198	0.464857702	0.127632617	C	2.890633354	2.347533655	-0.658552086
B	0.225700371	-0.679245183	1.178087245	C	1.061197048	3.771214531	-1.248071155
C	-0.571464517	-1.630514359	2.156272199	H	-0.881937078	2.945367287	-0.914556789
C	-1.938766773	0.851279774	0.488876060	C	3.925080647	-0.252221994	0.063459838
C	-2.827741254	-0.138378499	0.005023636	C	3.779272020	-2.202133390	1.454558172
C	-0.711703468	-0.832404114	-0.809277259	H	1.838040302	-2.610152226	2.237639834
C	0.621563346	1.537209407	-0.350327269	H	-3.671719519	-2.469913882	-1.396959802
C	1.790603414	-0.837354507	1.044791777	C	-1.754129712	-3.067852268	-2.157963939
C	-2.481883990	1.890935817	1.244952002	H	-5.765328215	1.008762496	1.245390892
C	-2.100180930	-1.136160851	-0.769401309	H	3.960676850	2.217991538	-0.544309920
C	-4.192188896	-0.093660389	0.284151242	C	2.430670628	3.557561064	-1.145398242
C	0.122999207	-1.678474373	-1.542352293	H	4.525956006	0.373420776	-0.585496480
C	2.015866005	1.321973362	-0.269736172	C	4.521762246	-1.360580706	0.636620842
C	0.188216342	2.775086435	-0.846665187	H	-2.148048827	-3.933502799	-2.681881169
C	2.574236105	0.061804526	0.278656991	H	3.136513462	4.331636258	-1.430343275
C	2.433904652	-1.930841048	1.636349844	H	5.568849148	-1.569562460	0.438157369
C	-3.845293467	1.951460778	1.505035807	H	-4.250553999	2.775549227	2.086047459
H	-1.828623917	2.667125114	1.635910729	H	0.269230697	-3.416972514	-2.794549574
C	-2.608635995	-2.247157632	-1.433386684	H	0.679336625	4.714925827	-1.627555126
H	-4.857533294	-0.870726355	-0.083265376	H	4.237309131	-3.072275506	1.914749260
C	-4.702154460	0.957599006	1.030913842	H	-0.291522991	-1.381184548	3.189353696
C	-0.393309405	-2.776983353	-2.219119525	H	-0.345561851	-2.695052685	2.022475016
H	1.188190946	-1.474342020	-1.587150586	H	-1.653252231	-1.502801358	2.071277747

[6^c-open]$^{-}$

Total energy = –1013.12607437 Hartree

Thermal correction to Gibbs free energy = 0.313661 Hartree

d(B···B) = 1.689778 Å

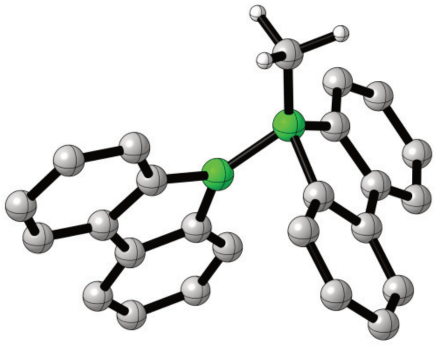

B	0.720147961	-1.135240335	1.206158149	C	-2.207807885	2.932925812	-0.745189863
B	-0.549192749	-0.161076673	0.662901704	H	-3.241465759	3.230229136	-0.898166136
C	0.475381457	-2.271006271	2.337009714	C	-1.176257495	3.810335799	-1.063658056
C	2.107279432	-0.341259185	1.227308617	H	-1.406260279	4.790900996	-1.469774313
C	2.692089520	-0.305127243	-0.060500408	C	0.146015628	3.438696737	-0.853273124
C	3.870833210	0.392946363	-0.311718541	C	0.453118986	2.186457363	-0.325406501
H	4.294454186	0.424204145	-1.312194215	H	1.488416357	1.918887820	-0.149663413
C	4.503498912	1.057829213	0.729161353	C	-2.113139427	-0.497714940	0.666975322
H	5.424616629	1.603347954	0.548017743	C	-2.824831333	0.616940320	0.174036316
C	3.954268169	1.020657011	2.010469389	C	-4.210642339	0.627837411	0.120454105
C	2.770238150	0.333077728	2.251637414	H	-4.747139950	1.493495207	-0.257575647
H	2.350404367	0.330345323	3.254561673	C	-4.915040203	-0.492077329	0.552979444
C	0.726379746	-1.594191318	-0.364092128	H	-6.000082420	-0.497656020	0.511022710
C	1.867049287	-1.042353773	-1.009848143	C	-4.234519413	-1.599715469	1.042115433
C	2.093775785	-1.243672477	-2.365712580	C	-2.841835243	-1.595786719	1.105114934
H	2.969671092	-0.817575202	-2.847409694	H	-2.323323884	-2.460950440	1.503699835
C	1.196648612	-2.002007008	-3.107556384	H	4.455712783	1.540230934	2.822632090
H	1.369674701	-2.161960115	-4.167558667	H	-0.622334772	-3.146285889	-3.081063662
C	0.075619899	-2.555134335	-2.495549713	H	-4.791349294	-2.467709764	1.382026822
C	-0.161591610	-2.340677439	-1.142639316	H	0.945655281	4.131920197	-1.095852287
H	-1.053375709	-2.759990626	-0.687620430	H	1.350242848	-2.926411213	2.437180993
C	-0.557471678	1.282145551	-0.018741001	H	-0.378713997	-2.920819903	2.119088638
C	-1.893869290	1.682435291	-0.233629283	H	0.289276503	-1.831984054	3.324866689

[6^c]^–

Total energy = –1013.11924082 Hartree

Thermal correction to Gibbs free energy = 0.314099 Hartree

d(B···B) = 1.672597 Å

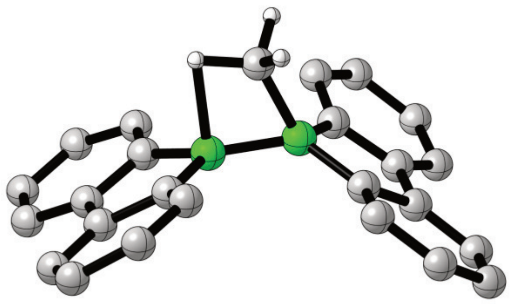

B	0.845333366	-0.123641900	0.772344246	C	-3.658202096	1.875976237	-1.047446566
B	-0.807549224	0.080432464	0.617700224	H	-4.568694722	1.532485257	-1.530240970
C	0.077242733	-0.080809613	2.284120336	C	-3.344734473	3.228556034	-1.038508614
C	1.911146323	1.023933395	0.507228162	H	-4.008159542	3.944255501	-1.514285535
C	2.965688132	0.488105971	-0.269678648	C	-2.177337343	3.663263881	-0.420131263
C	4.031699809	1.274072372	-0.692740603	C	-1.310724712	2.752423546	0.175642605
H	4.825647376	0.847374852	-1.299841548	H	-0.390213579	3.116332854	0.611855638
C	4.097155421	2.607159203	-0.307457188	C	-1.916075530	-1.042969577	0.439254445
H	4.928294139	3.227503641	-0.628810687	C	-3.000807711	-0.470859556	-0.264487730
C	3.109211753	3.135863190	0.517809126	C	-4.100799084	-1.228516824	-0.646247436
C	2.034892629	2.348293737	0.919957177	H	-4.917981915	-0.776780221	-1.201440312
H	1.294911754	2.781270253	1.587981390	C	-4.165863164	-2.568023405	-0.283349670
C	1.624675988	-1.403483736	0.233431984	H	-5.023602837	-3.168223002	-0.570794519
C	2.787725157	-0.953549612	-0.443888906	C	-3.141700557	-3.132204585	0.469551434
C	3.631371688	-1.830950653	-1.115796911	C	-2.029527872	-2.374671576	0.824178305
H	4.508748673	-1.458613278	-1.637883046	H	-1.253878713	-2.835856453	1.425006792
C	3.358714685	-3.192032747	-1.104338715	H	3.180271281	4.167277156	0.851503195
H	4.014996205	-3.885726795	-1.621060199	H	2.031964048	-4.727109934	-0.398058682
C	2.242467680	-3.661485132	-0.419435726	H	-3.209127116	-4.170871442	0.779001807
C	1.388844473	-2.777275987	0.232280537	H	-1.932252485	4.721175128	-0.414856139
H	0.520321620	-3.181839781	0.736922938	H	0.593359029	0.695994150	2.846381238
C	-1.601038313	1.391052471	0.192733423	H	0.163282023	-1.063683775	2.744886097
C	-2.805037684	0.972661461	-0.425635411	H	-1.000733532	0.183912091	2.381245301

[2^c-open][–]

Total energy = –1013.14164203 Hartree

Thermal correction to Gibbs free energy = 0.314067 Hartree

d(B···B) = 2.343491 Å

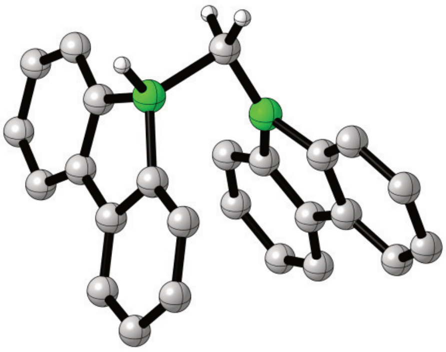

B	-0.656663047	0.008448156	-1.578232600	H	-2.917086865	-1.856388644	-2.246277428
B	0.924016051	-1.684198359	-1.219971128	C	0.299339576	-1.673813670	0.292200336
H	1.017934020	-2.843701814	-1.603452847	C	1.236892488	-1.109852867	1.186128070
C	0.142922501	-0.848116115	-2.517753641	C	0.983672394	-1.019746706	2.551161843
H	0.950937694	-0.390977444	-3.087574191	H	1.714156930	-0.572967603	3.220138618
H	-0.411756896	-1.553356816	-3.137115487	C	-0.212434246	-1.507909808	3.056828161
C	-0.233492773	1.295074225	-0.752204824	H	-0.425250549	-1.434156723	4.119193099
C	-1.340887837	1.716773899	0.012962834	C	-1.136934397	-2.097707252	2.199545892
C	-1.255233840	2.808323648	0.861954730	C	-0.875954245	-2.184478780	0.836902094
H	-2.107465819	3.122025942	1.457702449	H	-1.617641300	-2.638772552	0.188007235
C	-0.048127766	3.496015481	0.956074629	C	2.343752771	-1.002175372	-0.895709477
H	0.035877837	4.347177895	1.625189262	C	2.444713667	-0.689086681	0.475305368
C	1.048614321	3.098790044	0.202820179	C	3.564631525	-0.044741675	0.993175210
C	0.955165254	2.002415374	-0.652640691	H	3.621250258	0.205151715	2.049055912
H	1.823755548	1.688049053	-1.220978677	C	4.607714414	0.298701199	0.143753230
C	-2.177616684	-0.166891663	-1.154964839	H	5.483187427	0.807557430	0.536282149
C	-2.507870706	0.849718191	-0.237806679	C	4.528537121	-0.002973123	-1.213724359
C	-3.782458415	0.942094725	0.299247034	C	3.403305458	-0.645157135	-1.722914767
H	-4.033330375	1.722011839	1.012091689	H	3.350158686	-0.861471597	-2.787930465
C	-4.747091092	0.013802753	-0.083757519	H	1.987562058	3.636064101	0.290855320
H	-5.747503219	0.073508471	0.334205969	H	-5.203239645	-1.700729086	-1.291509528
C	-4.440339331	-0.985733486	-0.998858985	H	5.347471273	0.269255967	-1.874009700
C	-3.156485385	-1.069446907	-1.535964779	H	-2.071790717	-2.482798898	2.596590971

[2^c]^−

Total energy = –1013.15160474 Hartree

Thermal correction to Gibbs free energy = 0.314029 Hartree

d(B···B) = 1.916075 Å

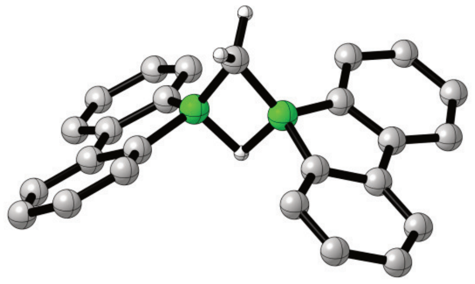

B	-0.955185592	0.073866560	0.721830180	H	-0.664912282	3.116995251	0.555495530
B	0.955186640	-0.073860255	0.721835402	C	1.810984502	-1.335790407	0.199526715
H	0.000005965	0.000004772	-0.268992240	C	3.028757590	-0.861326347	-0.335320007
C	-0.000002751	0.000006632	1.989491668	C	3.975623742	-1.724762292	-0.875046273
H	-0.106671534	-0.895803478	2.597947110	H	4.906657646	-1.339141390	-1.280893378
H	0.106663546	0.895820250	2.597942444	C	3.724136909	-3.089970727	-0.892694259
C	-1.974809555	-1.127350574	0.403057252	H	4.456065929	-3.773359331	-1.312370606
C	-3.130852473	-0.596042867	-0.204010831	C	2.532335276	-3.578367488	-0.370407143
C	-4.208915011	-1.404252671	-0.548948199	C	1.588411209	-2.707139139	0.166932989
H	-5.092030973	-0.978812030	-1.017207477	H	0.664915124	-3.116987462	0.555537018
C	-4.158561339	-2.763941782	-0.271886902	C	1.974815798	1.127353700	0.403062041
H	-4.993781339	-3.404623202	-0.537894536	C	3.130849380	0.596043324	-0.204021906
C	-3.046019733	-3.299191883	0.367804175	C	4.208911939	1.404249455	-0.548967829
C	-1.971703394	-2.482050495	0.708189535	H	5.092019595	0.978807176	-1.017241312
H	-1.132767190	-2.915041641	1.242199339	C	4.158570454	2.763936496	-0.271894307
C	-1.810986117	1.335794505	0.199518562	H	4.993791129	3.404614873	-0.537907127
C	-3.028765814	0.861326733	-0.335310789	C	3.046042646	3.299187182	0.367820076
C	-3.975639755	1.724758354	-0.875030380	C	1.971725416	2.482049634	0.708211997
H	-4.906678364	1.339133907	-1.280863305	H	1.132802738	2.915039466	1.242243826
C	-3.724153982	3.089966662	-0.892691613	H	-3.019897471	-4.357638630	0.609566433
H	-4.456088851	3.773351994	-1.312363091	H	-2.335130439	4.646270355	-0.383184734
C	-2.532344995	3.578367056	-0.370425063	H	3.019932866	4.357630902	0.609596906
C	-1.588413742	2.707143231	0.166909763	H	2.335121465	-4.646271065	-0.383154341

Transition states

TS1

Total energy = –1013.12056760 Hartree
Thermal correction to Gibbs free energy = 0.314542 Hartree
Imaginary frequency: –241.0656
d(B···B) = 1.634340 Å

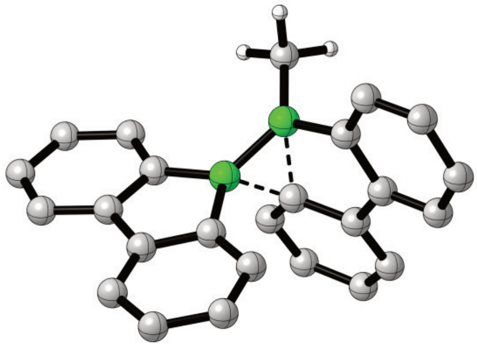

B	0.532998293	-0.156226601	0.510418886	C	-2.332863407	-1.646867975	-2.024923581
B	-0.667226025	-0.804368792	1.410663562	C	-0.283071371	-2.874281753	-2.259244957
C	-0.553161829	-2.016399372	2.447619510	H	1.195730954	-2.534373237	-0.754485773
C	2.075325768	-0.549017900	0.589012778	C	-3.855763309	0.422129148	-0.291187441
C	2.839761795	0.557332944	0.147866108	C	-3.923133719	1.346497746	1.926568231
C	0.606033215	1.257543425	-0.225146057	H	-2.283496348	0.857529103	3.218509008
C	-0.555087955	-1.352153855	-0.386554408	H	3.397298331	3.126564701	-0.939987344
C	-2.060188990	-0.065650481	1.290546212	C	1.371994037	3.741356758	-1.309974935
C	2.770341313	-1.664867887	1.050788260	H	5.977400884	-0.600765990	0.690035613
C	1.966145635	1.627744077	-0.341314402	H	-3.339735824	-1.426654581	-2.366297403
C	4.228942299	0.547493095	0.192802324	C	-1.545744857	-2.543821112	-2.731270235
C	-0.349488972	2.166857431	-0.674472294	H	-4.291254811	0.317120145	-1.280895848
C	-1.853824746	-1.056819031	-0.859252152	C	-4.492950802	1.202551230	0.665359924
C	0.198044614	-2.284510732	-1.096112911	H	1.657054071	4.702382717	-1.727767491
C	-2.654246998	-0.202386610	0.023150554	H	-1.926245047	-3.000348311	-3.639972971
C	-2.719186780	0.721078624	2.232430163	H	-5.429262162	1.697915253	0.429073487
C	4.162055769	-1.687808262	1.079834916	H	4.681681833	-2.571176325	1.440108927
H	2.225893913	-2.537211076	1.399095596	H	-0.734154006	4.084823005	-1.559802771
C	2.346801163	2.857790069	-0.866882290	H	0.332935918	-3.586499585	-2.799567244
H	4.798523461	1.410671502	-0.141085003	H	-4.420515301	1.958256784	2.673827754
C	4.892159109	-0.581740612	0.657393965	H	-1.356304931	-2.755008876	2.332318289
C	0.027769940	3.392078610	-1.213751807	H	-0.652129201	-1.606437972	3.461760607
H	-1.403822179	1.927068479	-0.596142088	H	0.401573558	-2.545644205	2.416149554

TS2

Total energy = –1013.11549001 Hartree

Thermal correction to Gibbs free energy = 0.314249 Hartree

Imaginary frequency: –116.0814

d(B···B) = 1.674970 Å

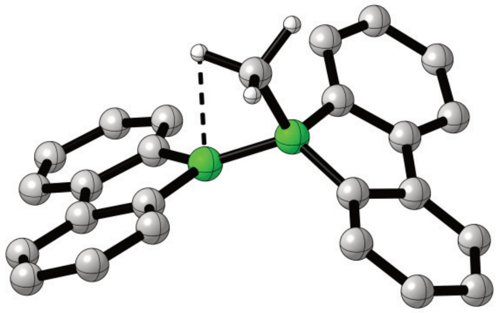

B	0.823342668	-0.244773625	0.748845532	C	-3.779222362	1.949358951	-0.897073309
B	-0.775530180	-0.002793182	0.312296136	H	-4.800587335	1.680584218	-1.151034249
C	0.245781499	-0.433839904	2.320737283	C	-3.338032719	3.256957505	-1.077488521
C	1.874189686	0.960366612	0.609823352	H	-4.020655805	4.008994398	-1.461679977
C	2.995656592	0.531612607	-0.135256788	C	-2.024979546	3.598944777	-0.781508326
C	4.055729049	1.387612102	-0.418270504	C	-1.138372247	2.637280085	-0.298905615
H	4.904705461	1.042874316	-1.003023866	H	-0.106819922	2.904551694	-0.104636474
C	4.038994652	2.685408280	0.076717677	C	-1.980782722	-1.057811375	0.324343146
H	4.863940888	3.359636418	-0.133492584	C	-3.152637991	-0.422763904	-0.139125228
C	2.973484474	3.110774366	0.865420652	C	-4.348592200	-1.109833636	-0.279895649
C	1.909017113	2.253020758	1.128641237	H	-5.239788918	-0.608804377	-0.646750542
H	1.092765711	2.603198925	1.756740905	C	-4.402588526	-2.454489610	0.074878891
C	1.689406367	-1.432904928	0.097334668	H	-5.333744045	-3.003742831	-0.025225322
C	2.877654996	-0.892209126	-0.454537476	C	-3.270766734	-3.091522179	0.565774472
C	3.786627753	-1.678485902	-1.155458175	C	-2.069571675	-2.394824505	0.686813471
H	4.686343358	-1.237177189	-1.576624653	H	-1.199905856	-2.906526757	1.081501589
C	3.546826044	-3.037545486	-1.306384032	H	2.976986258	4.116013743	1.278263332
H	4.252715968	-3.660653302	-1.847411436	H	2.212191950	-4.662077715	-0.863685225
C	2.398562045	-3.597091645	-0.754517562	H	-3.322354235	-4.137045622	0.853546030
C	1.484831065	-2.801044498	-0.070361347	H	-1.684300195	4.617519972	-0.939902567
H	0.593059152	-3.271048158	0.330681157	H	1.030426183	-0.074730649	2.995503314
C	-1.560958050	1.332784367	-0.078880860	H	0.034362507	-1.483476412	2.535623845
C	-2.896216991	1.004016523	-0.398310750	H	-0.662640982	0.127121936	2.588834136

TS3

Total energy = –1013.11106266 Hartree

Thermal correction to Gibbs free energy = 0.312907 Hartree

Imaginary frequencies: –686.9080

d(B···B) = 1.870943 Å

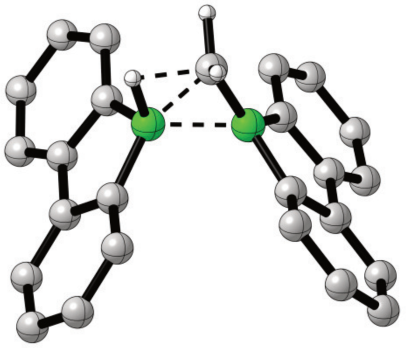

B	-0.826974644	-0.379670080	1.228647026	C	3.784888128	-0.226617992	-1.003987811
B	0.869216144	0.409290660	1.258752301	H	4.230023499	0.333176114	-1.821752333
C	-0.157148429	-0.114867485	2.582327985	C	4.328632723	-1.445853137	-0.622780608
C	-0.965290065	-1.731926439	0.417308576	H	5.188900221	-1.846492265	-1.149976942
C	-2.042357505	-1.581665417	-0.491538883	C	3.779169040	-2.146749362	0.447696255
C	-2.404303002	-2.599518136	-1.365977238	C	2.669867318	-1.646951036	1.119489784
H	-3.229868750	-2.462057888	-2.058960260	H	2.266831381	-2.207054618	1.959087211
C	-1.709414607	-3.801646399	-1.344806247	C	0.988181579	1.756957228	0.448424374
H	-1.983469228	-4.602010106	-2.025389659	C	2.029657571	1.574882938	-0.498024387
C	-0.663071697	-3.977452349	-0.444706536	C	2.362267196	2.574069487	-1.406538286
C	-0.299580413	-2.954788057	0.424598038	H	3.155354505	2.412855843	-2.131468501
H	0.526696546	-3.120090963	1.105019723	C	1.685920133	3.785148543	-1.376667328
C	-2.055296851	0.463478017	0.699342430	H	1.939119479	4.569248437	-2.083455039
C	-2.695691655	-0.281274328	-0.317942572	C	0.683037596	3.991357564	-0.433094901
C	-3.827604294	0.200651032	-0.964542422	C	0.341034825	2.990101931	0.467032040
H	-4.305769951	-0.380983037	-1.747854321	H	-0.452893211	3.178158639	1.178980811
C	-4.358244028	1.429677874	-0.592313467	H	-0.123188921	-4.919745480	-0.423527273
H	-5.240791589	1.815294005	-1.093564320	H	-4.199025727	3.106225121	0.741663578
C	-3.768061689	2.156902526	0.436830782	H	0.158448108	4.941896192	-0.403778321
C	-2.632045577	1.671539225	1.076458578	H	4.220118393	-3.089863168	0.757103700
H	-2.203706275	2.248228426	1.891928144	H	0.296781094	-0.930817938	3.140689487
C	2.083264133	-0.444663988	0.736224173	H	-0.591123166	0.647772130	3.226706021
C	2.681965907	0.277821850	-0.323149301	H	1.097698094	0.646473120	2.483573491

TS4

Total energy = –1013.13848805 Hartree

Thermal correction to Gibbs free energy = 0.315252 Hartree

Imaginary frequency: –13.9254

d(B···B) = 2.377687 Å

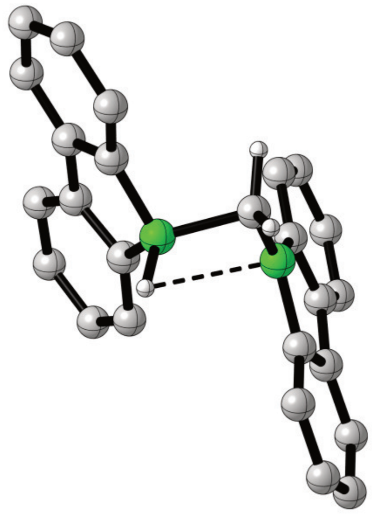

B	0.811745936	-0.902545467	0.987350017	H	1.752775470	-3.451981186	-0.289694671
B	-1.175652854	-1.486649396	-0.179897039	C	-0.894963353	-0.145091789	-1.068926462
H	-0.937211902	-2.502153968	-0.815830502	C	-2.053751109	0.662679474	-1.082973719
C	-0.410181361	-1.670897840	1.380829332	C	-2.098699005	1.867963672	-1.777571049
H	-1.055042801	-1.217090611	2.132823160	H	-2.999488833	2.476136726	-1.770903230
H	-0.331423330	-2.746245234	1.533257822	C	-0.986840807	2.283674829	-2.496277481
C	1.157455929	0.621601403	1.247840684	H	-1.008191727	3.225106830	-3.037196131
C	2.485494082	0.854878243	0.839306890	C	0.150162813	1.482753068	-2.532913327
C	3.068593330	2.105406949	0.974083752	C	0.186994053	0.280071254	-1.836200957
H	4.092336409	2.283603244	0.657703740	H	1.087305719	-0.324700729	-1.880691217
C	2.315247058	3.145887621	1.510538589	C	-2.731563697	-1.230527411	0.155223898
H	2.755962254	4.132914626	1.612344618	C	-3.143019630	0.023731439	-0.339680715
C	0.999829979	2.933394019	1.904957168	C	-4.434162160	0.499566134	-0.126427526
C	0.424581090	1.670902040	1.777855699	H	-4.737371648	1.470341330	-0.509632822
H	-0.608412237	1.511909884	2.072434416	C	-5.339895141	-0.278768574	0.582599412
C	2.127009825	-1.419594897	0.269059125	H	-6.350014946	0.082025329	0.752875120
C	3.068579816	-0.369587367	0.255784729	C	-4.952087768	-1.521712797	1.076797720
C	4.333600167	-0.550836033	-0.282208628	C	-3.655921752	-1.984513827	0.870238403
H	5.058431097	0.257929703	-0.291859563	H	-3.361865082	-2.949753323	1.276012881
C	4.665836687	-1.789282057	-0.826134479	H	0.418887732	3.757922045	2.306110419
H	5.651197604	-1.940691531	-1.256401703	H	4.014270556	-3.785812373	-1.266419789
C	3.744534959	-2.829392296	-0.829330474	H	-5.666057558	-2.127725990	1.627985865
C	2.477676547	-2.643507099	-0.277250374	H	1.016384518	1.801450589	-3.105504564

5. References

S1 A. Hübner, M. Diefenbach, M. Bolte, H.-W. Lerner, M. C. Holthausen and M. Wagner, *Angew. Chem. Int. Ed.*, 2012, **51**, 12514-12518.

S2 T. Kaese, H. Budy, M. Bolte, H.-W. Lerner and M. Wagner, *Angew. Chem. Int. Ed.*, 2017, **56**, 7546-7550.

S3 A. Hübner, M. Bolte, H.-W. Lerner and M. Wagner, *Angew. Chem. Int. Ed.*, 2014, **53**, 10408-10411.

S4 G. R. Fulmer, A. J. M. Miller, N. H. Sherden, H. E. Gottlieb, A. Nudelman, B. M. Stoltz, J. E. Bercaw and K. I. Goldberg, *Organometallics*, 2010, **29**, 2176-2179.

S5 T. Kaese, A. Hübner, M. Bolte, H.-W. Lerner and M. Wagner, *J. Am. Chem. Soc.*, 2016, **138**, 6224-6233.

S6 *X-AREA: Diffractometer Control Program System*, Stoe & Cie, Darmstadt, Germany, 2002.

S7 G. M. Sheldrick, *Acta Crystallogr., Sect. A: Found. Crystallogr.*, 2008, **64**, 112-122.

S8 M. J. Frisch, G. W. Trucks, H. B. Schlegel, G. E. Scuseria, M. A. Robb, J. R. Cheeseman, G. Scalmani, V. Barone, B. Mennucci, G. A. Petersson, H. Nakatsuji, M. Caricato, X. Li, H. P. Hratchian, A. F. Izmaylov, J. Bloino, G. Zheng, J. L. Sonnenberg, M. Hada, M. Ehara, K. Toyota, R. Fukuda, J. Hasegawa, M. Ishida, T. Nakajima, Y. Honda, O. Kitao, H. Nakai, T. Vreven, J. A. Montgomery, Jr., J. E. Peralta, F. Ogliaro, M. Bearpark, J. J. Heyd, E. Brothers, K. N. Kudin, V. N. Staroverov, R. Kobayashi, J. Normand, K. Raghavachari, A. Rendell, J. C. Burant, S. S. Iyengar, J. Tomasi, M. Cossi, N. Rega, J. M. Millam, M. Klene, J. E. Knox, J. B. Cross, V. Bakken, C. Adamo, J. Jaramillo, R. Gomperts, R. E. Stratmann, O. Yazyev, A. J. Austin, R. Cammi, C. Pomelli, J. W. Ochterski, R. L. Martin, K. Morokuma, V. G. Zakrzewski, G. A. Voth, P. Salvador, J. J. Dannenberg, S. Dapprich, A. D. Daniels, Ö. Farkas, J. B. Foresman, J. V. Ortiz, J. Cioslowski and D. J. Fox, *Gaussian 09*, Revision D.01, Gaussian, Inc., Wallingford, CT, 2013; http://www.gaussian.com.

S9 J. P. Perdew, K. Burke and M. Ernzerhof, *Phys. Rev. Lett.*, 1996, **77**, 3865-3868.

S10 J. P. Perdew, K. Burke and M. Ernzerhof, *Phys. Rev. Lett.*, 1997, **78**, 1396-1396.

S11 J. P. Perdew, M. Ernzerhof and K. Burke, *J. Chem. Phys.*, 1996, **105**, 9982-9985.

S12 C. Adamo and V. Barone, *J. Chem. Phys.*, 1999, **110**, 6158-6170.

S13 L. Goerigk and S. Grimme, *J. Chem. Theory Comput.*, 2011, **7**, 291-309.

S14 S. Grimme, S. Ehrlich and L. Goerigk, *J. Comput. Chem.*, 2011, **32**, 1456-1465.

S15 A. Schäfer, C. Huber and R. Ahlrichs, *J. Chem. Phys.*, 1994, **100**, 5829-5835.

S16 A. V. Marenich, C. J. Cramer and D. G. Truhlar, *J. Phys. Chem. B*, 2009, **113**, 6378-6396.

S17 C. Y. Legault, *CYLview*, 1.0b; Université de Sherbrooke, Canada, 2009; http://www.cylview.org.
